# NMFProfiler: a multi-omics integration method for samples stratified in groups

**DOI:** 10.1093/bioinformatics/btaf066

**Published:** 2025-02-08

**Authors:** Aurélie Mercadié, Éléonore Gravier, Gwendal Josse, Isabelle Fournier, Cécile Viodé, Nathalie Vialaneix, Céline Brouard

**Affiliations:** Recherche & Développement, Pierre Fabre Dermo-cosmétique, Toulouse 31300, France; Université de Toulouse, INRAE, UR MIAT, Castanet-Tolosan Cedex 31326, France; Recherche & Développement, Pierre Fabre Dermo-cosmétique, Toulouse 31300, France; Recherche & Développement, Pierre Fabre Dermo-cosmétique, Toulouse 31300, France; Université de Lille, Inserm, CHU Lille, U1192 PRISM, Lille 59000, France; Recherche & Développement, Pierre Fabre Dermo-cosmétique, Toulouse 31300, France; Université de Toulouse, INRAE, UR MIAT, Castanet-Tolosan Cedex 31326, France; Université de Toulouse, INRAE, UR MIAT, Castanet-Tolosan Cedex 31326, France

## Abstract

**Motivation:**

The development of high-throughput sequencing enabled the massive production of “omics” data for various applications in biology. By analyzing simultaneously paired datasets collected on the same samples, integrative statistical approaches allow researchers to get a global picture of such systems and to highlight existing relationships between various molecular types and levels. Here, we introduce NMFProfiler, an integrative supervised NMF that accounts for the stratification of samples into groups of biological interest.

**Results:**

NMFProfiler was shown to successfully extract signatures characterizing groups with performances comparable to or better than state-of-the-art approaches. In particular, NMFProfiler was used in a clinical study on atopic dermatitis (AD) and to analyze a multi-omic cancer dataset. In the first case, it successfully identified signatures combining known AD protein biomarkers and novel transcriptomic biomarkers. In addition, it was also able to extract signatures significantly associated to cancer survival.

**Availability and implementation:**

NMFProfiler is released as a Python package, **NMFProfiler** (v0.3.0), available on PyPI.

## 1 Introduction

The development of high-throughput sequencing enabled the massive production of “omics” data, for various applications in biology. Generally collected on a same set of samples, each omic illustrates a reduced part of the overall functioning of complex biological systems. By simultaneously analyzing these datasets, integrative statistical approaches allow researchers to get a global picture of such systems and to highlight existing relationships between various molecular types and levels. On the one hand, integrative exploratory approaches, called *unsupervised methods* (Meng *et al.* 2016, Eicher *et al.* 2020), identify possible interactions between omics. On the other hand, predictive approaches, called *supervised methods* (Ritchie *et al.* 2015, Eicher *et al.* 2020), leverage molecular interactions to predict a phenotype of interest. Here, we tackle both problems at the same time: interactions between omics are analyzed to extract typical signatures made of interacting biomarkers, while simultaneously explaining a given stratification of the samples into “groups”. This stratification can correspond, *e.g.* to a clinical characteristic of samples that is of biological importance and signatures would thus inform on the specific functioning, at different omics levels, of the groups.

This “*mixed* problem” (also known as “joint association and classification problem”), has been much less studied in the literature than the supervised and unsupervised settings. A majority of the approaches tackling this issue are based on canonical correlation analysis (CCA) (Witten and Tibshirani 2009, Singh *et al.* 2019, Moon *et al.* 2022, Safo *et al.* 2022, Zhang and Gaynanova 2022). For example, DIABLO (Singh *et al.* 2019) is based on the sparse generalized CCA (sGCCA) (Tenenhaus *et al.* 2014) and seeks projections maximizing a criterion of covariance between omics pairs and clinical data. JACA and SIDA (Safo *et al.* 2022, Zhang and Gaynanova 2022) mix CCA and linear discriminant analysis (LDA) in order to find correlated omics that discriminate well a phenotype. Closely related to this framework, SDGCCA (Moon *et al.* 2022) is a nonlinear variant of sGCCA based on deep neural networks but, due to its deep learning ground, it might be not adapted to small-size samples, frequent in clinical studies. Lastly, Ding *et al.* (2022) introduced an approach called cooperative learning that is more oriented toward the prediction quality of both omics independently but that nevertheless includes a term enforcing the omics-specific predictions to agree.

The nonnegative matrix factorization (NMF) is a well-known dimension reduction method introduced by Lee and Seung (1999). This method was developed to analyze nonnegative data and is thus well adapted to most omics datasets (*e.g.* count data from sequencing techniques as transcriptomics or metagenomics; compositional data as for metabolomics or proteomics; etc.). The nonnegativity of the solution has appealing interpretability, compared to partial least squares regression or factorial analysis. Hence, some recent variants have been designed to analyze omics or biological data for unsupervised (Zhang *et al.* 2011, Yang and Michailidis 2016, Chalise and Fridley 2017, Moon and Lee 2021, Pierre-Jean *et al.* 2022) or (semi-)supervised (Gaujoux and Seoighe 2012, Cai *et al.* 2017, Chao *et al.* 2018, Leuschner *et al.* 2019) problems. However, even though both supervised and integrative NMF have shown their ability to successfully solve unsupervised or supervised problems in biology (Rappoport and Shamir 2018, Chauvel *et al.* 2020, Pierre-Jean *et al.* 2020, Cantini *et al.* 2021), to the best of our knowledge, they have never been combined to address mixed problems.

Here, we introduce NMFProfiler, a mixed integrative NMF. NMFProfiler combines ideas from integrative and supervised NMF but is based on a novel supervised term that is more adapted to the nonnegative setting of the NMF than the one proposed in previous supervised NMF (Leuschner *et al.* 2019). A new proximal optimization approach is also proposed to get exact sparsity in obtained signatures. Its relevance is illustrated on simulated datasets, on a TCGA dataset, and on a clinical study of atopic dermatitis (AD).

## 2 Materials and methods

In the following, we consider J omics datasets, $X^{\left(j\right)}\in R_{+}^{n\times p_{j}}$ (j∈{1,…,J}). Omics are both measured on the same n samples but by different types of features ($p_{j}$ features, respectively). In addition, samples are known to belong to one of two groups, identified by a binary vector $y\in {\left\{0,1\right\}}^{n}$ (or by its one-hot encoding form $Y\in {\left\{0,1\right\}}^{n\times 2}$). Note that, for the sake of clarity, the presentation of the method is done for U=2 groups but its extension to more than two groups is straightforward and briefly discussed at the end of Section 2.2.

### 2.1 Standard NMF and existing extensions

First consider a single matrix $X\in R_{+}^{n\times p}$, in which the number of features can be much larger than the sample size (n≪p). The NMF (Lee and Seung 1999) produces a low rank approximation of X, in which X is decomposed into two nonnegative matrices X≃WH, with $W\in R_{+}^{n\times K}$, $H\in R_{+}^{K\times p}$, where K is the chosen number of signatures. Given K, both W (“contribution matrix”) and H (“signature matrix”) are obtained by solving a minimization problem that measures the quality of the approximation


(1) $$\underset{W,H\geq 0}{\text{arg min}}L(X,\mathrm{WH}),$$

where the loss function L is generally taken as $||X-\mathrm{WH}|{|}_{F}^{2}$.

*Extension of NMF for classification problems (supervised NMF “FR-lda”).* When a binary vector $y\in {\left\{0,1\right\}}^{n}$ characterizes groups of individuals, the supervised NMF of Fernsel and Maass (2018) proposes to add a second loss term to the reconstruction loss of Equation (1). This loss uses the projection of the original data onto the signature matrix, H, as a predictor in a linear regression setting and leads to solving this minimization problem:


(2) $$\underset{W,H,\beta \geq 0}{\text{arg min}}L(X,\mathrm{WH})+\frac{\gamma }{2}||y-XH^{⊤}\beta |{|}_{2}^{2},$$

where $\beta \in R_{+}^{K}$ corresponds to LDA-like regression coefficients and γ≥0 controls the tradeoff between the reconstruction loss and the supervised loss. The authors called the approach the “FR-lda” variant of the NMF.

This first method was later improved for better interpretability by Leuschner *et al.* (2019) who introduced a $\ell_{1}$- and $\ell_{2}$-regularized version of Problem (2): ${\text{arg min}}_{W,H,\beta \geq 0}L(X,\mathrm{WH})+\frac{\gamma }{2}||y-XH^{⊤}\beta |{|}_{2}^{2}+\lambda ||H|{|}_{1}+\frac{\mu }{2}||W|{|}_{F}^{2}+\frac{\nu }{2}||H|{|}_{F}^{2}$, where λ,μ,ν>0 are given regularization hyperparameters. The $\ell_{1}$-regularization term ensures the sparsity of obtained signatures and $\ell_{2}$ penalties aim to improve the identifiability of the decomposition.

*Extension of NMF to multi-table problems.*  Zhang *et al.* (2012) extended the NMF to integrate all sources of information in a joint NMF (jNMF). In this method, table-specific dictionaries, or signatures, $H^{\left(j\right)}$, are obtained but forced to describe a common sample contribution matrix W: ${\text{arg min}}_{W,H^{\left(1\right)},\ldots ,H^{\left(J\right)}\geq 0}\sum_{j=1}^{J}||X^{\left(j\right)}-WH^{\left(j\right)}|{|}_{F}^{2}$.

### 2.2 NMFProfiler: a mixed integrative NMF

Here, we propose a new NMF variant combining the advantages of the supervised NMF and of jNMF that we name “NMFProfiler.” As in jNMF, W contains the common contributions of individuals to the omic-specific dictionaries, or signatures, $H^{\left(j\right)}$, which are each driven to discriminate one of the groups by a LDA-type loss. However, our proposal is not a direct plug-in of the LDA-criterion of the supervised FR-lda into jNMF. Instead, we derive a criterion equivalent to K independent linear regressions, one for each group (so, here K=2). Details on the differences between the LDA term of the supervised FR-lda of Fernsel and Maass (2018) and our criterion are given in Supplementary Section S1 of Supplementary Material.

NMFProfiler is set to solve the following optimization problem:


(3) $$\underset{W,H^{\left(1\right)},\ldots ,H^{\left(J\right)},\beta^{\left(1\right)},\ldots ,\beta^{\left(J\right)}\geq 0}{\text{arg min}}F\left(W,{\left\{H^{\left(j\right)}\right\}}_{j=1}^{J},{\left\{\beta^{\left(j\right)}\right\}}_{j=1}^{J}\right),$$

where $F\left(W,{\left\{H^{\left(j\right)}\right\}}_{j=1}^{J},{\left\{\beta^{\left(j\right)}\right\}}_{j=1}^{J}\right)$ is equal to


(4) $$\begin{array}{cc} \frac{1}{2}\sum_{j=1}^{J}||X^{\left(j\right)}-WH^{\left(j\right)}|{|}_{F}^{2} & +\frac{\gamma }{2}\sum_{j=1}^{J}||Y-X^{\left(j\right)}H^{(j)⊤}\text{Diag}\left(\beta^{\left(j\right)}\right)|{|}_{F}^{2}\\ & +\sum_{j=1}^{J}\lambda ||H^{\left(j\right)}|{|}_{1}+\frac{\mu }{2}||W|{|}_{F}^{2} \end{array}$$

with $\text{Diag}\left(\beta^{\left(j\right)}\right)$, the 2×2 diagonal matrix with diagonal entries equal to $\beta^{\left(j\right)}\in R_{+}^{2}$. jNMF is a specific instance of this problem that corresponds to the case γ=λ=μ=0.

The criterion of Equation (4) can be extended in a trivial way to more than two groups: For U groups, K=U signatures are extracted and the regression part of the loss (the second term) is modified into a multivariate regression problem with K dimensions.

### 2.3 Solving the optimization problem

The optimization problems of NMF are described as “ill-posed, nonlinear, and nonconvex” (Fernsel and Maass 2018) because F is not simultaneously convex in W, $H^{\left(j\right)}$, and $\beta^{\left(j\right)}$. However, they can be written as separate convex optimization problems in each feature, one of them including a nonsmooth constraint. This is solved using alternating algorithms using a gradient descent approach. Fernsel and Maass (2018) describe updates of W, $H^{\left(j\right)}$, and $\beta^{\left(j\right)}$ leading to Multiplicative Updates (MU), which ensure positivity of the estimated matrices.

We introduce a new optimization of Equation (3) that yields exact (and not approximate, contrary to MU updates) sparsity on $H^{\left(j\right)}$ by a proximal approach (NMFProfiler-prox). Details on this proximal optimization are described in Supplementary Section S2 of the Supplementary Material. Both variants are implemented in the Python package NMFProfiler (v0.3.0) available from PyPI https://pypi.org/project/NMFProfiler. The source code of the package is available at https://forgemia.inra.fr/omics-integration/nmfprofiler.

### 2.4 Simulated datasets

NMFProfiler was first evaluated using J=2 simulated datasets. We used the same data generation process than the one described in Yang and Michailidis (2016) (scripts are available at https://github.com/yangzi4/iNMF/tree/master) because these data had previously also been used to test the integrative NMF approach (iNMF) of Yang and Michailidis (2016) as well as to assess the relevance of unsupervised multi-omics methods to cluster samples in the benchmark article of Chauvel *et al.* (2020).

**Figure 1.**
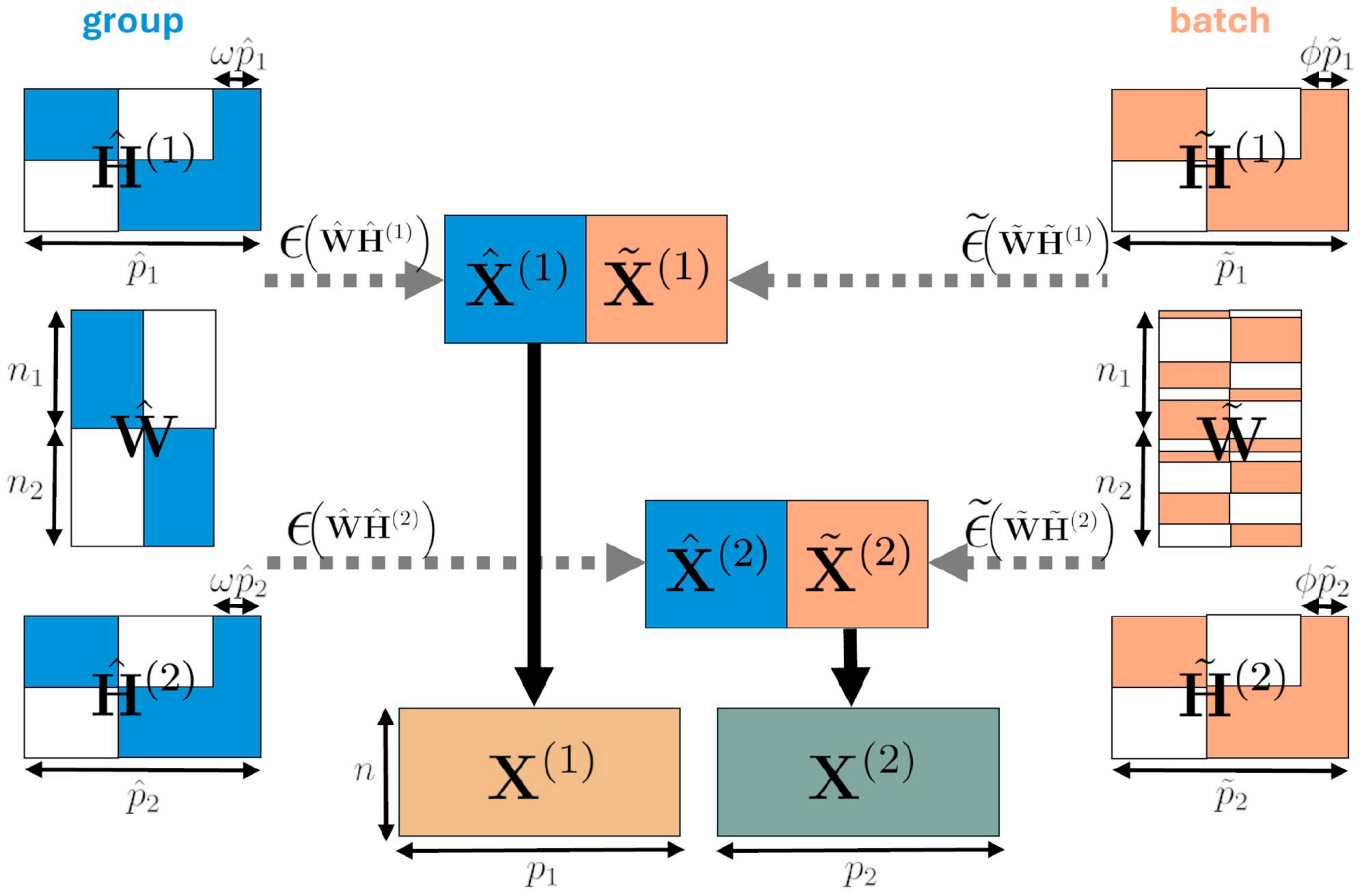
Data generation model for “Simulated datasets”. Colored blocks contain positive values. White blocks contain null values. ω (resp. ϕ) controls the number of noisy features inserted in ${\hat{H}}^{\left(j\right)}$ matrices for “group” (resp. ${\tilde{H}}^{\left(j\right)}$ matrices for “batch”). ϵ() and $\tilde{\epsilon }()$ are processes introducing noise. Some parameters are fixed in all simulations: n=50, $p_{1}=2500$, $p_{2}=400$, and K=2.

To generate simulated data with a clear ground truth, binary matrices stratified by groups, W and $H^{\left(j\right)}$ (∀j∈{1,2}), were first generated from K=2 signatures for each omic and used (together with different types of noise ϵ) to generate data matrices $X^{\left(j\right)}$. A realistic batch noise was also introduced using two datasets (${\tilde{X}}^{\left(j\right)}$) simulated independently and similarly but stratified by another type of group (called “batch” effect) independent of the “true” group structure of W and $H^{\left(j\right)}$. The final dataset was obtained as the concatenation of the columns of the two datasets. Figure 1 illustrates the data generation process and details on this process are provided in Supplementary Section S3.1 of Supplementary Material.

The flexible framework of this data generation procedure allowed us to vary different parameters of the simulations (*e.g.* number of features not selected in either of the K=2 signatures from the true group structure or the batch effect) but the results presented in this article mainly corresponds to one simulation with $n_{1}=n_{2}=n/2$, ${\hat{p}}_{1}={\tilde{p}}_{1}=p_{1}/2$, ${\hat{p}}_{2}={\tilde{p}}_{2}=p_{2}/2$, ω=ϕ=0, all features corresponding to a group or batch signature and with a larger variance for the “true” group datasets. An example of obtained datasets is given in Supplementary Fig. S3 of the Supplementary Material. The other tested simulation designs are described in Supplementary Section S3.1 of Supplementary Material and exhaustive results are available in Supplementary Sections S5.1.1–S5.1.5 of Supplementary Material.

### 2.5 TCGA

Similarly to ideas presented in Rappoport and Shamir (2018) and Cantini *et al.* (2021), we also evaluated NMFProfiler on TCGA multi-omics data. More precisely, we obtained three omics for colon adenocarcinoma (COAD) (gene expression, DNA methylation, and miRNA expression), measured for n=221 samples.

We evaluated NMFProfiler ability to integrate more than two omics in association with clinical labels with more than two levels (groups) previously used in Rappoport and Shamir (2018): pathologic T, pathologic M, pathologic N (respectively, measuring the progression of the tumor, metastases, and cancer in lymph nodes and noted T, M, and N, respectively). Clinical labels were recoded in three groups (respectively, {T2, T3, T4}, {M0, M1, MX} and {N0, N1, N2}) and subsets of the original dataset corresponding to binary contrasts of these variables (respectively, T2vsT3, T2vsT4, M0vsM1, M0vsMX, N0vsN1, and N0vsN2) were also considered, for the sake of comparison with DIABLO. Further information on data preprocessing is described in Supplementary Section S3.2 of Supplementary Material.

### 2.6 Proteomic and transcriptomic study on atopic dermatitis

NMFProfiler was also used on transcriptomic (microarray) and proteomic (LCMS) data obtained from a study on AD in nonlesional skin. AD is a common inflammatory skin disease, characterized mainly by an impaired-skin barrier function. Impairment of skin barrier function is responsible for increased penetration of environmental allergens into the skin and initiates immunological response and inflammation. Lesional AD skin has been investigated in several studies using transcriptomic or proteomic approaches (Cole *et al.* 2014, Sakabe *et al.* 2014, Ghosh *et al.* 2015), but it is less the case for nonlesional AD skin. Both datasets were obtained on n=12 volunteers, comprising five AD subjects and seven healthy volunteers. Suction blisters were sampled from these subjects’ interior forearms. Further information on data, including preprocessing steps, is described in Supplementary Section S3.2 of Supplementary Material. The final obtained datasets contained *p*_1_ = 1847 probeset genes and *p*_2_ = 281 proteins.

### 2.7 Comparison with other integrative approaches

To assess the relevance of NMFProfiler, we compared both versions (NMFProfiler-MU and NMFProfiler-prox) with other state-of-the-art methods for omics data integration:

- *jNMF* (Zhang *et al.* 2012): We used our implementation to perform jNMF, simply setting γ of Equation (4) to 0 (This actually corresponds to a $\ell_{2}$-regularized version of the original jNMF approach.). The two solvers (MU or proximal) led to two different variants: jNMF-MU and jNMF-prox;
- *DIABLO* (Singh *et al.* 2019): We used the R package **mixOmics** (v6.20.0) that builds on sGCCA (Tenenhaus *et al.* 2014). We used both the sparse and nonsparse versions of the method: DIABLO and DIABLO-nonsparse;
- *MOFA* (Argelaguet *et al.* 2018): We used the R package **MOFA2** (v1.6.0).

jNMF and MOFA were only assessed on simulated datasets and real-case studies (TCGA-COAD and AD) focused on the two most efficient methods, DIABLO and NMFProfiler.

In all methods, we selected a number of signatures K corresponding to the number of groups of individuals: K=2 or K>2 for NMF variants and K=1 for DIABLO variants and MOFA, split in two based on signs. For U=K=2, relations between signatures and groups were automatically derived from the average of estimated W by groups (NMF) or similarly from the variate matrix (other methods). For cases with more than two groups (in TCGA-COAD), relations between signatures and groups were obtained similarly for NMF but could not be obtained for DIABLO methods. Indeed, DIABLO extracts loadings that characterize all groups simultaneously and there is no simple automatic method to partition K global loadings into U (U>2) group-specific signatures, whatever the number K. Hence, for TCGA-COAD, DIABLO was only trained for cases where the number of groups was exactly equal to two. Further information on method implementation are provided in Supplementary Section S4 of Supplementary Material.

Methods were compared using different quality criteria. We evaluated their ability to recover correct signatures composition when a ground truth was available (*e.g.* on simulated data) using the rank of features correctly/incorrectly included in the signatures with Receiver Operating Characteristic (ROC) curves and the Area Under this curve (AUROC). We evaluated their ability to provide signatures predictive of the groups by performing a logistic regression of y on $X^{\left(j\right)}{\left({\hat{H}}^{\left(j\right)}\right)}^{⊤}$ with a 5-fold CV estimation of the classification accuracy and of the McFadden index (also called pseudo-$R^{2}$). We evaluated the exact sparsity of signatures (for sparse methods). The stability of obtained conclusions was assessed by repeating the simulation process 50 times. Finally, similarly to Cantini *et al.* (2021), in TCGA-COAD, we evaluated the predictive power of signatures for survival prediction. In each of the group, we fitted a Cox proportional hazard regression with the projection of samples onto signatures as predictors and assessed the significance of the model as well as that of each of the omic signature.

## 3 Results

### 3.1 Simulated data

#### 3.1.1 Method comparison

**Figure 2.**
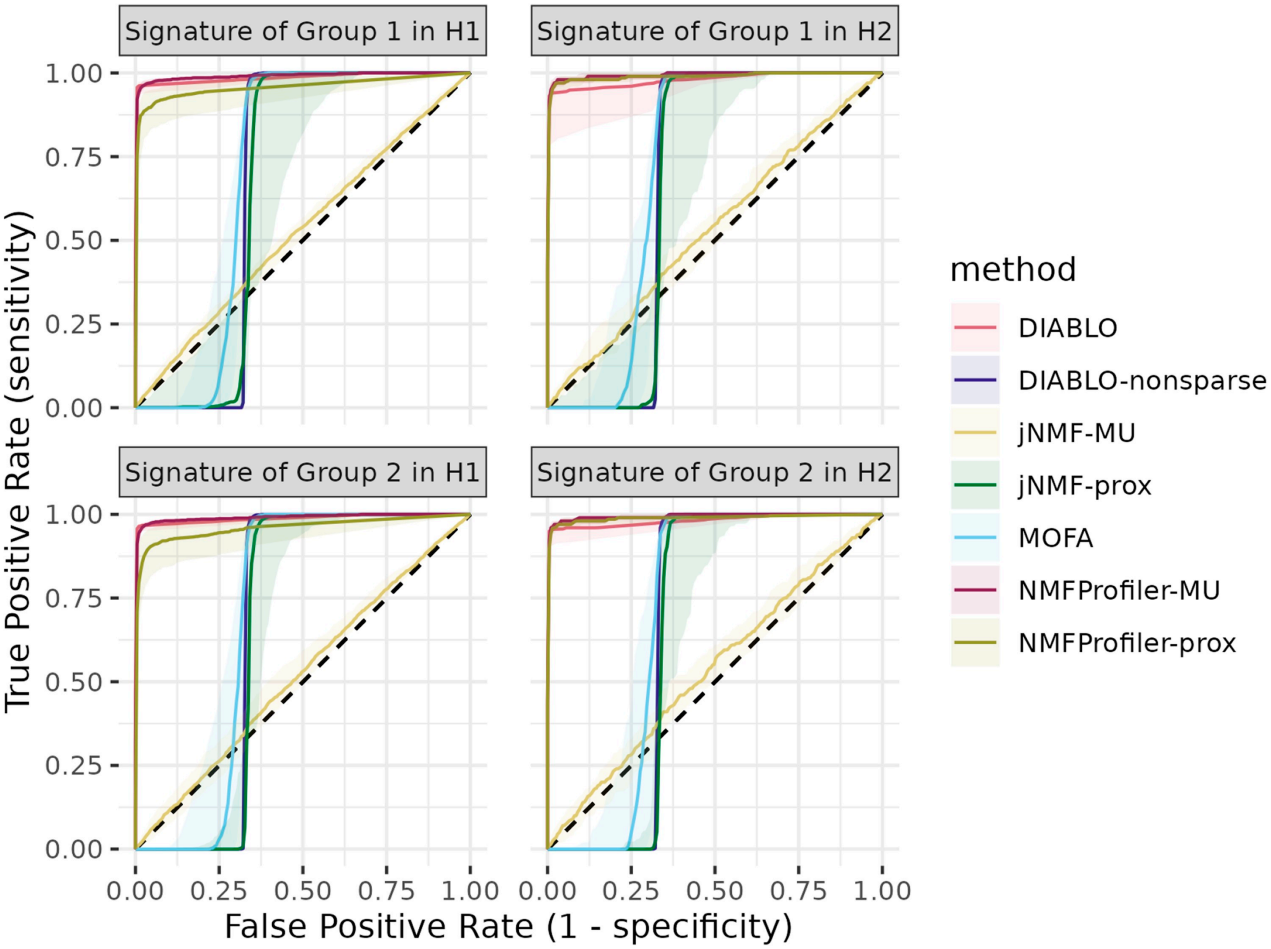
Simulated dataset. Median ROCs for all methods. The ribbon corresponds to interquartile range over the 50 simulations. The dashed line corresponds to the ROC of a random classifier.

Methods were first compared on the simulated data as generated by simulation settings described in Section 2.3. We started by assessing their ability to retrieve the ground truth features characteristic of each group. Based on feature ranking of each method, ROC curves were obtained. Figure 2 and Table 1, respectively, give the median ROC curve (with a ribbon indicating the range of the ROC curves), the average area under the ROC curve of each method, and its standard deviation (SD) over the 50 simulations. As expected, results indicate that supervised methods (DIABLO and NMFProfiler) had better performances than unsupervised methods (jNMF and MOFA). Indeed, unsupervised methods extract information related to the main source of variability, which works well if the variability is well explained by the group but fails when external covariates (here, the batch features) are the main drivers of the variability.

**Table 1.** Simulated dataset.^a^

*j*	*u*	AUROC						
		jNMF		MOFA	DIABLO		NMFProfiler	
		MU	prox	nonsparse	nonsparse	sparse	MU (nonsparse)	prox (sparse)
1	1	0.541 (0.074)	0.647 (0.115)	0.720 (0.096)	0.675 (0.010)	**0.974** (0.062)	**0.990** (0.005)	0.946 (0.048)
1	2	0.538 (0.063)	0.640 (0.111)	0.712 (0.092)	0.673 (0.006)	**0.978** (0.044)	**0.990** (0.004)	0.942 (0.059)
2	1	0.543 (0.074)	0.650 (0.106)	0.721 (0.095)	0.673 (0.007)	0.971 (0.064)	**0.991** (0.008)	**0.985** (0.021)
2	2	0.543 (0.064)	0.648 (0.101)	0.712 (0.088)	0.671 (0.005)	0.974 (0.047)	**0.991** (0.008)	**0.987** (0.010)

a Mean (SD) AUROCs. j∈{1,2} stands for the omic/dataset number and *u* for the group number. Bold numbers correspond to the best performance for a given omic, a given group, and a given type of method (sparse or nonsparse).

Among supervised methods, NMFProfiler-MU systematically had the highest average AUROC, while the nonsparse version of DIABLO had very poor results. DIABLO seemed to be very sensitive to the proper setting of the number of selected features, which was done with a meticulous tuning for the sparse version, but at the cost of a large increase of computational time (10 s for NFMProfiler-prox on average versus 680 s for DIABLO; see Supplementary Fig. S12 of Supplementary Material). Finally, the proximal version of NMFProfiler gave slightly lower performances than the MU version: The better interpretability of the proximal version comes at the cost of a slightly deteriorated prediction ability (see also individual ROC curves of the 50 simulations in Supplementary Figs S4–S6 of Supplementary Material and the discussion of Section 3.1.3 below).

We then evaluated the ability of the methods to provide signatures predictive of the groups by performing a logistic regression of y onto $X^{\left(j\right)}{\left({\hat{H}}^{\left(j\right)}\right)}^{⊤}$. Here, we focused only on the most effective supervised methods, DIABLO, NMFProfiler-prox, and NMFProfiler-MU. Mean accuracy and McFadden index were computed using a 5-fold CV. Overall, results showed that DIABLO and NMFProfiler-prox, which produce direct sparse signatures, had an higher explanatory power and accuracy when classifying samples than NMFProfiler-MU (Supplementary Figs S10 and S11 of Supplementary Material), with a slight advantage for DIABLO on the accuracy.

We also assessed the influence of the batch pattern size, the level of noisy features inside datasets or even group disequilibrium on all methods (see Supplementary Table S1 of Supplementary Material for a complete description of all setting variations). Results for these variants are available in Supplementary Sections S5.1 and S5.2 of Supplementary Material. In these simulations, unsupervised methods and DIABLO-nonsparse showed a diminished ability to select relevant features as the proportion of batch features inside data increases. NMFProfiler-prox was found sensitive to high levels of noise or to large numbers of irrelevant features, which was not the case for DIABLO variants, and to a lesser extent NMFProfiler-MU. In addition, NMFProfiler variants and DIABLO showed robustness against unbalanced group samples, with a slight advantage to DIABLO in cases where there is no batch feature.

#### 3.1.2 Impact of the new supervised term

**Figure 3.**
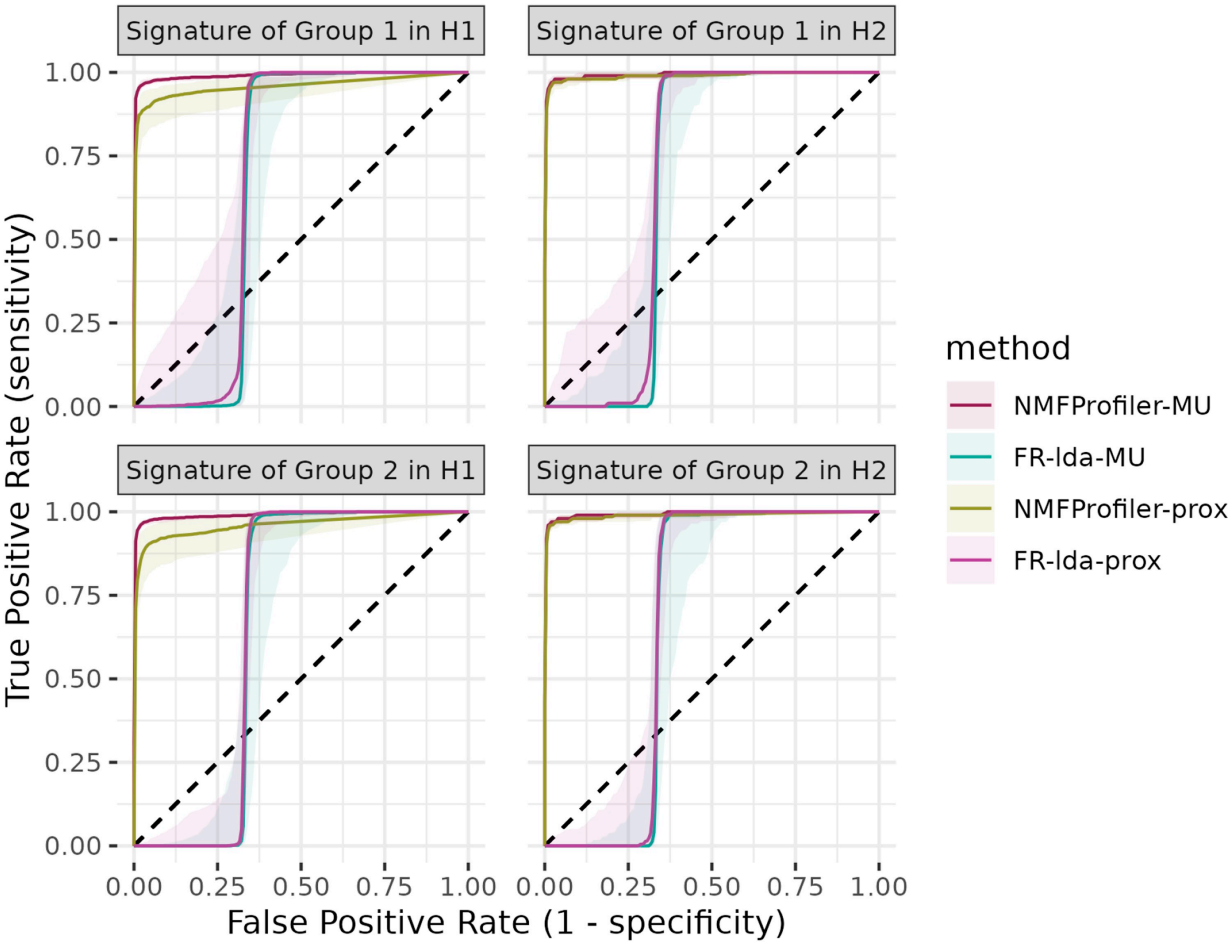
Simulated datasets. Median ROCs for variants of the supervised term in supervised NMF. The ribbon corresponds to interquartile range over the 50 simulations. The dashed line corresponds to the ROC of a random classifier.

To assess the relevance of using the supervised term of Equation (4) instead of the original LDA-term of Leuschner *et al.* (2019), we also compared both versions of the supervised NMF on the same simulated dataset. Figure 3 shows the median ROC curves of the MU and sparse versions of NMFProfiler and FR-lda. In this simulation setting, FR-lda failed to properly use the supervised term to select relevant features and had results comparable to the unsupervised methods jNMF-prox and MOFA. In other simulations with no batch noise (*e.g.* simulation setting n°05 in Supplementary Table S1), NMFProfiler and FR-lda displayed similar performances (see Supplementary Fig. S18) because, similarly to unsupervised methods, the FR-lda version is able to extract the main source of variability (which, in this case, is the group).

#### 3.1.3 Assessment of the sparsity level

Another important aspect of the methods is their ability to select true features in a clear and automatic way and thus to ease result interpretation. Supplementary Figures S7 and S8 of Supplementary material display the specificity (proportion of predicted true zeros among ground truth irrelevant features) of the NMFProfiler and DIABLO variants, respectively. Note that, contrary to ROC curves displayed in previous section, these values correspond to the direct output of the method, without additional thresholding. In addition, sensitivity was displayed separately for DIABLO and NMFProfiler variants because their values are not directly comparable. A signature in NMFProfiler is specific of one group, while a loading in DIABLO is describing the two groups simultaneously. Hence, the number of ground truth irrelevant features is higher for NMFProfiler than for DIABLO (because, for a given signature, it includes the features relevant for the other group) and the sensitivity is thus expected to be smaller by design.

As expected, only NMFProfiler-prox and DIABLO have exact sparsity and thus positive specificity. Levels of specificity were good, even though higher for the first dataset (with more features), and higher for DIABLO. More directly comparing the signature coefficients obtained by NMFProfiler-prox and NMFProfiler-MU, we also found that NMFProfiler-prox predicted higher coefficients for relevant features (Supplementary Fig. S9 of Supplementary Material) and that it generally automatically obtained the sparsity level maximizing the true positive rate (TPR) (Supplementary Fig. S6 of Supplementary Material).

### 3.2 NMFProfiler extracts signatures predictive of survival

Results are described only for groups designed as N0vsN1, N0vsN2, and N, corresponding to three types of recoding of pathologic N (the first two are binary recoding and the other is a recoding in three groups, only used with NMFProfiler). The other results are provided in Supplementary Section S5.3 of Supplementary Material.

**Figure 4.**
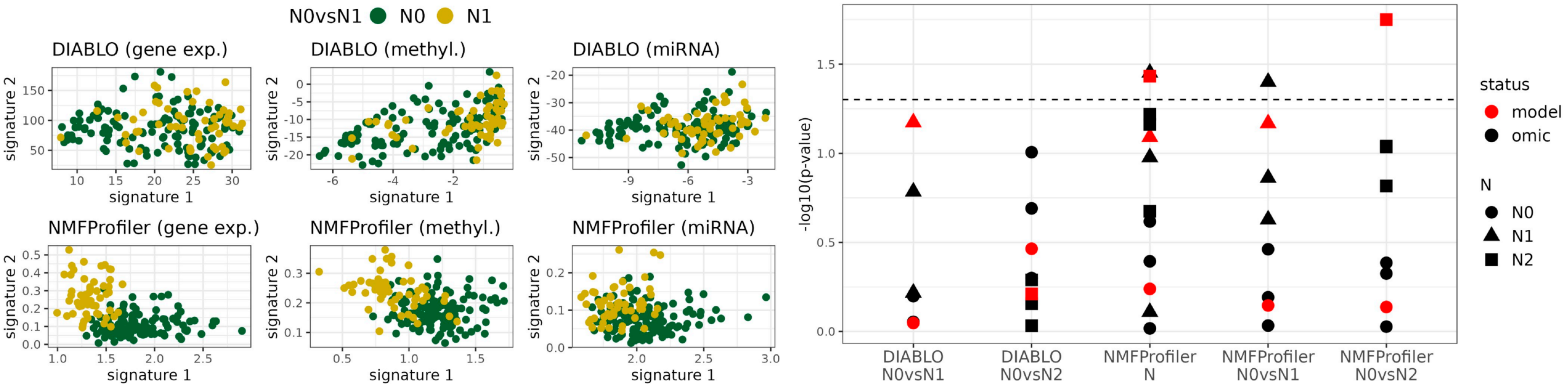
TCGA-COAD. Left: Projection of samples onto signatures obtained for N0vsN1 for each omic and method. For DIABLO, only the *x-axis (first signature) is relevant (split based on sign). Right*: $-{\;\text{log}\;}_{10}$(*P*-values) obtained with Cox proportional hazard models for the association of survival to both N0vsN1 and N0vsN2 signatures obtained by DIABLO and NMFProfiler. The full (versus null) model *P-*value is displayed in red and the three *P-*values corresponding to an omic-specific signature are displayed in black. The dashed horizontal line corresponds to a *P-*value of 0.05.

Figure 4 (left) displays the projection of samples onto signatures obtained with NMFProfiler-MU and DIABLO for N0vsN1. In this plot, NMFProfiler shows a much better ability than DIABLO to separate the two groups, especially for gene expression and DNA methylation. Similar results were obtained with groups coming from the other clinical features.

Figure 4 (right) shows the predictive significance of selected features (in terms of $-{\;\text{log}\;}_{10}p$-value) in survival prediction within groups (Cox proportional hazard regression). NMFProfiler was able to select signatures significantly associated with survival (one for N, with also one global model significantly associated to the survival, one for N0vsN1, and one global model also significantly associated for N0vsN2). In contrast, none of the signature selected by DIABLO was found significantly associated with survival in any of the groups. This result shows the relevance of our approach since finding signatures predictive of survival for colon adenocarcinoma has previously been reported to be rare and difficult in this cancer type (Rappoport and Shamir 2018, Cantini *et al.* 2021). Note that a similar result was also obtained for groups derived from pathologic M.

### 3.3 NMFProfiler successfully identifies molecular signatures of atopic dermatitis

**Figure 5.**
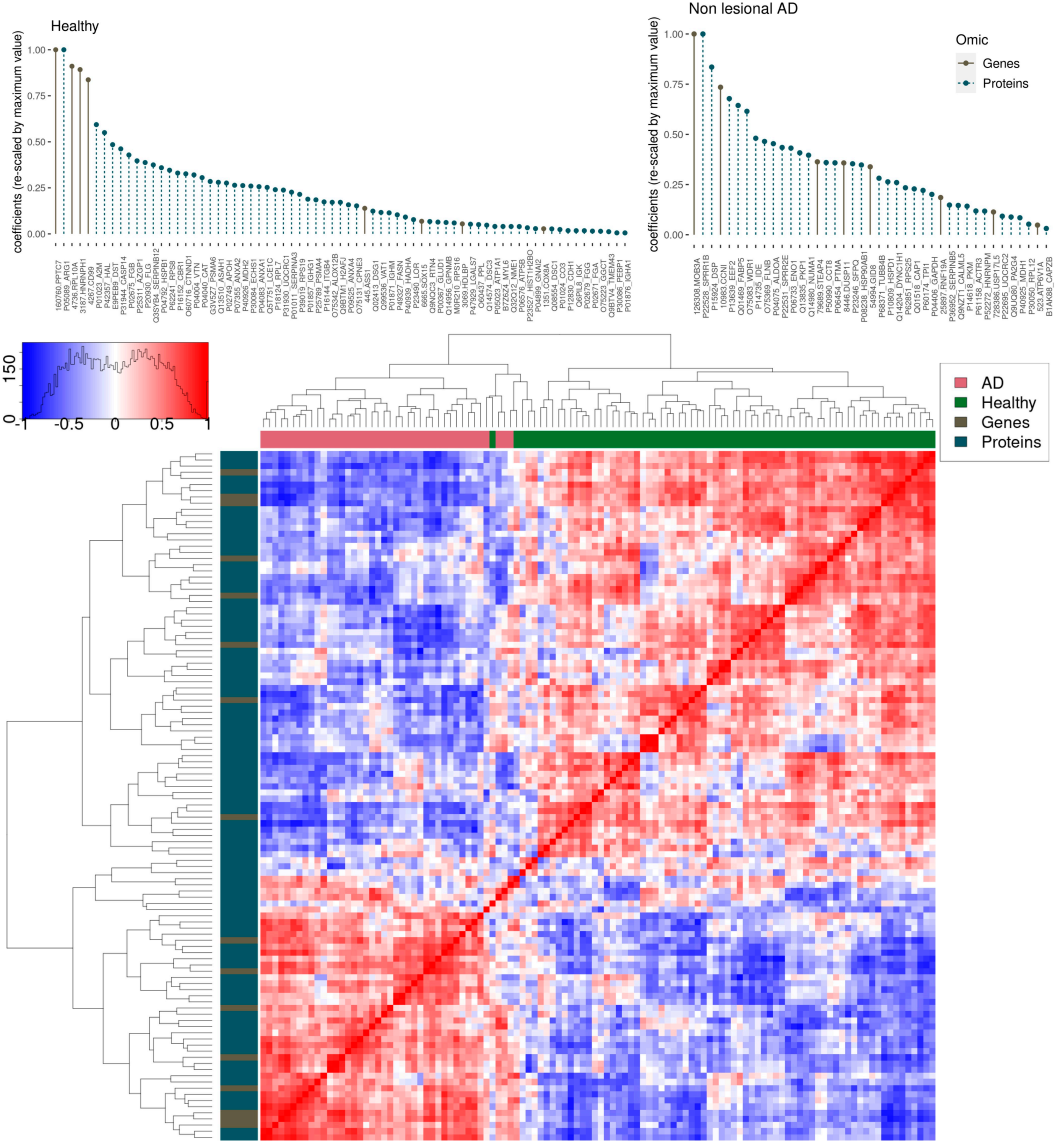
Atopic dermatitis. Top: Features selected by NMFProfiler-prox for both signatures (healthy: left; nonlesional AD: right), ordered by decreasing order of their coefficients in ${\hat{H}}^{\left(j\right)}$. To ease readability, coefficients have been rescaled so that their maximum is equal to 1. Bottom: Pearson correlation heatmap of selected features. Features are ordered identically in rows and columns, based on the result of a hierarchical clustering (complete linkage) with Euclidean distance. Colors displayed for rows (resp. columns) correspond to the molecular type (gene or protein) (resp. to the signature type: healthy or AD).

NMFProfiler-prox was used to extract molecular (genes and proteins) signatures of subjects with or without AD. Obtained signatures were sparse: 16 genes were selected from the transcriptomics dataset (over the 1847 genes initially available) and 96 proteins were selected from the proteomics dataset (over the 281 proteins initially available). Figure 5 (top) displays the positive coefficients of extracted features in both datasets for both sample groups (healthy samples and nonlesional AD samples) and the same figure (bottom) displays the Pearson correlation heatmap of selected features.

The molecular signatures of nonlesional AD subjects contained less features than the molecular signatures of healthy subjects. This is consistent with the well-known fact that AD skin is characterized by the down-regulation of genes/proteins relative to the skin barrier structure. More precisely, extracted signatures showed that the presence of Arginase-1 and Filaggrin is characteristic of healthy subjects and a complementary differential analysis revealed that these proteins were indeed significantly underexpressed for AD samples (adjusted *P*-values of $5.32\times 10^{-7}$ and $6.96\times 10^{-4}$, respectively, with moderated t-tests from **limma**; see Supplementary Table S1 and Supplementary Section S5.4 of Supplementary Material). Filaggrin, a skin barrier protein, is a well known biomarker of the AD pathogenesis (Nakajima *et al.* 2024) and Arginase-1 is a protein known to be related to skin natural moisturizing. Hence, decreasing levels of Filaggrin and Arginase-1 could reflect impaired skin barrier function, consistent with AD. Moreover, the presence of SPRR1B, SPRR2E, FABP5 or even of HSP90AB1 and HSPD1 proteins is known to be typical of nonlesional AD skin. Indeed, small proline-rich proteins (as SPRR1B, SPRR2E), implied in keratinization process, and fatty acid binding proteins (as FABP5), implied in fatty acid metabolism, were found to be highly expressed in nonlesional/lesional skin of AD and psoriatic patients (Nakajima *et al.* 2024, Rusinol and Puig 2024). Heat shock proteins (as HSP90AB1, HSPD1) play a role in inflammatory stress response, and when inhibited attenuates inflammation on AD samples (Ben Abdallah *et al.* 2023). Conclusions for selected genes are less clear but nine out of the 16 selected genes were also found to be significantly under/overexpressed by a differential analysis (moderated t-tests from **limma**; see Supplementary Table S1).

Obtained signatures were compared with the single signature extracted using DIABLO on the same dataset (see Supplementary Section S5.4 for further details): DIABLO signatures include 200 genes and 10 proteins. All proteins found in DIABLO signature were also found by NMFProfiler and a majority of genes selected by NMFProfiler were also found in DIABLO signature. Overall, NMFProfiler is less influenced by unbalanced sizes between the two datasets in its selection, while DIABLO tends to extract signatures with sizes more influenced by the respective initial number of features of the two omics. Also, as expected, DIABLO provides signatures with very strong correlations between features: In particular, DIABLO gene signature has an average (absolute value of) correlation equal to 0.682 while the (smaller) gene signatures of NMFProfiler are less redundant, with an average absolute value of correlations equal to 0.51. This is explained by the fact that the objective function of DIABLO, based on covariance, favors strong correlations between extracted features, while NMFProfiler seeks good reconstruction and better benefit from the $\ell_{1}$ penalty to extract nonredundant features. The same remark holds for the protein signature (average signature of 0.729 for DIABLO and of 0.369 for NMFProfiler) but this is a more expected result regarding the number of proteins selected by each method.

However, extracted signatures remain consistent: Figure 5 (bottom) confirms the existence of strong linear positive associations within, respectively, healthy and AD signatures and of negative associations between signatures. If the precise role of some of the identified molecules is still to be confirmed, NMFProfiler has been able to obtain results consistent with known biomarkers and has extracted potential new biomarkers. Hence, compared to standard analyses performed independently on each gene or protein (like differential analysis), NMFProfiler provides a complete signature of associated omics that potentially work together. It has thus the ability to include features that classical single-omic approaches would miss and to achieve a good tradeoff between complementarity and consistency of the features included in the signature.

## 4 Discussion

We developed an extension of the NMF able to find multi-omic signatures typical of groups of samples. The approach was successfully tested on simulated and real data.

On simulated data, we showed that NMFProfiler was able to retrieve a majority of the features characterizing groups specifically, classified well samples and ran fast. We were able to show that NMFProfiler compares similarly or favorably with state-of-the-art methods for omics integration. The simulated study also highlighted that the proximal solver that we proposed enables the recovery of signatures that are directly sparse, which is an advantage for the interpretation. However, this optimization solver was also shown to be less robust as the noise level increases than the more common MU solver. Both solvers are provided in our implementation, that can be chosen depending on the level of noise expected in the data.

NMFProfiler was able to extract relevant signatures in an AD multi-omics study: In addition to known protein AD biomarkers, it provided a list of new potential biomarker genes. In TCGA dataset study, it also extracted relevant signatures, significantly associated to survival, from groups based only on clinical information.

In terms of interpretability, NMFProfiler signatures are directly specific of a single group. This provides an advantage compared to other PCA- or CCA-like methods (*e.g.* DIABLO and MOFA) where extracted loadings are supposed to characterize simultaneously all groups. For PCA- and CCA-like methods, the set of variables contributing to a loading are thus to be re-interpreted a posteriori to obtain group-specific signatures: In the case of two groups, this can be done using a split based on sign (as we did) but for more than two groups, there is no straightforward automatic manner to obtain omic-specific signatures. Finally, in the two-group case, NMFProfiler also provides a slight additional flexibility since it allows a given feature to be present in the signature of several groups simultaneously (which cannot be done if signatures are built from sign based splits).

As other NMF-based methods, NMFProfiler only requires that data are nonnegative. This limitation has been leveraged in past works on omics data (Kim and Tidor 2003, Zhang *et al.* 2011) by splitting the data into positive and negative components (using the absolute value of the negative component as additional features). However, the use of the square-loss in the objective function might be subjected to limitations inherent to this specific loss and not be well adapted to highly skewed data or data containing outliers. Standard strategies (log-transformation or outlier detection and removal) can address this limitation. An alternative specific to NMF method is to replace the square-loss by the Kullback-Leibler (KL) divergence, which also has well-established optimization strategies based on specific surrogates (Fernsel and Maass 2018). While rarely tested for omics data, KL divergence has shown superior performance for mass spectrometry imaging data, which are distributed as Poisson (Nijs *et al.* 2021). Sequencing data have similar distribution and could thus also benefit from using this loss.

In the results presented in the current article, hyperparameters, and especially λ that controls the level of sparsity of the method, are automatically set based on data basic characteristics. However, additional simulations probing the influence of this parameter seem to indicate that the results can be sensitive to the value of this hyperparameter specifically. We noticed a similar situation when using NMFProfiler-prox on TCGA data. Future work could allow the automatic tuning of λ with a stability score (Liu *et al.* 2010, Meinshausen and Bühlmann 2010). However, these approaches (or cross-validation strategy) would strongly increase the computational time of the method. The current default choice implemented in our package seems to provide a satisfactory tradeoff between performance quality and computational time in various cases.

Similarly, γ, which controls the tradeoff between reconstruction quality and prediction quality, was also set to a default and basic data driven value (as a rule-of-thumb, an appropriate γ generally corresponds to a balanced contribution between reconstruction and prediction errors). Again, if the obtained results are already quite satisfactory, there might be room for improvements for this hyperparameter. In particular, based on previous remark, an adaptive strategy that would allow the update of this parameter during the optimisation from observed reconstruction and prediction errors could be an interesting idea to explore.

Finally, NMFProfiler is currently restricted to extract one signature per group and having more than one signature for a given group might require additional efforts. In some cases, this might be an interesting venue to pursue in order to identify, *e.g.* different functional pathways in various signatures. Another interesting development would be to allow for the current method to incorporate prior knowledge (*e.g.* forcing a known biomarker to be included in a signature). Very few works have addressed similar issues in the NMF litterature so far: Tang *et al.* (2012) have developped a variant of NMF where an entire signature (or a few entire signatures) is known and passed in the method to force the decomposition. Liu *et al.* (2012) have introduced a hard constrained NMF to force identical weights in clusters of individuals. The latter approach is based on a Lagrangian reformulation of the objective function and could be a course of action to incorporate various forms of prior knowledge in a flexible way.

## Supplementary Material

btaf066_Supplementary_Data

## Data Availability

The cancer TCGA data were dowloaded from http://acgt.cs.tau.ac.il/multi_omic_benchmark/download.html. The AD study data underlying this article are deposited on Recherche Data Gouv database (https://doi.org/10.57745/GWK1UW).
