# Supplementary material for: NMFProfiler: a multi-omics integration method for samples stratified in groups
Source: Bioinformatics. 2025 Feb 8;41(2):btaf066. doi: 10.1093/bioinformatics/btaf066 (PMC11855281; doi:10.1093/bioinformatics/btaf066)
Supplement: btaf066_Supplementary_Data [file btaf066_supplementary_data.zip › intsupnmf_article_suppmat.pdf]

Supplementary material of the article “NMFProfiler: A multi-omics integration method for samples stratified in groups”

A. Mercadié *et al.*

January 8, 2025

Contents

S1 Discussion on the choice of the supervised term in NMFProfiler 1

S2 Proximal optimization approach for NMFProfiler 3
S2.1 Overview . . . 3
S2.1.1 Majorize-Minimization approach . . . 4
S2.1.2 Proximal approach . . . 6
S2.1.3 Objective functions development and marginals . . . 7
S2.2 Derivation of update steps for $\mathbf{W}$, $\mathbf{H}^{(j)}$, and $\beta^{(j)}$ . . . 8
S2.2.1 Update for $\mathbf{W}$ (multiplicative update based on a surrogate) . . . 8
S2.2.2 Updates for $\mathbf{H}^{(j)}$ . . . 8
S2.2.3 Update for $\beta^{(j)}$ . . . 9
S2.2.4 Backtracking line search . . . 9

S3 Information on datasets 9
S3.1 Simulated data . . . 9
S3.2 Colon adenocarcinoma study (TCGA) . . . 10
S3.3 AD study . . . 11

S4 Information on implementation of tested methods 12

S5 Supplementary results 12
S5.1 Additional results for the main simulated dataset (Simulated dataset 02) . . . 12
S5.1.1 Simulated data . . . 12
S5.1.2 Assessment of feature selection quality . . . 13
S5.1.3 Assessment of exact sparsity quality . . . 14
S5.1.4 Assessment of sample classification quality . . . 15
S5.1.5 Computational time . . . 16
S5.2 Additional results obtained on simulated data (other frameworks) . . . 18
S5.2.1 Feature selection: numeric performance (AUROCs, 50 simulations) . . . 18
S5.2.2 Feature selection: ROC curves (50 simulations) . . . 20
S5.3 Additional methods and results for TCGA-COAD . . . 29
S5.3.1 Specific choices in the implementation of NMFProfiler and DIABLO . . . 29
S5.3.2 Cox model to explain survival . . . 30
S5.4 Additional methods and results for AD study . . . 34

S6 List of figures, tables and references 37

S1 Discussion on the choice of the supervised term in NMFProfiler

In the following, we will denote by $\mathbf{P}$ a $n \times p$ matrix of data corresponding to two groups (or classes) of individuals encoded in a vector $\mathbf{y} \in \{0, 1\}$. (Fisher) Linear Discriminant Analysis (LDA) finds the projection of $\mathbf{P}$ onto a vector of $\mathbb{R}^p$, $\boldsymbol{\beta}_F$, that is given by

$$\boldsymbol{\beta}_F = S^+(g_1 - g_2)$$

where S^+ is the pseudo-inverse of the variance of $\mathbf{P}$ and g_j is the center of gravity of group j. g_j is also, up to a scaling factor, the solution of the linear regression problem (least squares) $\|\mathbf{y}-\mathbf{P}^\top\boldsymbol{\beta}_F\|^2$, provided that $\mathbf{y}$ and $\mathbf{P}$ have previously been centered [Duda et al., 2000].

The supervised setting of the FR-lda NMF [Fernsel and Maass, 2018] is based on the addition of the LDA least-square loss into the NMF loss function. However, in the nonnegative case, the least-square solution of $\|\mathbf{y}-\mathbf{P}^\top\boldsymbol{\beta}_{\text{FR-lda}}\|^2$ is constrained to be positive (and data are thus not centered). It is thus equal to:

$$\boldsymbol{\beta}_{\text{FR-lda}} = \left(\mathbf{P}^\top\mathbf{P}\right)^+ \mathbf{P}^\top\mathbf{y} = \left(\mathbf{P}^\top\mathbf{P}\right)^+ g_1.$$

Let us illustrate the difference on an ideal case derived from the NMF background described in the main document, where:

- $K = 2$ and $n_1 = n_2 = n/2$;
- $\mathbf{P}$ is the projection of $\mathbf{X}$ onto a 2-dimensional signature matrix $\mathbf{H}$;
- each dimension of the projection is perfectly accurate for the groups: $\mathbf{P}_{i.} \simeq (1, 0)$ for i in the first group and $\mathbf{P}_{i.} \simeq (0, 1)$ for i in the second group.

In this case, $\boldsymbol{\beta}_{\text{NMF}}$ is roughly equal to g_1 (up to a scaling factor), which is far from being the LDA projection, as shown in Figure S1.

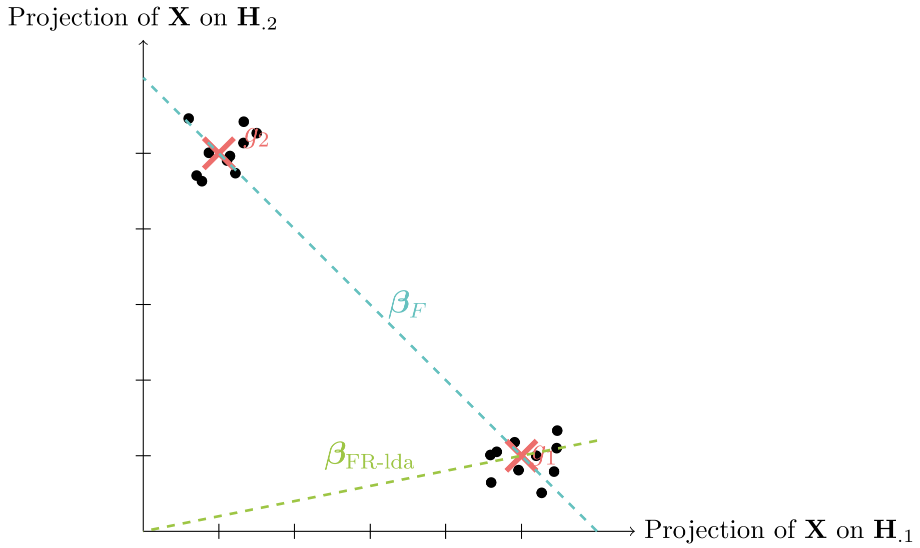

Figure S1: Solutions of the least-square problems in the case of the standard LDA ($\boldsymbol{\beta}_F$) and of the nonnegative constrained LDA ($\boldsymbol{\beta}_{\text{FR-lda}}$) as used in FR-lda.

Our proposal is to modify the original criterion to express it as two (or K for K groups) independent one-dimensional regression problems because $\left\|\mathbf{Y}-\mathbf{P}^\top\text{Diag}\left(\boldsymbol{\beta}\right)\right\|_F^2$ is equivalent to fitting independently the two linear regressions

$$\mathbf{Y}_{.1} \sim \boldsymbol{\beta}_1\mathbf{P}_{.1} \qquad \text{and} \qquad \mathbf{Y}_{.2} \sim \boldsymbol{\beta}_2\mathbf{P}_{.2}.$$

Intuitively, this approach is illustrated in Figure S2: The coordinates of the $(\mathbf{P}_{i.})_i$ on the first axis shrank by $\boldsymbol{\beta}_1$ so as to make their values close to 1 for the individuals of group 1 and close to 0 for the individuals of group 2. A similar shrinkage is performed by $\boldsymbol{\beta}_2$ in the opposite direction in terms of groups.

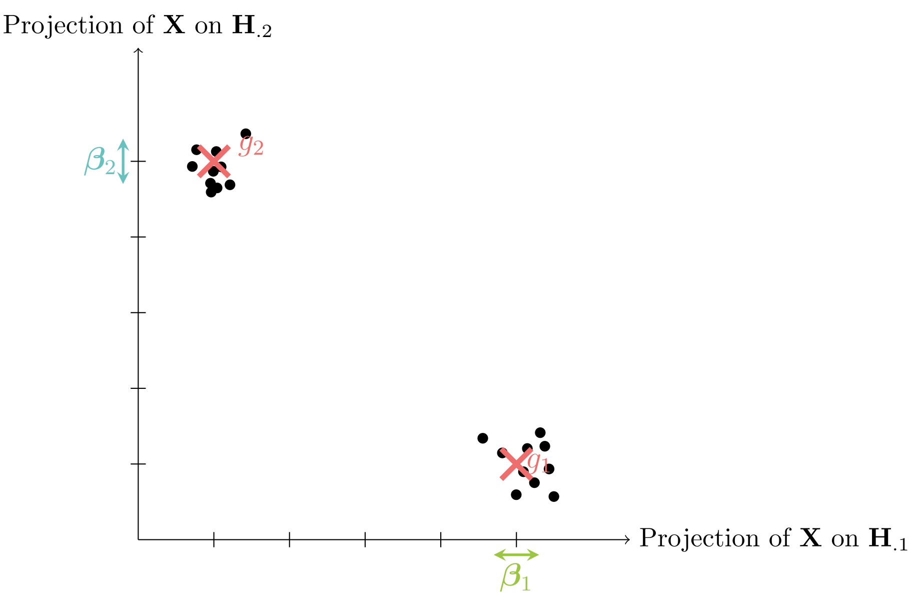

Figure S2: Illustration of the supervised part LDA of NMFProfiler.

S2 Proximal optimization approach for NMFProfiler

The notations used in this section are the same than the one introduced in the article. We describe the new proximal optimization developed for NMFProfiler in the general setting of K groups (thus K signatures extracted). We thus aim to minimize $\min_{\mathbf{W},\mathbf{H}^{(1)},\dots,\mathbf{H}^{(J)},\boldsymbol{\beta}^{(1)},\dots,\boldsymbol{\beta}^{(J)}} \mathcal{F}\left(\mathbf{W}, \{\mathbf{H}^{(j)}\}_{j=1}^J, \{\boldsymbol{\beta}^{(j)}\}_{j=1}^J\right)$, where

$$\mathcal{F}\left(\mathbf{W}, \{\mathbf{H}^{(j)}\}_{j=1}^J, \{\boldsymbol{\beta}^{(j)}\}_{j=1}^J\right) = \frac{1}{2}\left(\sum_{j=1}^J \left\|\mathbf{X}^{(j)} - \mathbf{W}\mathbf{H}^{(j)}\right\|_F^2\right) + \frac{\gamma}{2}\left(\sum_{j=1}^J \left\|\mathbf{Y} - \mathbf{X}^{(j)}\mathbf{H}^{(j)\top}\mathrm{Diag}\left(\boldsymbol{\beta}^{(j)}\right)\right\|_F^2\right) + \lambda \sum_{j=1}^J \left\|\mathbf{H}^{(j)}\right\|_1 + \frac{\mu}{2}\|\mathbf{W}\|_F^2 \tag{1}$$

with:

- $\mathbf{W} \in \mathbb{R}_+^{n\times K}$ the common contributions of the individuals to latent factors,
- $\forall j \in \{1,\dots,J\}$, $\mathbf{H}^{(j)} \in \mathbb{R}_+^{K\times p_j}$ the latent components,
- $\forall j \in \{1,\dots,J\}, \mathrm{Diag}\left(\boldsymbol{\beta}^{(j)}\right)$, the $K \times K$ diagonal matrix with diagonal entries equal to $\boldsymbol{\beta}_k^{(j)}$ where k represents a given "group" in $\{1,\dots,K\}$,
- λ, γ, ν, $\mu > 0$ are regularization parameters (given).

S2.1 Overview

The optimization problems of NMF are described as "ill-posed, non-linear and non-convex" [Fernsel and Maass, 2018]. Indeed, the functions $\mathcal{F}_l$ are not simultaneously convex in $\mathbf{W}$, $\mathbf{H}^{(j)}$, and $\boldsymbol{\beta}^{(j)}$, but can be written as separated convex optimization problems in each variable, one of these problems including non-smooth constraints. A way to solve this type of problems is to use alternating algorithms, where each matrix is iteratively updated, as described in Algorithm 1.

In this algorithm, the updates for $\mathbf{W}$, $\mathbf{H}^{(j)}$, and $\boldsymbol{\beta}^{(j)}$ are all using a surrogate approach, where the objective function for the corresponding variable is bounded by a surrogate function. However, these surrogates are obtained with two different principles:

- updates for $\mathbf{W}$ and $\boldsymbol{\beta}^{(j)}$ are obtained with a Majorize-Minimization (MM) approach that is equivalent to Multiplicative Updates (MU), automatically yielding positivity of the matrices;
- contrary to what is described in [Fernsel and Maass, 2018], $\mathbf{H}^{(j)}$ updates are based on a proximal approach, which ensures exact sparsity of the matrices (which is not the case of the previous approach that requires strictly positive updates).

These two different principles are respectively described in turn below.

Algorithm 1 Overview of the algorithm solving Equation (4)

1: Initialize matrices $\mathbf{W}^{(0)}$, $\mathbf{H}^{(j,0)}$, vectors $\boldsymbol{\beta}^{(j,0)}$ with strictly positive values.
2: **for all** $t = 1, \ldots, T$ **do**
3: MU update: $\mathbf{W}^{(t+1)} \leftarrow \mathbf{W}^{(t)} \odot \mathbf{A}(\mathbf{W}^{(t)})$
4: MU or Prox update: $\forall\, j = 1, \ldots, J$, (Prox)

$$\mathbf{H}^{(j,t+1)} \leftarrow \text{prox}_{\tilde{g}_j}\left(\tilde{\mathbf{H}}^{(j)}\right),\ \tilde{\mathbf{H}}^{(j)} = \mathbf{H}^{(j,t)} - \frac{1}{\eta}\nabla f_j(\mathbf{H}^{(j,t)})$$

OR (MU):

$$\mathbf{H}^{(j,t+1)} \leftarrow \mathbf{H}^{(j,t)} \odot \mathbf{S}(\mathbf{H}^{(j,t)})$$

5: OLS solution: $\forall\, j = 1, \ldots, J$, $\forall\, k = 1, \ldots, K$,

$$\boldsymbol{\beta}_k^{(j,t+1)} \leftarrow \frac{\mathbf{H}_{k.}^{(j,t+1)}\mathbf{X}^{(j)\top}\mathbf{Y}_{.k}}{\mathbf{H}_{k.}^{(j,t+1)}\mathbf{X}^{(j)\top}\mathbf{X}^{(j)}\mathbf{H}_{k.}^{(j,t+1)\top}}$$

6: **end for**
7: **return** $\mathbf{W} := \mathbf{W}^{(T+1)}$, $\mathbf{H}^{(j)} := \mathbf{H}^{(j,T+1)}$ and $\boldsymbol{\beta}^{(j)} := \boldsymbol{\beta}^{(j,T+1)}$ $(j = 1, \ldots, J)$

$\odot$ is the element-wise multiplication and precise values of $\mathbf{A}(.)$, $f_j(.)$, $\tilde{g}_j(.)$, and $\mathbf{S}(.)$ are given in Sections S2.2.1 and S2.2.2.

S2.1.1 Majorize-Minimization approach

This section describes the basic principle of the MM approach for a general optimization problem

$$\min_{\mathbf{a} \in \mathbb{R}^D} \mathcal{F}(\mathbf{a}).$$

In the specific case of the NMF, this principle leads to MU for $\mathbf{a}$ (either $\mathbf{W}$ or $\boldsymbol{\beta}^{(j)}$) that ensures positivity, as shown later.

The MM principle does not require that the objective function $\mathcal{F}$ is convex or differentiable. Instead, it defines a surrogate $\mathcal{Q}_{\mathcal{F}}(\cdot,\cdot)$, which is a function $\mathbb{R}^D \times \mathbb{R}^D \to \mathbb{R}$ that is easy to minimize in its first component and that satisfies:

- $\mathcal{Q}_{\mathcal{F}}(\mathbf{a}, \mathbf{a}) = \mathcal{F}(\mathbf{a})$, $\forall \mathbf{a} \in \mathbb{R}^D$,
- $\mathcal{F}(\mathbf{a}) \leq \mathcal{Q}_{\mathcal{F}}(\mathbf{a}, \mathbf{b})$, $\forall \mathbf{a}, \mathbf{b} \in \mathbb{R}^D$.

Since, $\mathcal{Q}_{\mathcal{F}}(\mathbf{a}, \mathbf{a}^{(t)})$ is an upper-bound of $\mathcal{F}(\mathbf{a})$ for all $\mathbf{a} \in \mathbb{R}^D$, setting an update of $\mathbf{a}$ of the form

$$\mathbf{a}^{(t+1)} = \text{argmin}_{\mathbf{a}}\, \mathcal{Q}_{\mathcal{F}}(\mathbf{a}, \mathbf{a}^{(t)}) \tag{2}$$

leads to

$$\mathcal{F}(\mathbf{a}^{(t+1)}) \leq \mathcal{Q}_{\mathcal{F}}(\mathbf{a}^{(t+1)}, \mathbf{a}^{(t)}) \leq \mathcal{Q}_{\mathcal{F}}(\mathbf{a}^{(t)}, \mathbf{a}^{(t)}) = \mathcal{F}(\mathbf{a}^{(t)})$$

and thus ensures the decrease of $\mathcal{F}(\mathbf{a}^{(t)})$ at each iteration of the algorithm.

Below is described a common approach to define a proper surrogate corresponding to methods used to define the surrogate of $\mathbf{W}$.

Upper Quadratic Bound Principle The Upper Quadratic Bound Principle (UQBP) principle requires that $\mathcal{F}$ is twice differentiable and is based on the majorization of the quadratic term in the second order Taylor development of the objective function of interest:

$$\mathcal{F}(\mathbf{a}) = \mathcal{F}(\mathbf{b}) + (\mathbf{a} - \mathbf{b})^\top \nabla \mathcal{F}(\mathbf{b}) + \frac{1}{2}(\mathbf{a} - \mathbf{b})^\top \triangle^2 \mathcal{F}(\mathbf{b})(\mathbf{a} - \mathbf{b}) + o(\|\mathbf{a} - \mathbf{b}\|^2)$$

$\mathcal{Q}_{\mathcal{F}}$ is then chosen as

$$\mathcal{Q}_{\mathcal{F}}(\mathbf{a}, \mathbf{b}) = \mathcal{F}(\mathbf{b}) + (\mathbf{a} - \mathbf{b})^\top \nabla \mathcal{F}(\mathbf{b}) + \frac{1}{2}(\mathbf{a} - \mathbf{b})^\top \mathbf{M}(\mathbf{b})(\mathbf{a} - \mathbf{b})$$

with $\mathbf{M}(\mathbf{b})$ chosen so that $\mathbf{M}(\mathbf{b}) - \triangle^2 \mathcal{F}(\mathbf{b})$ is positive semi-definite.

Proposition 1. *Let $\mathcal{F} : \mathbb{R}^D \to \mathbb{R}$ be a twice continuously differentiable function and $\mathbf{M}(\mathbf{b}) \in \mathbb{R}^{D \times D}$ a matrix such that, $\forall \mathbf{b} \in \mathbb{R}^D$, $\mathbf{M}(\mathbf{b}) - \triangle^2 \mathcal{F}(\mathbf{b})$ is semi-definite positive (with $\triangle^2 \mathcal{F}(\mathbf{b})$ the Hessian of $\mathcal{F}$ at $\mathbf{b}$). Then, when $\mathcal{F}$ is a second degree polynomial, $\mathcal{Q}_{\mathcal{F}}$ is a surrogate of $\mathcal{F}$. In addition, when $\mathbf{M}(\mathbf{b})$ is symmetric and positive definite, the corresponding surrogate is strictly convex and its minimization leads to the following update rule:*

$$\mathbf{a}^{(t+1)} = \mathbf{a}^{(t)} - \mathbf{M}^{-1}(\mathbf{a}^{(t)}) \nabla \mathcal{F}(\mathbf{a}^{(t)}).$$

Proof. First, let us show that $\mathcal{Q}_{\mathcal{F}}$ is a surrogate for $\mathcal{F}$. We have:

- $\forall \mathbf{a} \in \mathbb{R}^D$, $\mathcal{Q}_{\mathcal{F}}(\mathbf{a}, \mathbf{a}) = \mathcal{F}(\mathbf{a}) + (\mathbf{a} - \mathbf{a})^\top \nabla \mathcal{F}(\mathbf{a}) + \frac{1}{2}(\mathbf{a} - \mathbf{a})^\top \mathbf{M}(\mathbf{a})(\mathbf{a} - \mathbf{a}) = \mathcal{F}(\mathbf{a})$
- since $\mathbf{M}(\mathbf{b}) - \triangle^2 \mathcal{F}(\mathbf{b})$ is semi-definite positive $\forall \mathbf{b} \in \mathbb{R}^D$, we have $\forall \mathbf{a}$, $\mathbf{a}^{(t)} \in \mathbb{R}^D$, $(\mathbf{a} - \mathbf{a}^{(t)})^\top \triangle^2 \mathcal{F}(\mathbf{a}^{(t)})(\mathbf{a} - \mathbf{a}^{(t)}) \leq (\mathbf{a} - \mathbf{a}^{(t)})^\top \mathbf{M}(\mathbf{a}^{(t)})(\mathbf{a} - \mathbf{a}^{(t)})$. In addition, since $\mathcal{F}$ is a second degree polynomial, it is equal to its second degree Taylor expansion and thus:

  $$\mathcal{F}(\mathbf{a}) = \mathcal{F}(\mathbf{b}) + (\mathbf{a} - \mathbf{b})^\top \nabla \mathcal{F}(\mathbf{b}) + \frac{1}{2}(\mathbf{a} - \mathbf{b})^\top \triangle^2 \mathcal{F}(\mathbf{b})(\mathbf{a} - \mathbf{b}) \leq \mathcal{Q}_{\mathcal{F}}(\mathbf{a}, \mathbf{b}),$$

  which concludes the proof that $\mathcal{Q}_{\mathcal{F}}$ is a surrogate for $\mathcal{F}$.

Then, let us show that $\mathcal{Q}_{\mathcal{F}}$ is strictly convex whenever $\mathbf{M}(\mathbf{b})$ is symmetric and positive definite. Thanks to the symmetry of $\mathbf{M}(\mathbf{b})$, we get:

$$\nabla_{\mathbf{a}} \mathcal{Q}_{\mathcal{F}}(\mathbf{a}, \mathbf{b}) = \nabla \mathcal{F}(\mathbf{b}) + \mathbf{M}(\mathbf{b})(\mathbf{a} - \mathbf{b})$$

and

$$\triangle^2_{\mathbf{a}} \mathcal{Q}_{\mathcal{F}}(\mathbf{a}, \mathbf{b}) = \mathbf{M}(\mathbf{b})$$

Thus, as $\mathbf{M}(\mathbf{b})$ is positive definite, we can conclude that $\mathcal{Q}_{\mathcal{F}}$ is a strictly convex function having a unique minimizer.

In addition, as stated by Equation (2), the update rule is thus given by the minimization of $\mathcal{Q}_{\mathcal{F}}(\mathbf{a}, \mathbf{a}^{(t)})$ over $\mathbf{a} \in \mathbb{R}^D$. Using the first order optimality condition, we have:

$$\begin{aligned} \nabla \mathcal{Q}_{\mathcal{F}}(\mathbf{a}^{(t+1)}, \mathbf{a}^{(t)}) = 0 \quad &\Leftrightarrow \quad \nabla \mathcal{F}(\mathbf{a}^{(t)}) + \mathbf{M}(\mathbf{a}^{(t)})(\mathbf{a}^{(t+1)} - \mathbf{a}^{(t)}) = 0 \\ &\Leftrightarrow \quad \mathbf{a}^{(t+1)} = \mathbf{a}^{(t)} - \mathbf{M}(\mathbf{a}^{(t)})^{-1} \nabla \mathcal{F}(\mathbf{a}^{(t)}) \end{aligned}$$

□

[Fernsel and Maass, 2018] suggest to choose $\mathbf{M}(\mathbf{b})$ as

$$\mathbf{M}(\mathbf{b}) = \left[\delta_{i,j} \frac{\left(\triangle^2 \mathcal{F}(\mathbf{b}) \mathbf{b}\right)_i}{\mathbf{b}_i} \right]_{i,j=1,\ldots,D}. \tag{3}$$

The following proposition shows that this choice satisfies the conditions of Proposition 1.

Proposition 2. $\mathbf{M}(\mathbf{b})$*, as defined in Equation (3), is positive definite and* $\mathbf{M}(\mathbf{b}) - \triangle^2 \mathcal{F}(\mathbf{b})$ *is positive semi-definite.*

Proof. The following more general lemma is sufficient to prove the proposition:

Lemme 1. *Let* $\mathbf{U} \in \mathbb{R}_+^{D \times D}$ *be a symmetric matrix and* $\mathbf{b} \in \mathbb{R}_+^D$ *a vector. Let* $\mathbf{M}(\mathbf{b})$ *be defined as:*

$$\mathbf{M}(\mathbf{b}) = \left[\delta_{i,j} \frac{(\mathbf{U}\mathbf{b})_i}{\mathbf{b}_i} \right]_{i,j=1,\ldots,D}$$

Then $\mathbf{M}(\mathbf{b})$ *is positive definite and* $\mathbf{M}(\mathbf{b}) - \mathbf{U}$ *is positive semi-definite.*

First, let us show that $\mathbf{M}(\mathbf{b}) - \mathbf{U}$ is positive semi-definite. Let $\mathbf{v} \in \mathbb{R}_*^D$ (*i.e.* $\mathbf{v} \neq 0$) an arbitrary vector. Then:

$$\begin{aligned}
\mathbf{v}^\top (\mathbf{M}(\mathbf{b}) - \mathbf{U})\, \mathbf{v} &= \sum_{i=1}^{D}\sum_{j=1}^{D} \mathbf{v}_i \left(\mathbf{M}(\mathbf{b}) - \mathbf{U}\right)_{ij} \mathbf{v}_j \\
&= \sum_{i=1}^{D}\sum_{j=1}^{D} \mathbf{v}_i \delta_{ij} \frac{(\mathbf{U}\mathbf{b})_i}{\mathbf{b}_i} \mathbf{v}_j - \sum_{i=1}^{D}\sum_{j=1}^{D} \mathbf{v}_i \mathbf{U}_{ij} \mathbf{v}_j \\
&= \sum_{i=1}^{D} \mathbf{v}_i^2 \frac{(\mathbf{U}\mathbf{b})_i}{\mathbf{b}_i} - \sum_{i=1}^{D}\sum_{j=1}^{D} \mathbf{v}_i \mathbf{U}_{ij} \mathbf{v}_j \\
&= \sum_{i=1}^{D}\sum_{j=1}^{D} \mathbf{v}_i^2 \mathbf{U}_{ij} \frac{\mathbf{b}_j}{\mathbf{b}_i} - \sum_{i=1}^{D}\sum_{j=1}^{D} \mathbf{v}_i \mathbf{U}_{ij} \mathbf{v}_j \\
&= \sum_{i=1}^{D} \mathbf{v}_i^2 \mathbf{U}_{ii} + \sum_{i=1}^{D}\sum_{\substack{j=1 \\ j\neq i}}^{D} \mathbf{v}_i^2 \mathbf{U}_{ij} \frac{\mathbf{b}_j}{\mathbf{b}_i} - \sum_{i=1}^{D}\sum_{j=1}^{D} \mathbf{v}_i \mathbf{U}_{ij} \mathbf{v}_j \\
&= \sum_{i=1}^{D} \mathbf{v}_i^2 \mathbf{U}_{ii} + \sum_{\substack{i,j=1 \\ i<j}}^{D} \left(\mathbf{v}_i^2 \frac{\mathbf{b}_j}{\mathbf{b}_i} + \mathbf{v}_j^2 \frac{\mathbf{b}_i}{\mathbf{b}_j} \right) \mathbf{U}_{ij} - \sum_{i=1}^{D}\sum_{j=1}^{D} \mathbf{v}_i \mathbf{U}_{ij} \mathbf{v}_j \\
&= \sum_{i=1}^{D}\sum_{j=1}^{D} \left[\frac{1}{2} \mathbf{v}_i^2 \frac{\mathbf{b}_j}{\mathbf{b}_i} \mathbf{U}_{ij} + \frac{1}{2} \mathbf{v}_j^2 \frac{\mathbf{b}_i}{\mathbf{b}_j} \mathbf{U}_{ij} - \mathbf{v}_i \mathbf{U}_{ij} \mathbf{v}_j \right] \\
&= \frac{1}{2} \sum_{i=1}^{D}\sum_{j=1}^{D} \left[\mathbf{v}_i^2 \frac{\mathbf{b}_j}{\mathbf{b}_i} \mathbf{U}_{ij} + \mathbf{v}_j^2 \frac{\mathbf{b}_i}{\mathbf{b}_j} \mathbf{U}_{ij} - 2\sqrt{\frac{\mathbf{b}_j}{\mathbf{b}_i}} \sqrt{\frac{\mathbf{b}_i}{\mathbf{b}_j}} \mathbf{v}_i \mathbf{U}_{ij} \mathbf{v}_j \right] \\
&= \frac{1}{2} \sum_{i=1}^{D}\sum_{j=1}^{D} \left[\mathbf{v}_i^2 \frac{\mathbf{b}_j}{\mathbf{b}_i} + \mathbf{v}_j^2 \frac{\mathbf{b}_i}{\mathbf{b}_j} - 2\sqrt{\frac{\mathbf{b}_j}{\mathbf{b}_i}} \sqrt{\frac{\mathbf{b}_i}{\mathbf{b}_j}} \mathbf{v}_i \mathbf{v}_j \right] \mathbf{U}_{ij} \\
&= \frac{1}{2} \sum_{i=1}^{D}\sum_{j=1}^{D} \left(\mathbf{v}_i \sqrt{\frac{\mathbf{b}_j}{\mathbf{b}_i}} - \mathbf{v}_j \sqrt{\frac{\mathbf{b}_i}{\mathbf{b}_j}} \right)^2 \mathbf{U}_{ij} \geq 0
\end{aligned}$$

As $\forall \mathbf{b} \in \mathbb{R}_+^2$, $\mathbf{M}(\mathbf{b})$ is a diagonal matrix, it is also symmetric. And we have, with $\mathbf{v} \in \mathbb{R}_*^D$ (*i.e.* $\mathbf{v} \neq 0$) an arbitrary vector:

$$\mathbf{v}^\top \mathbf{M}(\mathbf{b}) \mathbf{v} = \sum_{i=1}^{D}\sum_{j=1}^{D} \mathbf{v}_i \frac{(\mathbf{U}\mathbf{b})_i}{\mathbf{b}_i} \mathbf{v}_j = \sum_{i=1}^{D} \mathbf{v}_i^2 \sum_{j=1}^{D} \mathbf{U}_{ij} \frac{\mathbf{b}_j}{\mathbf{b}_i} > 0.$$

Thus, $\forall \mathbf{b} \in \mathbb{R}_+^2$, $\mathbf{M}(\mathbf{b})$ is also a positive definite matrix. □

This principle is used to update $\mathbf{W}$ as described in Section S2.2.1. In addition, taking $\mathbf{M}(\mathbf{b})$ as in Equation (3) ensures that the update for $\mathbf{a}^{(t)}$ is multiplicative and that its entries remain positive.

S2.1.2 Proximal approach

The minimization problem in $\mathbf{H}$ includes an ℓ_1 penalty term, which is not differentiable. However, as shown in [Leuschner et al., 2019], the UQBP principle could still be used in this case and still yields a multiplicative update. However, it would not lead to an exact sparsity (in short, the authors need to add a small offset to zero components in $\mathbf{H}$ to perform the multiplicative updates). Hence, we propose a more direct approach that ensures sparsity for the update of $\mathbf{H}$. This purpose's section is thus to explain the general principle of this optimization, using a proximal operator.

This section describes a general method to solve optimization problems

$$\min_{\mathbf{a} \in \mathbb{R}_+^D} \mathcal{F}(\mathbf{a}),$$

where $\mathcal{F}(\mathbf{a})$ takes the form $f(\mathbf{a}) + \lambda g(\mathbf{a})$ with $\lambda > 0$, f a gradient Lipschitz function (with Lipschitz contant η) and g a non smooth penalty.

In this case, the Forward Backward Splitting (FBS) algorithm [Parikh and Boyd, 2014, Bauschke and Combettes, 2017] defines the surrogate as

$$\mathcal{Q}_{\mathcal{F}}(\mathbf{a}, \mathbf{b}) = f(\mathbf{b}) + (\mathbf{a} - \mathbf{b})^\top \nabla f(\mathbf{b}) + \frac{\eta}{2} \|\mathbf{a} - \mathbf{b}\|^2 + \lambda g(\mathbf{a}).$$

Proposition 3. *Let $\mathcal{F}$ be an objective function equal to $f + \lambda g$ with $f : \mathbb{R}^D \to \mathbb{R}$ a convex and gradient Lipschitz function with a Lispchitz constant η, g a non smooth penalty, and $\lambda > 0$. Then, $\mathcal{Q}_{\mathcal{F}}$ is a surrogate of $\mathcal{F}$.*

Proof. First, let us show that $\mathcal{Q}_{\mathcal{F}}$ is a surrogate for $\mathcal{F}$. We have:

- $\forall \mathbf{a} \in \mathbb{R}^D$, $\mathcal{Q}_{\mathcal{F}}(\mathbf{a}, \mathbf{a}) = f(\mathbf{a}) + (\mathbf{a} - \mathbf{a})^\top \nabla f(\mathbf{a}) + \frac{\eta}{2}(\mathbf{a} - \mathbf{a})^\top(\mathbf{a})(\mathbf{a} - \mathbf{a}) + \lambda g(\mathbf{a}) = f(\mathbf{a}) + \lambda g(\mathbf{a}) = \mathcal{F}(\mathbf{a})$
- Since f is convex and gradient Lipschitz, we get $\forall \mathbf{a}, \mathbf{b} \in \mathbb{R}^D$,

$$\mathcal{F}(\mathbf{a}) \leq f(\mathbf{b}) + (\mathbf{a} - \mathbf{b})^\top \nabla f(\mathbf{b}) + \frac{\eta}{2}\|\mathbf{a} - \mathbf{b}\|^2 + \lambda g(\mathbf{a}) = \mathcal{Q}_{\mathcal{F}}(\mathbf{a}, \mathbf{b})$$

□

Noting that $\mathcal{Q}_{\mathcal{F}}(\mathbf{a}, \mathbf{a}^{(t)})$ has the same minimum as

$$\frac{1}{2}\|\mathbf{a} - \tilde{\mathbf{a}}\|_2^2 + \frac{\lambda}{\eta} g(\mathbf{a}),$$

with $\tilde{\mathbf{a}} = \mathbf{a}^{(t)} - \frac{1}{\eta}\nabla f(\mathbf{a}^{(t)})$, we can show that the update step based on this surrogate, *i.e.*, the solution of Equation (2), is given by:

$$\mathbf{a}^{(t+1)} = \operatorname{prox}_{\frac{\lambda}{\eta} g}(\tilde{\mathbf{a}}^t) \qquad \text{with} \qquad \tilde{\mathbf{a}}^t = \mathbf{a}^{(t)} - \frac{1}{\eta}\nabla f(\mathbf{a}^{(t)}) \tag{4}$$

where prox_g is the proximal operator of g.

In the particular case of the ℓ_1 penalty restricted to $\mathbb{R}_+^D$, g can be written as $g = \|.\|_1 + \delta_+$ with, $\forall \mathbf{a} \in \mathbb{R}^D$,

$$\delta_+(\mathbf{a}) = \begin{cases} 0 & \text{if } \mathbf{a} \geq 0 \\ +\infty & \text{otherwise.} \end{cases}$$

In this specific case, the proximal operator has an explicit form and the update step thus writes:

$$\mathbf{a}_i^{(t+1)} = \operatorname{prox}_{\frac{\lambda}{\eta}\|\cdot\|_1 + \delta_+}(\tilde{\mathbf{a}}_i) = \left[\tilde{\mathbf{a}}_i - \frac{\lambda}{\eta}\right]_+ \tag{5}$$

where $(z)_+ = \max(0, z)$ is the positive part of $z \in \mathbb{R}$.

The principle presented here is used to update $\mathbf{H}^{(j)}$ as described in Section S2.2.2.

S2.1.3 Objective functions development and marginals

As stated in the introduction of this section, the optimization is tackled using separated steps for each of the variable to optimize. The precise expression of these update steps are explained and made explicit in the next section. To ease the future calculus, this section gives the expended matrix form expression of the objective function, $\mathcal{F}$, and of its marginals in $\mathbf{W}$, $\mathbf{H}^{(j)}$, and $\boldsymbol{\beta}^{(j)}$.

Using the trace form of the Frobenius norm, we get:

$$\begin{aligned}\mathcal{F}(\mathbf{W}, \{\mathbf{H}^{(j)}\}_{j=1}^J, \{\boldsymbol{\beta}^{(j)}\}_{j=1}^J) = \frac{1}{2}\sum_{j=1}^J \Big[\operatorname{Tr}(\mathbf{X}^{(j)\top}\mathbf{X}^{(j)}) - 2\operatorname{Tr}(\mathbf{H}^{(j)\top}\mathbf{W}^\top\mathbf{X}^{(j)}) + \operatorname{Tr}(\mathbf{H}^{(j)\top}\mathbf{W}^\top\mathbf{W}\mathbf{H}^{(j)})\Big] \\ + \frac{\gamma}{2}\sum_{j=1}^J \left\|\mathbf{Y} - \mathbf{X}^{(j)}\mathbf{H}^{(j)\top}\operatorname{Diag}\left(\boldsymbol{\beta}^{(j)}\right)\right\|_F^2 + \lambda\sum_{j=1}^J \|\mathbf{H}^{(j)}\|_1 + \frac{\mu}{2}\operatorname{Tr}(\mathbf{W}^\top\mathbf{W}).\end{aligned} \tag{6}$$

Based on Equation (6), we can then derive marginals of the objective function, each of which corresponds to one of the function minimized in the different update steps of the overall optimization Algorithm 1:

$$F(\mathbf{W}) := \sum_{j=1}^J \left(\frac{1}{2}\operatorname{Tr}(\mathbf{H}^{(j)\top}\mathbf{W}^\top\mathbf{W}\mathbf{H}^{(j)}) - \operatorname{Tr}(\mathbf{H}^{(j)\top}\mathbf{W}^\top\mathbf{X}^{(j)})\right) + \frac{\mu}{2}\operatorname{Tr}(\mathbf{W}^\top\mathbf{W}), \tag{7}$$

$$\begin{aligned}\forall j \in \{1, \ldots, J\}, \qquad G_j(\mathbf{H}^{(j)}) = \frac{1}{2}\operatorname{Tr}(\mathbf{H}^{(j)\top}\mathbf{W}^\top\mathbf{W}\mathbf{H}^{(j)}) - \operatorname{Tr}(\mathbf{H}^{(j)\top}\mathbf{W}^\top\mathbf{X}^{(j)}) + \\ + \frac{\gamma}{2}\operatorname{Tr}\left(\operatorname{Diag}\left((\boldsymbol{\beta}^{(j)})^2\right)\mathbf{H}^{(j)}\mathbf{X}^{(j)\top}\mathbf{X}^{(j)}\mathbf{H}^{(j)\top}\right) - \gamma\operatorname{Tr}\left(\operatorname{Diag}(\boldsymbol{\beta}^{(j)})\mathbf{H}^{(j)}\mathbf{X}^{(j)\top}\mathbf{Y}\right) \\ + \lambda\|\mathbf{H}^{(j)}\|_1,\end{aligned} \tag{8}$$

with $\operatorname{Diag}\left((\boldsymbol{\beta}^{(j)})^2\right)$ the $K \times K$ diagonal matrix with diagonal entries equal to $\left(\beta_k^{(j)}\right)^2$,

$$\forall j \in \{1, \ldots, J\}, \qquad E_j(\boldsymbol{\beta}^{(j)}) = \frac{\gamma}{2}\left\|\mathbf{Y} - \mathbf{X}^{(j)}\mathbf{H}^{(j)\top}\operatorname{Diag}\left(\boldsymbol{\beta}^{(j)}\right)\right\|_F^2. \tag{9}$$

S2.2 Derivation of update steps for $\mathbf{W}$, $\mathbf{H}^{(j)}$, and $\beta^{(j)}$

This section builds on principles described in Section S2.1.1 to derive the update steps 4 (for $\mathbf{W}$), 5 (for $\mathbf{H}^{(j)}$) and 6 (for $\boldsymbol{\beta}^{(j)}$) of Algorithm 1, respectively.

S2.2.1 Update for $\mathbf{W}$ (multiplicative update based on a surrogate)

For the sake of simplicity, we omit the step number t for matrices that are not updated (*i.e.*, $\mathbf{H}^{(j)}$ will be used for the current value of this matrix, $\mathbf{H}^{(j,t)}$, and similarly for $\boldsymbol{\beta}^{(j,t)}$). Combining the update step defined in Proposition 1 with the value given in Equation (3), we obtain the update rule for $\mathbf{W}$ as: $\forall i = 1, \dots, n$:

$$\mathbf{w}_i^{(t+1)} = \mathbf{w}_i^{(t)} - \mathbf{M}^{-1}(\mathbf{w}_i^{(t)})\nabla F(\mathbf{w}_i^{(t)}) \qquad \text{with} \qquad \mathbf{M}\left(\mathbf{w}_i^{(t)}\right) = \left[\delta_{k,k'} \frac{\left(\triangle^2 F(\mathbf{w}_i^{(t)})\mathbf{w}_i^{(t)}\right)_k}{\mathbf{w}_{ik}^{(t)}}\right]_{k,k'=1,\dots,K}, \tag{10}$$

where $\mathbf{w}_i$ is a column vector of $\mathbb{R}^K$ whose entries correspond to the i-th row of $\mathbf{W}$. Thus, $\mathbf{w}_{ik}$ denotes the entry (i,k) of the matrix $\mathbf{W}$.

Using Equation (7), we get

$$\begin{aligned}\nabla F(\mathbf{W}) &= \sum_{j=1}^{J} \mathbf{W}\mathbf{H}^{(j)}\mathbf{H}^{(j)\top} - \sum_{j=1}^{J} \mathbf{X}^{(j)}\mathbf{H}^{(j)\top} + \mu\mathbf{W} \\ &= \mathbf{W}\left[\sum_{j=1}^{J} \mathbf{H}^{(j)}\mathbf{H}^{(j)\top} + \mu\mathbb{I}_K\right] - \left[\sum_{j=1}^{J} \mathbf{X}^{(j)}\mathbf{H}^{(j)\top}\right] \\ &= \mathbf{W}\mathbf{B} - \mathbf{C},\end{aligned}$$

where $\mathbf{B} = \mu\mathbb{I}_K + \sum_{j=1}^{J} \mathbf{H}^{(j)}\mathbf{H}^{(j)\top}$ and $\mathbf{C} = \sum_{j=1}^{J} \mathbf{X}^{(j)}\mathbf{H}^{(j)\top}$. Thus, we finally have:

$$\forall\, i = 1, \dots, n, \qquad \triangle^2 F(\mathbf{w}_i) = \mathbf{B}.$$

Using " $(./)$ " for the element-wise division, we thus have

$$\mathbf{M}\left(\mathbf{w}_i^{(t)}\right)^{-1} = \mathrm{Diag}\left(\mathbf{w}_i^{(t)}\,(./)(\mathbf{B}\mathbf{w}_i^{(t)})\right),$$

a $K \times K$-matrix, where $\mathrm{Diag}(\mathbf{z})$ is the diagonal matrix having for diagonal entry values $\mathbf{z}$.

Plugging the values of $\nabla F(\mathbf{w}_i)$ and $\mathbf{M}(\mathbf{w}_i^{(t)})^{-1}$ in Equation (10), we have

$$\mathbf{w}_i^{(t+1)} = \mathbf{w}_i^{(t)} - \mathrm{Diag}\left(\mathbf{w}_i^{(t)}\,(./)(\mathbf{B}\mathbf{w}_i^{(t)})\right) \times \left[\mathbf{W}^{(t)}\mathbf{B} - \mathbf{C}\right]_{i,\cdot}^{\top}.$$

Noting that the matrix multiplication is equivalent to element-wise multiplication with diagonal matrices and using the fact that $\mathbf{B}$ is symmetric, we finally get

$$\begin{aligned}\mathbf{w}_i^{(t+1)} &= \mathbf{w}_i^{(t)} - \left(\mathbf{w}_i^{(t)}\,(./)(\mathbf{B}\mathbf{w}_i^{(t)}) \odot (\mathbf{B}\mathbf{w}_i^{(t))}\right) + \left(\mathbf{w}_i^{(t)}\,(./)(\mathbf{B}\mathbf{w}_i^{(t)}) \odot \mathbf{C}_{i,\cdot}^{\top}\right) \\ &= \mathbf{w}_i^{(t)} - \mathbf{w}_i^{(t)} + \mathbf{w}_i^{(t)} \odot \left(\mathbf{C}_{i,\cdot}^{\top}\,(./)(\mathbf{B}\mathbf{w}_i^{(t)})\right) \\ &= \mathbf{w}_i^{(t)} \odot \left(\mathbf{C}_{i,\cdot}^{\top}\,(./)(\mathbf{B}\mathbf{w}_i^{(t)})\right)\end{aligned}$$

Finally setting $\mathbf{A}\left(\mathbf{W}^{(t)}\right) := \mathbf{C}\,(./)(\mathbf{W}^{(t)}\mathbf{B})$, we obtain the update step for $\mathbf{W}$ given in Algorithm 1.

S2.2.2 Updates for $\mathbf{H}^{(j)}$

For the sake of simplicity, we omit the step number t for matrices that are not updated (*i.e.*, $\mathbf{W}$ will be used for the current value of this matrix, $\mathbf{W}^{(t)}$, and similarly for $\boldsymbol{\beta}^{(j,t)}$). For the same reason, since the objective function for $\mathbf{H}^{(j)}$ is separable in j, indices j (related to the omics datasets) are omitted too (*i.e.*, $\mathbf{H}$ will refer to $\mathbf{H}^{(j)}$, $\mathbf{X}$ to $\mathbf{X}^{(j)}$, $\boldsymbol{\beta}$ to $\boldsymbol{\beta}^{(j)}$, G to G_j, f to f_j, g to g_j and p to p_j).

Multiplicative update based on a surrogate The objective function wrt $\mathbf{H}$ is

$$G(\mathbf{H}) = \frac{1}{2}\mathrm{Tr}(\mathbf{H}^\top\mathbf{W}^\top\mathbf{W}\mathbf{H}) - \mathrm{Tr}(\mathbf{H}^\top\mathbf{W}^\top\mathbf{X}) + \frac{\gamma}{2}\|\mathbf{Y} - \mathbf{X}\mathbf{H}^\top\mathrm{Diag}\,(\boldsymbol{\beta})\,\|_F^2 + \lambda\|\mathbf{H}\|_1.$$

Solved similarly as in Section S2.2.1, we end up with the following update rule:

$$\mathbf{H}^{(t+1)} = \mathbf{H}^{(t)} \odot \mathbf{S}\left(\mathbf{H}^{(t)}\right)$$

where

$$\mathbf{S}\left(\mathbf{H}^{(t)}\right) := (\mathbf{W}^\top\mathbf{X} + \gamma\mathrm{Diag}(\boldsymbol{\beta})\mathbf{Y}^\top\mathbf{X})\,(./)(\mathbf{W}^\top\mathbf{W}\mathbf{H}^{(t)} + \gamma\mathrm{Diag}(\boldsymbol{\beta}^2)\mathbf{H}^{(t)}\mathbf{X}^\top\mathbf{X}).$$

Proximal approach $G(\mathbf{H})$ can also be partitionned as

$$G(\mathbf{H}) = \underbrace{\frac{1}{2}\operatorname{Tr}(\mathbf{H}^\top\mathbf{W}^\top\mathbf{W}\mathbf{H}) - \operatorname{Tr}(\mathbf{H}^\top\mathbf{W}^\top\mathbf{X}) + \frac{\gamma}{2}\|\mathbf{Y} - \mathbf{X}\mathbf{H}^\top\operatorname{Diag}(\boldsymbol{\beta})\|_F^2}_{:=f(\mathbf{H})} + \lambda \underbrace{\|\mathbf{H}\|_1}_{:=g(\mathbf{H})},$$

where g is a non-smooth penalty. For this reason, updates for $\mathbf{H}$ are obtained thanks to the proximal approach, as described in Section S2.1.2.

Combining Proposition 3 with update steps given in Equations (4) and (5), we obtain the update rule for $\mathbf{H}$ as:

$$\mathbf{H}^{(t+1)} = \operatorname{prox}_{\tilde{g}}\left(\tilde{\mathbf{H}}^{(t)}\right) = \left[\tilde{\mathbf{H}}^{(t)} - \frac{\lambda}{\eta}\right]_+ \tag{11}$$

where $\tilde{g} = \frac{\lambda}{\eta}\|\cdot\|_1 + \delta_+$ and

$$\tilde{\mathbf{H}}^{(t)} = \mathbf{H}^{(t)} - \frac{1}{\eta}\nabla f(\mathbf{H}^{(t)}). \tag{12}$$

In addition, using Equation (8), we get

$$\nabla f(\mathbf{H}) = \mathbf{W}^\top\mathbf{W}\mathbf{H} + \gamma\operatorname{Diag}(\boldsymbol{\beta}^2)\mathbf{H}\mathbf{X}^\top\mathbf{X} - \gamma\operatorname{Diag}(\boldsymbol{\beta})\mathbf{Y}^\top\mathbf{X} - \mathbf{W}^\top\mathbf{X}.$$

Plugging these in Equation (12), we finally have

$$\begin{aligned}\tilde{\mathbf{H}}^{(t)} &= \mathbf{H}^{(t)} - \frac{1}{\eta}\underbrace{\left[\mathbf{W}^\top\mathbf{W}\mathbf{H}^{(t)} + \gamma\operatorname{Diag}(\boldsymbol{\beta}^2)\mathbf{H}^{(t)}\mathbf{X}^\top\mathbf{X}\right]}_{:=\mathbf{D}(\mathbf{H}^{(t)})} + \frac{1}{\eta}\underbrace{\left[\gamma\operatorname{Diag}(\boldsymbol{\beta})\mathbf{Y}^\top\mathbf{X} + \mathbf{W}^\top\mathbf{X}\right]}_{:=\mathbf{R}} \\ &= \mathbf{H}^{(t)} + \frac{1}{\eta}\left[\mathbf{R} - \mathbf{D}(\mathbf{H}^{(t)})\right].\end{aligned}$$

S2.2.3 Update for $\beta^{(j)}$

For the sake of simplicity, we omit the step number t for matrices that are not updated (*i.e.*, $\mathbf{W}$ will be used for the current value of this matrix, $\mathbf{W}^{(t)}$, and similarly for $\mathbf{H}^{(j,t)}$). For the same reason, since the objective function for $\boldsymbol{\beta}^{(j)}$ is separable in j, indices j (related to the omics datasets) are omitted too (*i.e.*, $\boldsymbol{\beta}$ will refer to $\boldsymbol{\beta}^{(j)}$, $\mathbf{X}$ to $\mathbf{X}^{(j)}$, $\mathbf{H}$ to $\mathbf{H}^{(j)}$ and E to E_j).

Based on the one-hot encoding form, we re-write Equation (9) as:

$$E(\boldsymbol{\beta}) = \frac{\gamma}{2}\sum_{i=1}^{n}\sum_{k=1}^{K}\left(\mathbf{Y}_{ik} - \tilde{\mathbf{x}}_{ik}^\top\boldsymbol{\beta}_k\right)^2$$

where $\tilde{\mathbf{x}}_{ik} \in \mathbb{R}$ is the entry ik of $\mathbf{X}\mathbf{H}^\top$. Minimizing $E(\boldsymbol{\beta})$ thus corresponds to minimizing K independent OLS problems, which leads to the following update of $\boldsymbol{\beta}$ at each step of the algorithm:

$$\boldsymbol{\beta}_k^{(t+1)} = \frac{\mathbf{H}_{k.}\mathbf{X}^\top\mathbf{Y}_{.k}}{\mathbf{H}_{k.}\mathbf{X}^\top\mathbf{X}\mathbf{H}_{k.}^\top} = \frac{\overline{\mathbf{X}\mathbf{H}_{k.}^\top}^k}{\mathbf{H}_{k.}\mathbf{X}^\top\mathbf{X}\mathbf{H}_{k.}^\top},$$

where $\overline{\mathbf{X}\mathbf{H}_{k.}^\top}^k$ is the average of $\mathbf{X}\mathbf{H}_{k.}^\top$ across observations of goup k. This quantity is ensured to be positive.

S2.2.4 Backtracking line search

The backtracking line search is a common method determining the most suited step size in gradient descent procedures. The method begins with a relatively large step size and shrinks it iteratively until satisfying an adequate stopping criterion. This approach limits local optimum issues and accelerates convergence of algorithms. [Brouard et al., 2022] successfully used it in a Forward Backward Splitting (FBS) algorithms [Bauschke and Combettes, 2017] for kernels methods applied on omics datasets. Here, we adapted their procedure for the automatic setting of the hyperparameters $\eta^{(j)}$ used in the proximal update term of $\mathbf{H}^{(j)}$ (see Section S2.2.2).

S3 Information on datasets

S3.1 Simulated data

To generate simulated data with a clear ground truth, binary matrices, $\mathbf{W}$ and $\mathbf{H}^{(j)}$ ($\forall j \in \{1, 2\}$), were first generated. For both omics and both matrices, $K = 2$ signatures were generated. Each of the two signatures characterized one

of the two groups of samples, with a certain (chosen) selected number of non-zero features in each omics. Non-zero positive values were randomly drawn from $\mathcal{B}eta(2,2) \times 2$. They replaced non-zero entries in $\mathbf{W}$ and $\mathbf{H}^{(j)}$, producing $\hat{\mathbf{W}}$ and $\hat{\mathbf{H}}^{(j)}$. The omic datasets were obtained as $\widehat{\mathbf{X}}^{(j)} = \epsilon(\hat{\mathbf{W}}\hat{\mathbf{H}}^{(j)})$, where ϵ is a process introducing noise in the data in two different ways: 1) with a certain (chosen) probability, each value was either shrunk to 0 or drawn from a $\mathcal{B}eta$ distribution; 2) a matrix of random values drawn from a uniform distribution was added. If negative values were obtained during this process, they were flipped to their opposite.

A realistic batch noise was finally introduced using two datasets ($\widetilde{\mathbf{X}}^{(j)}$) simulated independently and similarly. Hence, $\widehat{\mathbf{X}}^{(j)}$ were used to feature the "true" group structure and $\widetilde{\mathbf{X}}^{(j)}$ were used to feature a 2-level "batch" effect (the distribution of rows between levels of the group and of the batch were different). The final dataset was obtained as the concatenation of the columns of the two datasets.

The flexible framework of this data generation procedure allowed us to challenge NMFProfiler on various aspects. In a nutshell, simulated data were varied on their percentage of noisy features (*i.e.*, on the number of features not selected in either of the $K = 2$ signatures from the true group structure or the batch effect), on the proportion taken by batch effect, and on the groups size. All tested data designs are summarized in Table S1. Simulated dataset 00 stands as a baseline (all parameters are set so as to generate the simplest case, *i.e.* a case with neither noise nor batch pattern and not any disequilibrium). Simulated dataset 02 is the framework discussed in the article, which corresponds to the following parameters: $n_1 = n_2 = n/2$ (groups perfectly balanced), $\hat{p}_1 = \tilde{p}_1 = p_1/2$, $\hat{p}_2 = \tilde{p}_2 = p_2/2$ (group and batch patterns of the same size), $\omega = \phi = 0$ (case where irrelevant features all come from "batch" pattern features : *i.e.*, Nb. main patterns = 2 and % noisy features = 0), and with a variance of $\epsilon()$ larger than the variance of $\tilde{\epsilon}()$ (hence with a weaker noise for the batch effect than for the group effect).

Table S1: **Simulated data.** Settings used to generate the different simulated datasets. For scenarios with two main patterns, the first pattern corresponds to the group of interest and the second is meant to represent an external variation of no interest for the user, which we abusively called "batch" effect. Across simulations, dimensions of $\mathbf{X}^{(j)}$s are fixed to $n = 50$, $p_1 = 2500$, and $p_2 = 400$. σ_u corresponds to the probability to add a uniform error term (see main text).

Simulated dataset nb.	Nb. main patterns	% pat= 1 in $\mathbf{X}^{(j)}$	% noisy features	σ_u (or $\sigma_u^{(pat)}$)	Group size n_u
n°00	1	100	0	0.5	$n_1 = n_2$
n°01	2	20	0	$\sigma_u^{(1)} = 0.5, \sigma_u^{(2)} = 0.2$	$n_2 = n_1$
**n°02**	**2**	**50**	**0**	$\sigma_u^{(1)} = 0.5, \sigma_u^{(2)} = 0.2$	$n_2 = n_1$
n°03	-	75	-	-	-
n°04	-	90	-	-	-
n°05	1	90	10	0.5	$n_2 = n_1$
n°06	-	50	50	-	-
n°07	-	20	80	-	-
n°08	2	45	10	$\sigma_u^{(1)} = 0.5, \sigma_u^{(2)} = 0.2$	$n_2 = n_1$
n°09	-	25	50	-	-
n°10	-	10	80	-	-
n°11	1	90	10	0.5	$n_2 = 2n_1$
n°12	-	-	-	-	$n_2 = 3n_1$
n°13	2	50	0	0.5	$n_2 = 2n_1$
n°14	-	-	-	-	$n_2 = 3n_1$

S3.2 Colon adenocarcinoma study (TCGA)

TCGA-COAD data were downloaded from http://acgt.cs.tau.ac.il/multi_omic_benchmark/download.html [Rappoport and Shamir, 2018]. The $n = 221$ samples to three omics were first extracted, corresponding to:

- gene expression (RNAseqv2 level 3 RSEM genes normalized), denoted `gene`;
- DNA methylation (Illumina-450k level 3), denoted `methyl`;
- miRNA expression (Illumina mirnaseq level 3 miR gene expression), `miRNA`.

We found no missing values in any of the three datasets but we removed features with (nearly) null variance, using the function `nearZeroVar()` from the R package **mixOmics**. Gene and miRNA expression datasets were also log-transformed. We ended up with $p_{\texttt{gene}} = 18,877$, $p_{\texttt{methyl}} = 5,000$, and $p_{\texttt{miRNA}} = 503$ features in each of the three omics.

In addition, samples are characterized based on the extent of the primary tumor (pathologic Tc), the extent of the distant metastasis (pathologic Mc), and the extent of the regional lymph node involvement (pathologic Nc). We recoded these clinical variables in three groups each:

- T: T2, T3, and T4 that indicate size and /or extent of the primary tumor;
- M: M0 (no distant metastasis), M1 (distant metastasis present), and MX (distant metastasis could not be evaluated);
- N: N0 (no cancer found in regional lymph nodes), N1, and N2 (different levels of number and / or extent of spread of cancer in regional lymph nodes).

DIABLO signatures are only easily associated to a specific group for the case of the comparison of two groups. Hence, to allow the direct comparison between NMFProfiler and DIABLO, we also derived from the previous recoded clinical variables new binary clinical variables corresponding to subcases comparisons:

- T2vsT3 (comparison between levels T2 and T3 of T) and T2vsT4;
- M0vsM1 and M0vsMX;
- N0vsN1 and N0vsN2.

In addition, the survival time (in number of days) and the death status were also downloaded on the same data repository and all samples with a missing value in the (recoded) clinical feature of interest and / or survival features were removed.

Final sample sizes of each of the are provided in Table S2.

Table S2: **TCGA-COAD.** Group sizes of recoded clinical features.

Clinical feature	Group	Number of samples
T	T2	29
	T3	155
	T4	24
M	M0	153
	M1	25
	MX	33
N	N0	124
	N1	55
	N2	35

S3.3 AD study

Proteomics Proteomic analysis was realized following the shotgun bottom-up approach using a Proxeon EASY-nLC II UPLC system coupled to a Q-Exactive MS instrument (Thermo Fisher Scientific). Prior to injection, the protein extracts were subjected to the modified Filter-Aided Sample Preparation method. The instrument was set to acquire top 10 MS/MS. Protein tandem MS/MS data were processed using MaxQuant, and the peptides were identified by performing target-decoy searches against the Uniprot Rattus norvegicus database concatenated with known contaminants. Identifications were filtered using false discovery rate (FDR) thresholds at both peptide and protein levels. MaxQuant and Scaffold were used for peptide/protein grouping, label-free quantification. The total spectrum count of $1,303$ protein clusters were measured. Total Counts were $\log_2$ transformed and data were then normalized using quantile normalization. The seventeen protein clusters with no associated gene name were removed. Low count and low variance proteins were also filtered out. Thus the analysis was performed on 281 clusters of proteins. Data were then batch-corrected for different LC-MS/MS analysis dates using the method ComBat [Johnson et al., 2007] and the efficacy of the correction was validated using Principal Component Analysis (PCA).

Microarray The transcriptomic analysis was performed with Human Gene Array Plates (Human Gene ST Array 2.1 Affymetrix). Data were normalized using the Robust Multi-Array Average (RMA) method [Irizarry et al., 2003] and unexpressed probes were removed, following the procedure described in **limma** user guide. The latter filter led to a strong decrease in the number of probe sets. Only the probe sets with a non-missing *entrez gene id* annotation were kept for subsequent analyses. Thus the analysis was performed on $p_{\text{tran}} = 1,847$ clusters of probeset genes. Data were then batch-corrected for different hybridation dates using the same method ComBat [Johnson et al., 2007] as for proteomics and the correction was also validated using PCA.

The final dataset consisted on $n = 26$ samples for proteomic and $n = 28$ samples for transcriptomic data, usually including two samples per subject (one coming from the left inner forearm and the other coming from the right inner forearm). For multi-omics analysis, only samples corresponding to the left forearm and with both omics available for the corresponding subject were used ($n = 12$).

S4 Information on implementation of tested methods

Hyperparameters (when present) were chosen as follows: For NMF variants, hyperparameters in Equation (4) were automatically set to a default value, defined based on preliminary tests on independent data and depending on the dataset characteristics: $\mu = 10^{-3}$, $\lambda = n \times 10^{-3}$, and $\gamma = n \times 10^{-2}$ [1]. The gradient step size, $\eta^{(j)}$, in the proximal version was initialized to 1 but automatically adjusted using a backtrack linesearch approach (see Section S2.2.4 of the Supplementary material).

For DIABLO variants, the design matrix $\mathbf{C}$ was set to its recommended value $\begin{pmatrix} 0 & 0.1 & 1 \\ 0.1 & 0 & 1 \\ 1 & 1 & 0 \end{pmatrix}$. In addition, in the sparse variant, the number of selected features in the signature was obtained by cross-validation (CV) using `tune.block.splsda()` over the grid $\{10, 50, 100, 250, 500, 625, 750, 1000, 1250, 1500, 1875, 2000, 2250, 2500\}$ and $\{10, 40, 80, 100, 150, 200, 250, 300, 360, 400\}$, respectively for the first and second omics. Finally, for MOFA, we used default values except for `scale_views = TRUE` (to be consistent with DIABLO), `spikeslab_weights = FALSE` (to get approximate sparsity) and `num_factors = 2` (because we are interested in only the first component as discussed before).

Centering and scaling to unit variance is not indicated for NMF so, in these methods, potential size effects were controlled by scaling all features $\mathbf{X}^{(j)}$ between 0 and 1 through a min-max approach and by dividing each omic dataset by $\sqrt{p_j}$. For the sake of completeness, MOFA and DIABLO were also tested with this pre-processing but this led to poorer results for these methods.

S5 Supplementary results

S5.1 Additional results for the main simulated dataset (Simulated dataset 02)

S5.1.1 Simulated data

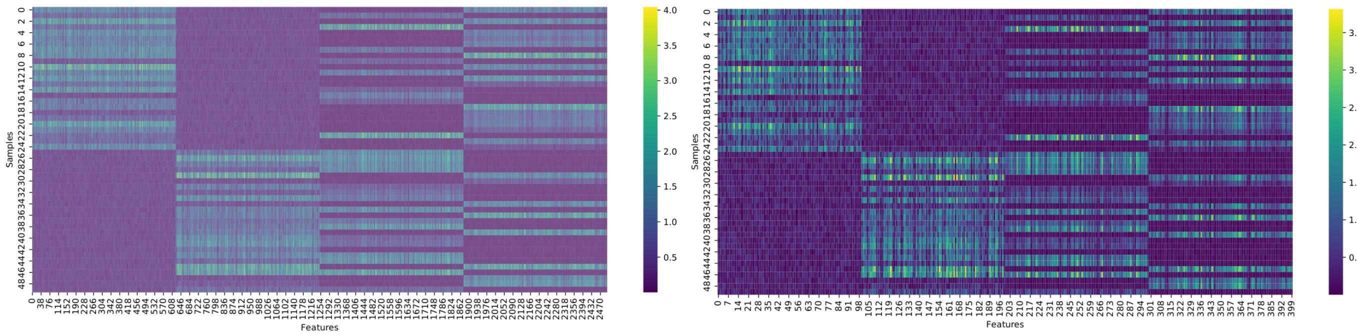

(a) $\mathbf{X}^{(1)}$ (b) $\mathbf{X}^{(2)}$

Figure S3: **Simulated dataset 02.** Heatmaps of $\mathbf{X}^{(j)}$ (one over 50 simulations).

[1]Default hyperparameters of the `scikit-learn` NMF are similar to ours.

S5.1.2 Assessment of feature selection quality

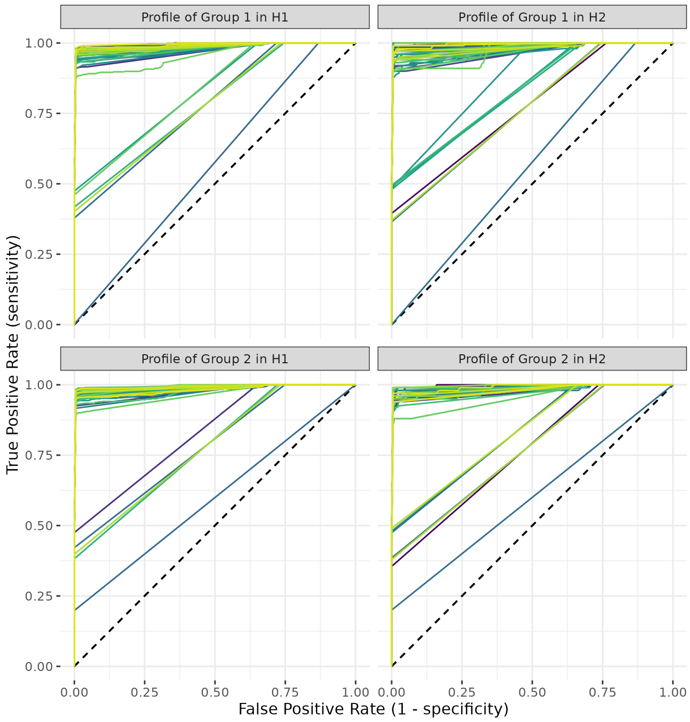

Figure S4: **Simulated dataset 02.** Individual ROC curves for DIABLO (50 simulations). The dashed line corresponds to the ROC of a random classifier.

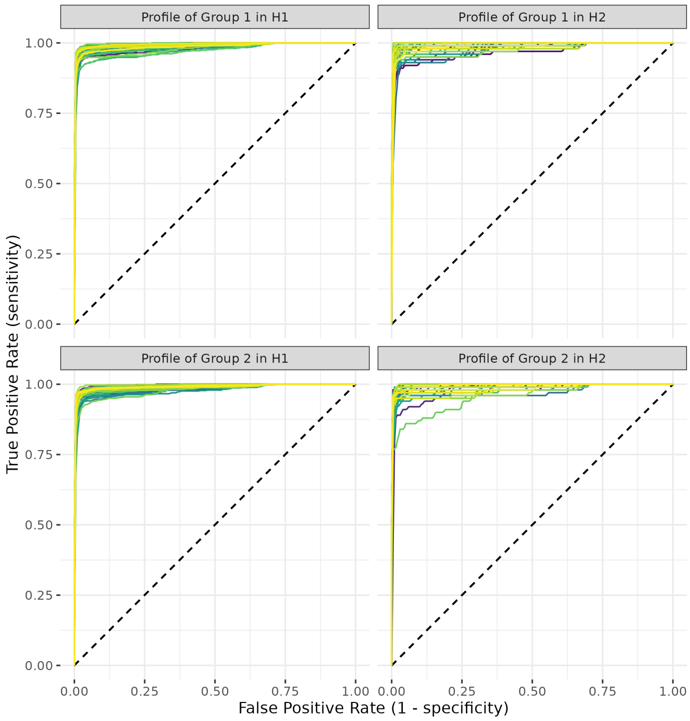

Figure S5: **Simulated dataset 02.** Individual ROC curves for NMFProfiler-MU (50 simulations). The dashed line corresponds to the ROC of a random classifier.

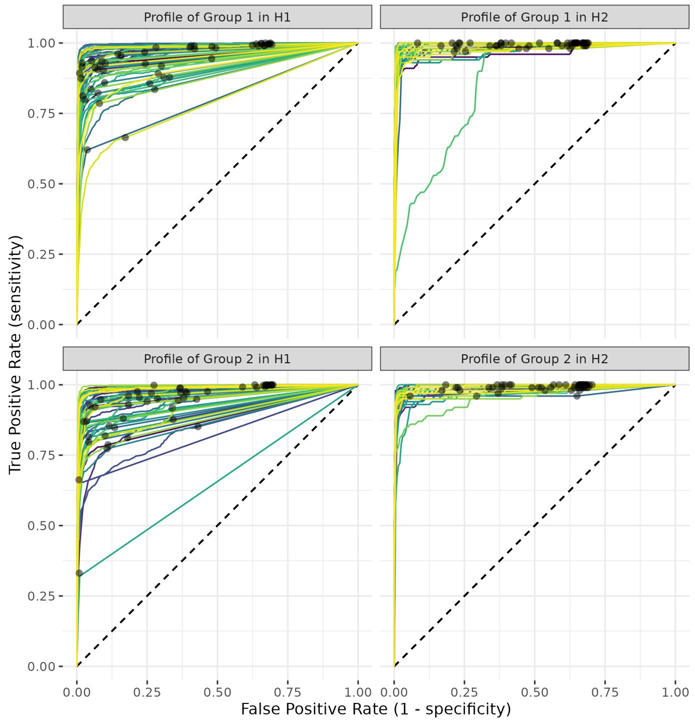

Figure S6: **Simulated dataset 02.** Individual ROC curves for NMFProfiler-prox (50 simulations). The dashed line corresponds to the ROC of a random classifier. Black dots correspond to the TPR and FPR obtained with a threshold of 0, which is the level of sparsity produced automatically by the method.

S5.1.3 Assessment of exact sparsity quality

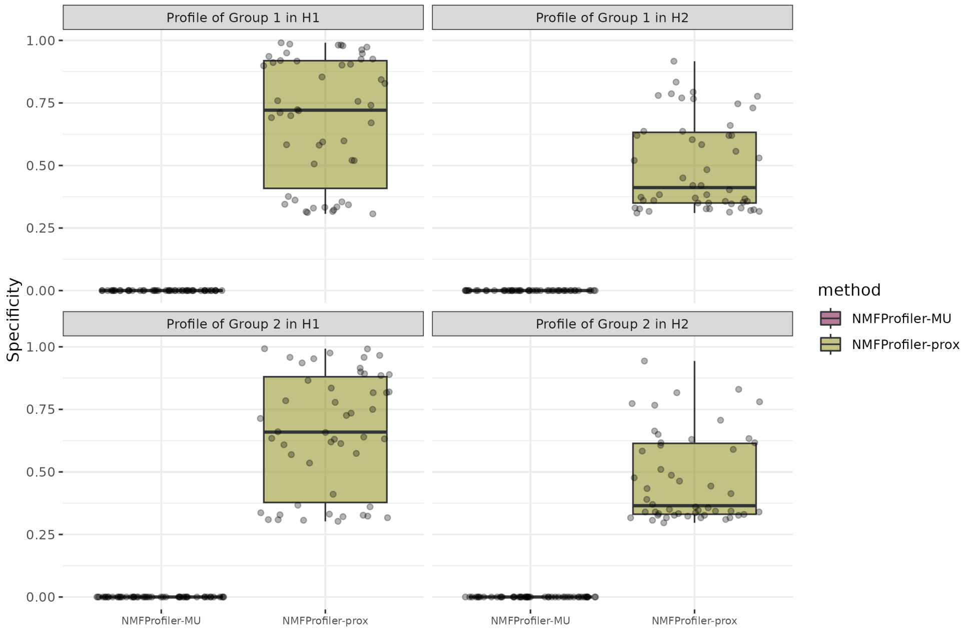

Figure S7: **Simulated dataset 02.**. Specificity of NMFProfiler variants (50 simulations).

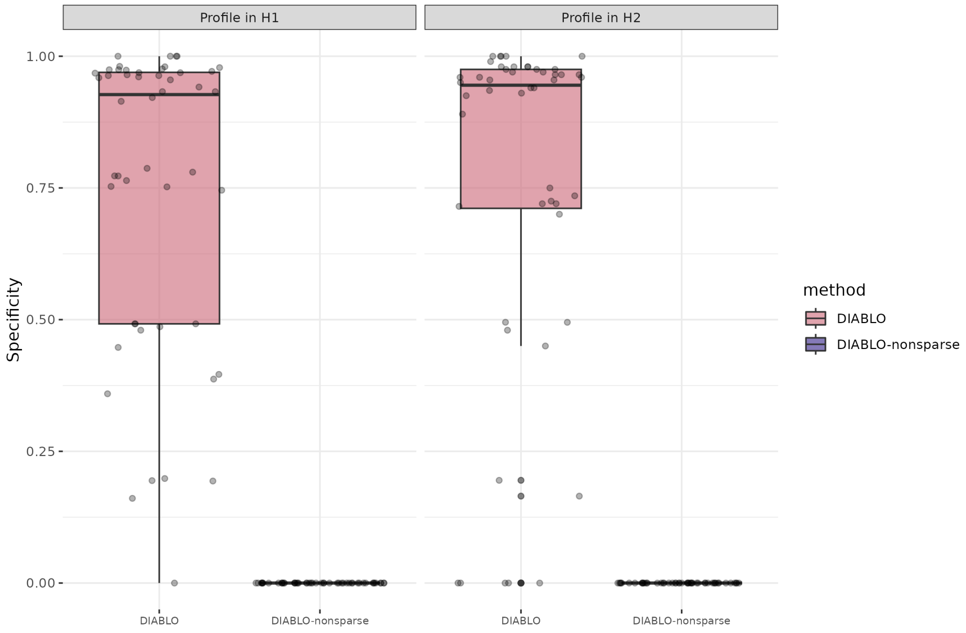

Figure S8: **Simulated dataset 02.** Specificity of DIABLO variants (50 simulations).

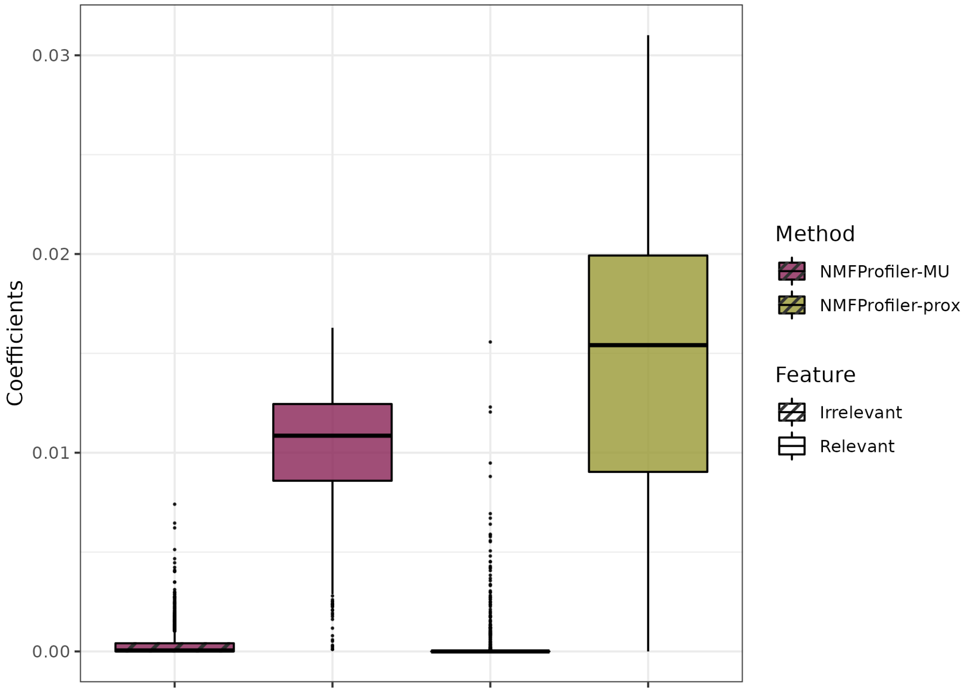

Figure S9: **Simulated dataset 02.** Distribution of coefficients in dictionary matrices $\mathbf{H}^{(j)}$ for dataset 1, group 1 for NMFProfiler variants (one simulation over 50).

S5.1.4 Assessment of sample classification quality

For each omic j, a logistic regression of y on the projection of the individuals onto the signature matrix ($i.e.$, $\mathbf{X}^{(j)}\mathbf{H}^{(j)\top}$) has been fitted. Classification accuracy and McFadden index have been computed using a $5-$fold CV strategy.

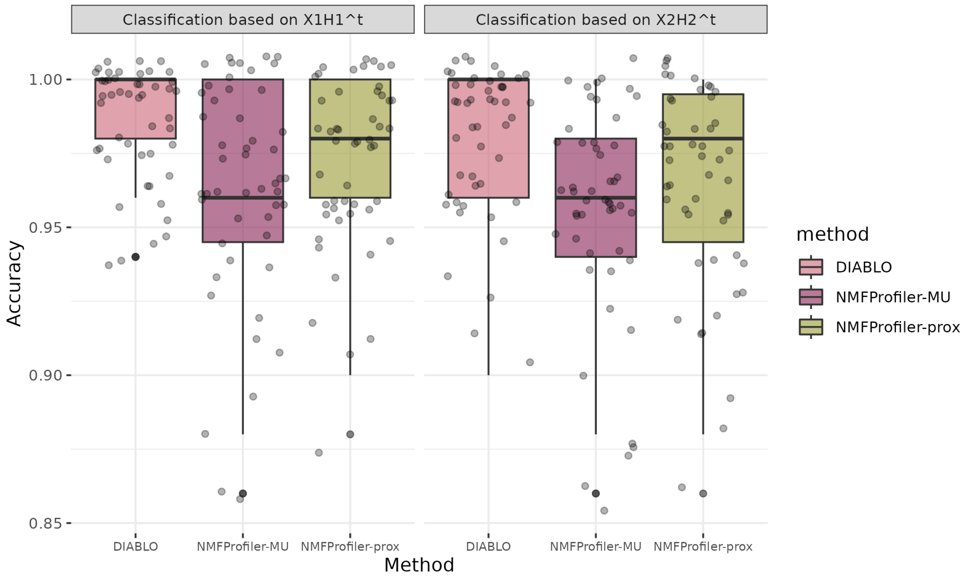

Figure S10: **Simulated dataset 02.** Classification accuracy in logistic regression from features selected by supervised methods (except DIABLO-nonsparse) over 50 simulations.

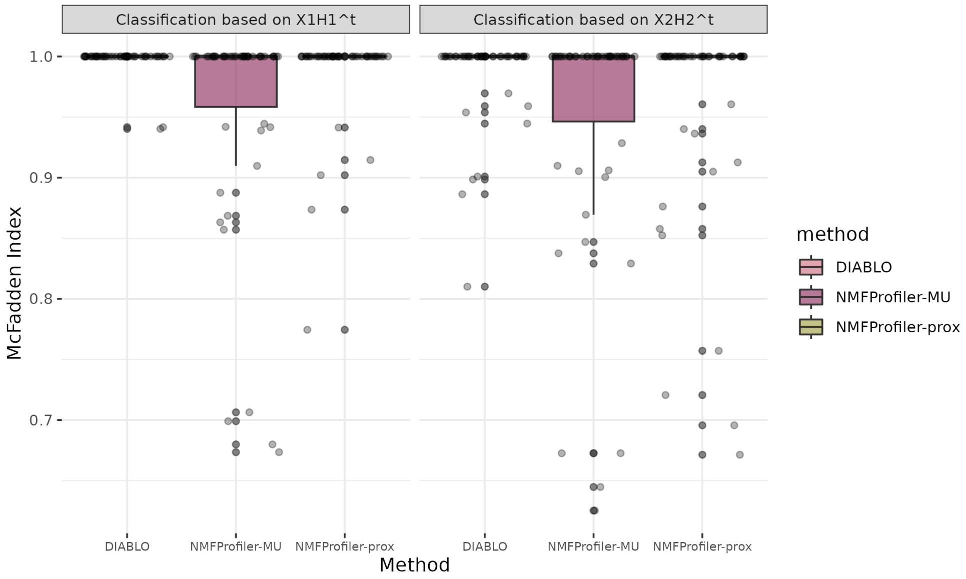

Figure S11: **Simulated dataset 02.** McFadden index (also called pseudo-R^2) obtained in logistic regression from features selected by supervised methods (except DIABLO-nonsparse) over 50 simulations.

S5.1.5 Computational time

Computational time required to run each method on 50 simulated pairs of datasets is measured.

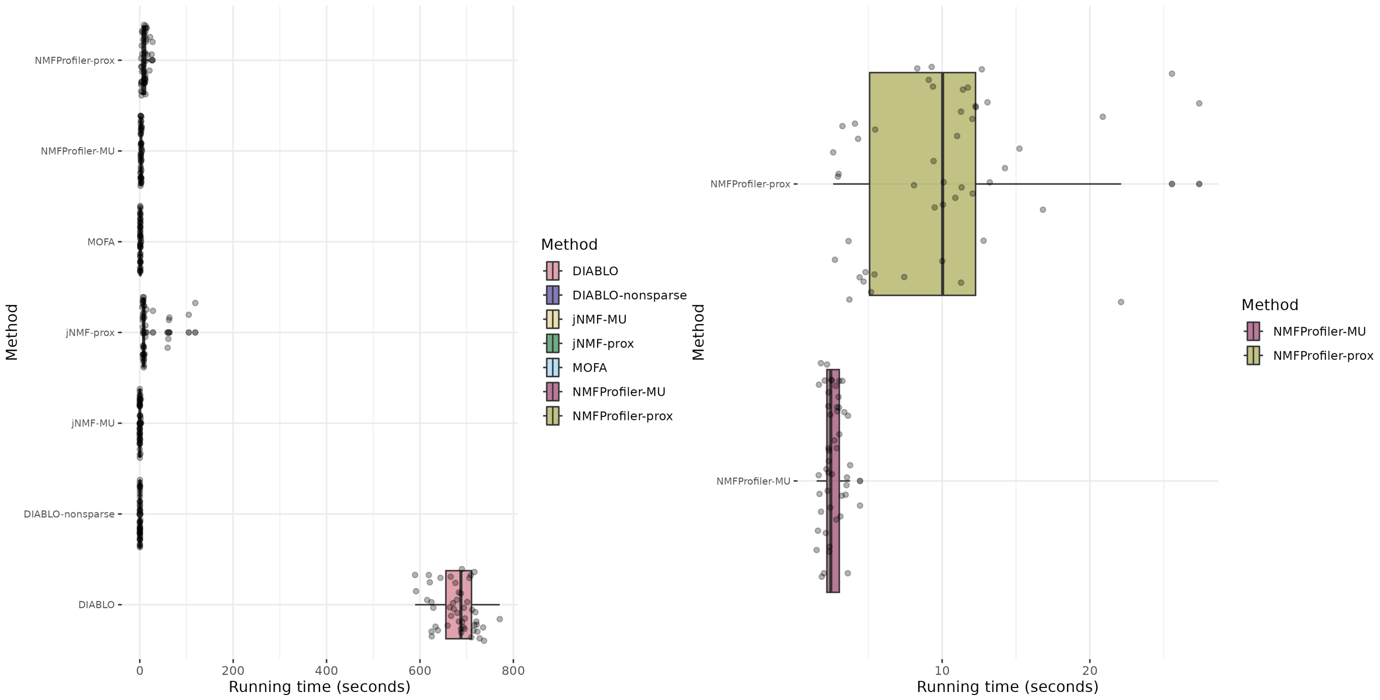

(a) All methods

(b) NMFProfiler with new supervised term

Figure S12: **Simulated dataset 02.** Computational time of the different methods (50 simulations).

S5.2 Additional results obtained on simulated data (other frameworks)

S5.2.1 Feature selection: numeric performance (AUROCs, 50 simulations)

Table S3: **Simulated data.** Averaged AUROCs (and standard deviation) given simulation scenario number, dataset and group.

Simulated dataset nb.	j	u	**AUC**						
			jNMF		MOFA	DIABLO		NMFProfiler	
			MU	prox		nonsparse	sparse	MU	prox
n°00	1	1	0.720 (0.242)	0.976 (0.115)	**1.000** (0.000)	**1.000** (0.000)	0.885 (0.170)	0.997 (0.002)	**0.993** (0.005)
	1	2	0.730 (0.233)	0.979 (0.098)	**1.000** (0.000)	**1.000** (0.000)	0.885 (0.170)	0.997 (0.002)	**0.992** (0.006)
	2	1	0.718 (0.244)	0.976 (0.114)	**1.000** (0.000)	**1.000** (0.000)	0.821 (0.208)	0.998 (0.002)	**0.997** (0.003)
	2	2	0.727 (0.236)	0.979 (0.098)	**1.000** (0.000)	**1.000** (0.000)	0.821 (0.208)	0.997 (0.002)	**0.997** (0.003)
n°01	1	1	0.505 (0.037)	0.525 (0.036)	0.539 (0.040)	0.549 (0.016)	**0.946** (0.111)	**0.985** (0.009)	0.916 (0.039)
	1	2	0.506 (0.038)	0.524 (0.040)	0.543 (0.037)	0.550 (0.015)	**0.946** (0.107)	**0.983** (0.011)	0.923 (0.037)
	2	1	0.514 (0.042)	0.522 (0.034)	0.539 (0.041)	0.543 (0.023)	0.952 (0.099)	**0.989** (0.013)	**0.963** (0.034)
	2	2	0.515 (0.047)	0.527 (0.043)	0.544 (0.038)	0.545 (0.021)	0.939 (0.108)	**0.975** (0.021)	**0.955** (0.031)
n°02	1	1	0.541 (0.074)	0.647 (0.115)	0.720 (0.096)	0.675 (0.010)	**0.974** (0.062)	**0.990** (0.005)	0.946 (0.048)
	1	2	0.538 (0.063)	0.640 (0.111)	0.712 (0.092)	0.673 (0.006)	**0.978** (0.044)	**0.990** (0.004)	0.942 (0.059)
	2	1	0.543 (0.074)	0.650 (0.106)	0.721 (0.095)	0.673 (0.007)	0.971 (0.064)	**0.991** (0.008)	**0.985** (0.021)
	2	2	0.543 (0.064)	0.648 (0.101)	0.712 (0.088)	0.671 (0.005)	0.974 (0.047)	**0.991** (0.008)	**0.987** (0.010)
n°03	1	1	0.584 (0.157)	0.939 (0.134)	0.975 (0.020)	**0.991** (0.006)	**0.992** (0.004)	0.990 (0.005)	0.956 (0.051)
	1	2	0.588 (0.156)	0.940 (0.138)	0.974 (0.020)	**0.991** (0.005)	**0.992** (0.004)	**0.991** (0.005)	0.949 (0.053)
	2	1	0.580 (0.163)	0.932 (0.146)	0.975 (0.021)	0.991 (0.007)	0.969 (0.078)	**0.992** (0.006)	**0.990** (0.007)
	2	2	0.586 (0.159)	0.942 (0.123)	0.974 (0.022)	0.991 (0.007)	0.968 (0.078)	**0.993** (0.005)	**0.990** (0.007)
n°04	1	1	0.719 (0.232)	0.968 (0.118)	0.994 (0.004)	**0.997** (0.001)	**0.983** (0.069)	0.992 (0.006)	0.975 (0.030)
	1	2	0.716 (0.235)	0.968 (0.116)	0.994 (0.004)	**0.997** (0.002)	**0.983** (0.068)	0.992 (0.007)	0.973 (0.033)
	2	1	0.720 (0.233)	0.969 (0.112)	0.994 (0.004)	**0.997** (0.002)	0.960 (0.096)	0.994 (0.005)	**0.992** (0.006)
	2	2	0.716 (0.235)	0.977 (0.088)	0.994 (0.005)	**0.997** (0.002)	0.960 (0.098)	0.995 (0.006)	**0.993** (0.007)
n°05	1	1	0.725 (0.236)	0.944 (0.110)	**0.997** (0.001)	**0.997** (0.001)	0.946 (0.139)	0.972 (0.010)	**0.958** (0.036)
	1	2	0.724 (0.241)	0.943 (0.123)	**0.997** (0.001)	**0.997** (0.001)	0.945 (0.139)	0.973 (0.011)	**0.958** (0.033)
	2	1	0.726 (0.237)	0.953 (0.099)	**0.997** (0.002)	**0.997** (0.002)	0.896 (0.173)	0.976 (0.011)	**0.968** (0.014)
	2	2	0.728 (0.238)	0.949 (0.113)	**0.997** (0.002)	**0.997** (0.002)	0.898 (0.170)	0.975 (0.012)	**0.966** (0.016)

Table S3: **Simulated data.** Averaged AUROCs (and standard deviation) given simulation scenario number, dataset and group.

Simulated dataset nb.	j	u	**AUC**						
			jNMF		MOFA	DIABLO		NMFProfiler	
			MU	prox		nonsparse	sparse	MU	prox
n°06	1	1	0.864 (0.109)	0.742 (0.218)	**0.991** (0.003)	0.990 (0.003)	**0.981** (0.018)	0.887 (0.104)	0.836 (0.128)
	1	2	0.868 (0.136)	0.733 (0.231)	**0.991** (0.003)	0.990 (0.003)	**0.981** (0.017)	0.908 (0.088)	0.880 (0.099)
	2	1	0.870 (0.112)	0.746 (0.226)	**0.991** (0.006)	0.990 (0.006)	**0.967** (0.074)	0.888 (0.107)	0.889 (0.103)
	2	2	0.875 (0.138)	0.734 (0.240)	**0.991** (0.006)	0.990 (0.006)	**0.967** (0.073)	0.911 (0.087)	0.928 (0.063)
n°07	1	1	0.749 (0.238)	0.447 (0.070)	**0.987** (0.006)	0.985 (0.006)	**0.964** (0.063)	0.630 (0.345)	0.710 (0.103)
	1	2	0.681 (0.278)	0.421 (0.100)	**0.988** (0.005)	0.987 (0.005)	**0.967** (0.062)	0.659 (0.356)	0.696 (0.115)
	2	1	0.762 (0.228)	0.449 (0.088)	**0.985** (0.014)	0.982 (0.012)	**0.974** (0.017)	0.631 (0.343)	0.604 (0.335)
	2	2	0.696 (0.264)	0.422 (0.080)	**0.986** (0.011)	0.985 (0.012)	**0.978** (0.014)	0.658 (0.360)	0.621 (0.311)
n°08	1	1	0.523 (0.063)	0.611 (0.106)	0.740 (0.098)	0.710 (0.009)	**0.977** (0.042)	**0.977** (0.010)	0.938 (0.044)
	1	2	0.527 (0.068)	0.605 (0.112)	0.740 (0.100)	0.710 (0.011)	**0.972** (0.060)	**0.977** (0.010)	0.936 (0.052)
	2	1	0.528 (0.070)	0.617 (0.104)	0.741 (0.096)	0.708 (0.012)	0.966 (0.062)	**0.979** (0.013)	**0.972** (0.013)
	2	2	0.530 (0.068)	0.600 (0.112)	0.740 (0.102)	0.708 (0.013)	**0.966** (0.062)	**0.979** (0.013)	0.965 (0.039)
n°09	1	1	0.504 (0.187)	0.531 (0.169)	0.814 (0.125)	0.848 (0.027)	**0.974** (0.020)	**0.851** (0.150)	0.814 (0.110)
	1	2	0.494 (0.184)	0.529 (0.178)	0.794 (0.143)	**0.840** (0.030)	**0.971** (0.016)	0.810 (0.170)	0.796 (0.110)
	2	1	0.508 (0.194)	0.535 (0.175)	0.818 (0.123)	0.841 (0.032)	**0.956** (0.072)	**0.850** (0.153)	0.891 (0.126)
	2	2	0.495 (0.184)	0.530 (0.185)	0.793 (0.147)	**0.834** (0.036)	**0.956** (0.075)	0.811 (0.169)	0.861 (0.133)
n°10	1	1	0.520 (0.233)	0.431 (0.056)	0.858 (0.195)	**0.943** (0.025)	**0.966** (0.019)	0.680 (0.329)	0.773 (0.104)
	1	2	0.495 (0.231)	0.425 (0.064)	0.897 (0.131)	**0.945** (0.021)	**0.966** (0.016)	0.712 (0.312)	0.766 (0.155)
	2	1	0.533 (0.233)	0.436 (0.092)	0.854 (0.197)	**0.932** (0.038)	**0.947** (0.088)	0.672 (0.326)	0.610 (0.261)
	2	2	0.511 (0.232)	0.433 (0.071)	0.894 (0.120)	**0.934** (0.030)	**0.952** (0.058)	0.714 (0.318)	0.595 (0.278)
n°11	1	1	0.817 (0.224)	0.954 (0.026)	**0.997** (0.001)	0.996 (0.001)	**0.981** (0.075)	0.991 (0.003)	0.963 (0.069)
	1	2	0.900 (0.156)	0.982 (0.006)	**0.997** (0.001)	**0.997** (0.001)	**0.980** (0.075)	0.915 (0.024)	0.897 (0.025)
	2	1	0.815 (0.230)	0.958 (0.027)	**0.996** (0.003)	**0.996** (0.002)	0.967 (0.086)	0.992 (0.004)	**0.978** (0.047)
	2	2	0.904 (0.151)	0.984 (0.006)	**0.997** (0.002)	**0.997** (0.002)	**0.964** (0.091)	0.923 (0.025)	0.911 (0.026)

Table S3: **Simulated data.** Averaged AUROCs (and standard deviation) given simulation scenario number, dataset and group.

Simulated dataset nb.	j	u	AUC jNMF MU	AUC jNMF prox	AUC MOFA	AUC DIABLO nonsparse	AUC DIABLO sparse	AUC NMFProfiler MU	AUC NMFProfiler prox
n°12	1	1	0.813 (0.231)	0.896 (0.171)	**0.996** (0.001)	**0.996** (0.001)	0.939 (0.152)	0.993 (0.002)	**0.968** (0.034)
	1	2	0.921 (0.135)	0.956 (0.089)	**0.997** (0.001)	**0.997** (0.001)	**0.938** (0.153)	0.874 (0.029)	0.847 (0.041)
	2	1	0.816 (0.234)	0.899 (0.185)	**0.996** (0.002)	**0.996** (0.002)	0.931 (0.151)	0.994 (0.002)	**0.975** (0.012)
	2	2	0.923 (0.135)	0.952 (0.112)	**0.998** (0.001)	0.997 (0.001)	**0.930** (0.155)	0.878 (0.033)	0.877 (0.031)
n°13	1	1	0.492 (0.111)	0.591 (0.124)	0.732 (0.122)	0.677 (0.024)	**0.962** (0.096)	**0.979** (0.017)	0.886 (0.090)
	1	2	0.674 (0.142)	0.767 (0.152)	0.730 (0.120)	0.674 (0.014)	**0.959** (0.100)	**0.991** (0.006)	0.929 (0.044)
	2	1	0.495 (0.111)	0.585 (0.131)	0.732 (0.123)	0.673 (0.018)	0.957 (0.099)	**0.981** (0.019)	**0.962** (0.025)
	2	2	0.679 (0.136)	0.772 (0.137)	0.731 (0.122)	0.672 (0.016)	0.950 (0.114)	**0.992** (0.007)	**0.991** (0.008)
n°14	1	1	0.493 (0.128)	0.560 (0.147)	0.711 (0.113)	0.672 (0.012)	**0.968** (0.073)	0.964 (0.061)	0.770 (0.156)
	1	2	0.742 (0.161)	0.831 (0.171)	0.733 (0.121)	0.671 (0.014)	**0.961** (0.078)	**0.980** (0.020)	0.907 (0.066)
	2	1	0.494 (0.130)	0.559 (0.148)	0.711 (0.114)	0.669 (0.015)	**0.964** (0.071)	**0.967** (0.058)	0.929 (0.047)
	2	2	0.747 (0.154)	0.832 (0.169)	0.734 (0.125)	0.670 (0.016)	0.948 (0.093)	**0.981** (0.025)	**0.980** (0.025)

S5.2.2 Feature selection: ROC curves (50 simulations)

Median ROC curves with their interquartile range ribbon for each dataset j and each group u obtained with several multi-omics analysis methods. Note that for some methods, computations were not made on 50 simulations but less because of a very few number of failures.

Basis without batch pattern

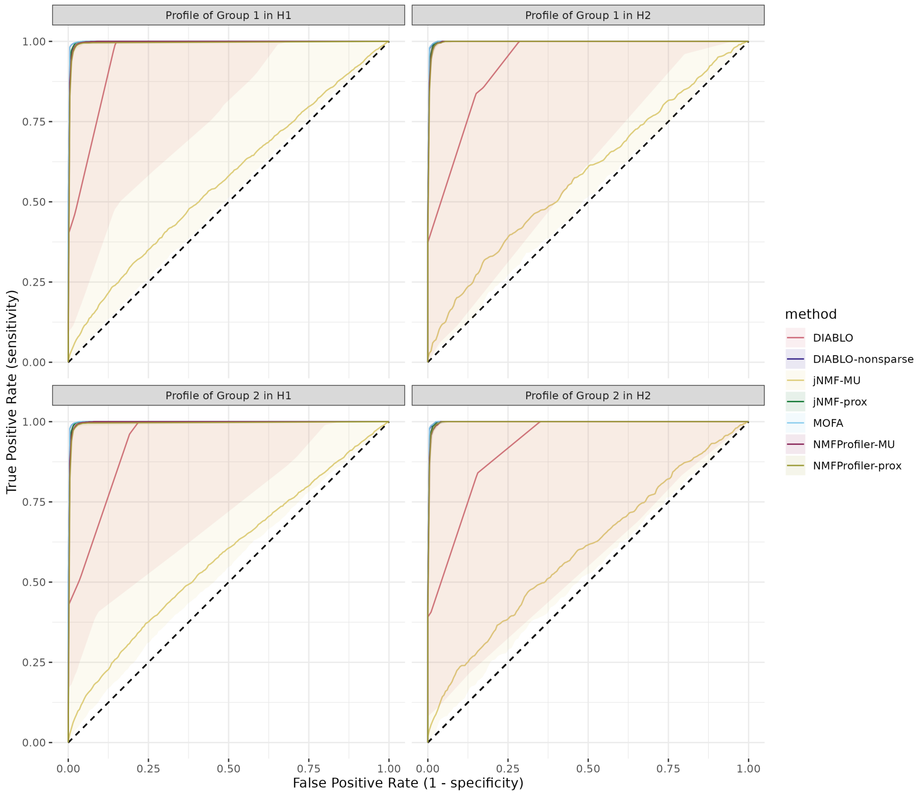

Figure S13: **Simulated dataset 00.** Median ROC curves. The ribbon corresponds to the interquartile range over the 50 simulations. The dashed line corresponds to the ROC of a random classifier.

Variation of the size of the batch pattern (column "% pat=1 in $\mathbf{X}^{(j)}$")

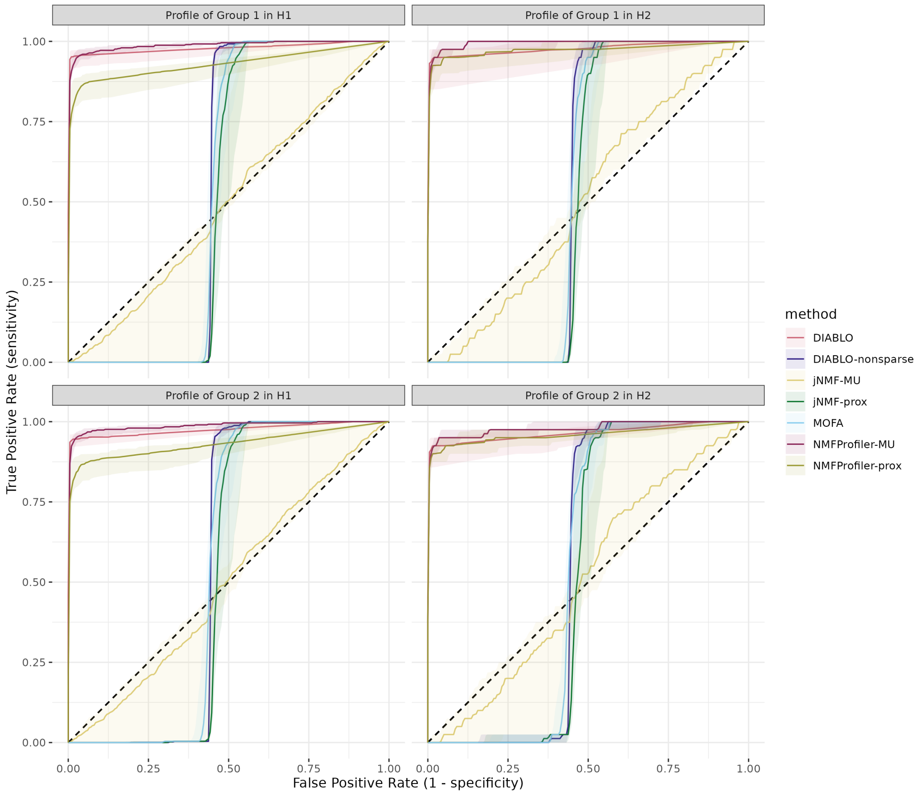

Figure S14: **Simulated dataset 01.** Median ROC curves. The ribbon corresponds to the interquartile range over the 50 simulations. The dashed line corresponds to the ROC of a random classifier.

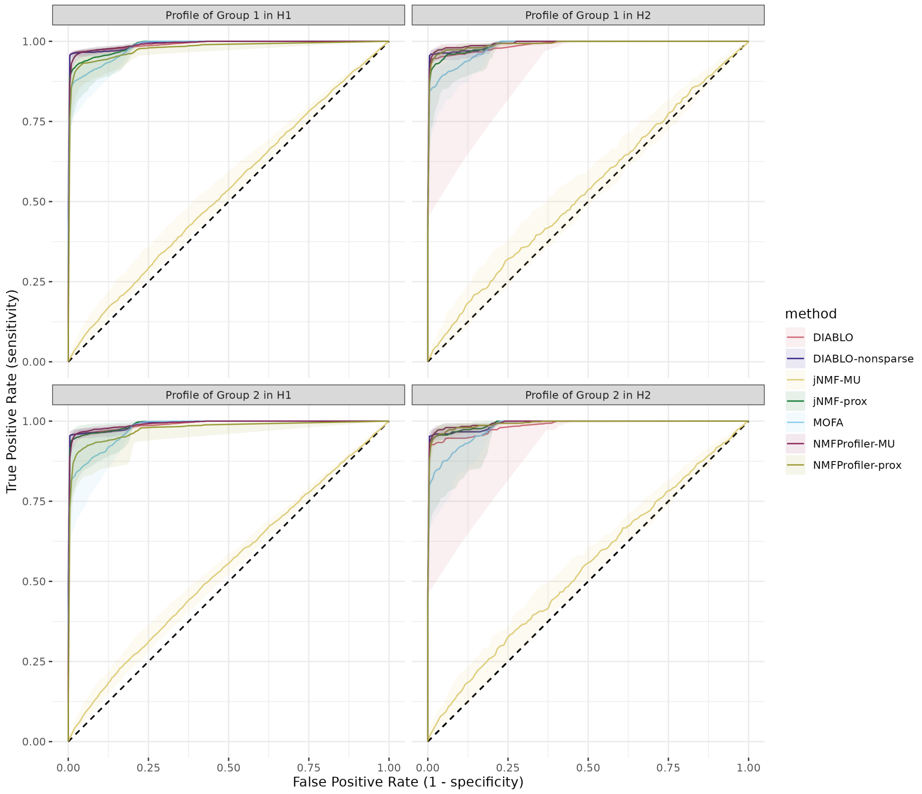

Figure S15: **Simulated dataset 03.** Median ROC curves. The ribbon corresponds to the interquartile range over the 50 simulations. The dashed line corresponds to the ROC of a random classifier.

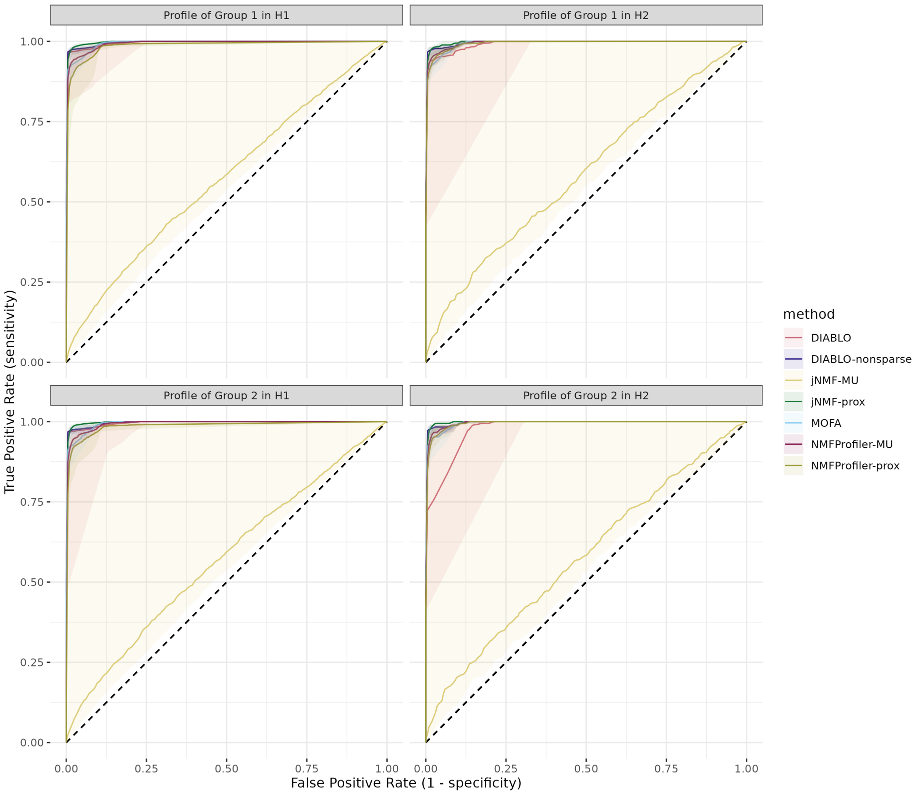

Figure S16: **Simulated dataset 04.** Median ROC curves. The ribbon corresponds to the interquartile range over the 50 simulations. The dashed line corresponds to the ROC of a random classifier.

Variation of the percentage of features in the batch pattern and of the percentage of noisy features (columns "% pat=1 in $\mathbf{X}^{(j)}$" and "% noisy features")

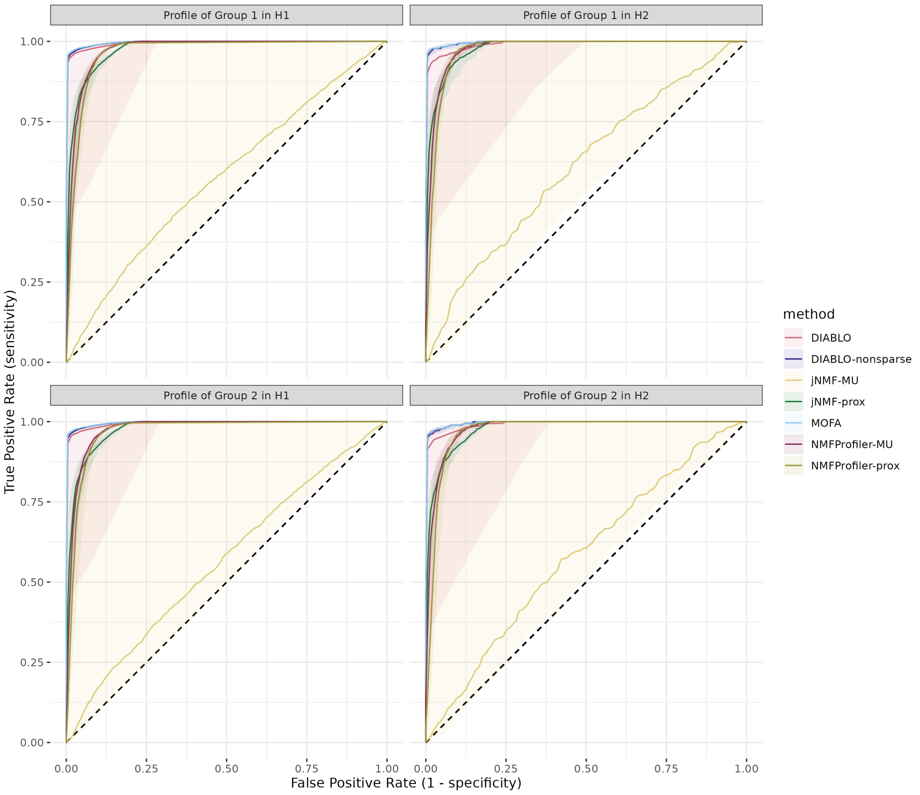

Figure S17: **Simulated dataset 05.** Median ROC curves. The ribbon corresponds to the interquartile range over the 50 simulations. The dashed line corresponds to the ROC of a random classifier.

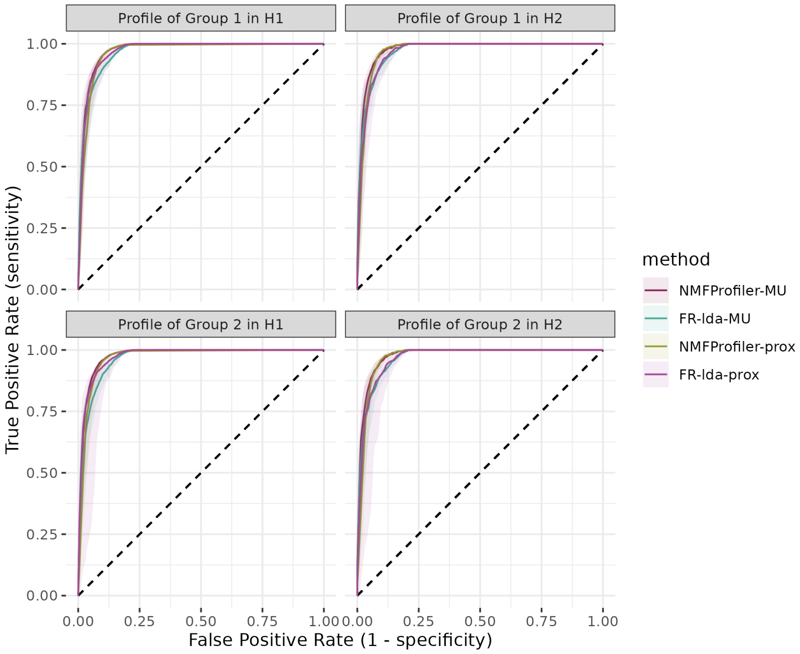

Figure S18: **Simulated dataset 05.** Median ROC curves for the different supervised NMF variants. The ribbon corresponds to the interquartile range over the 50 simulations. The dashed line corresponds to the ROC of a random classifier.

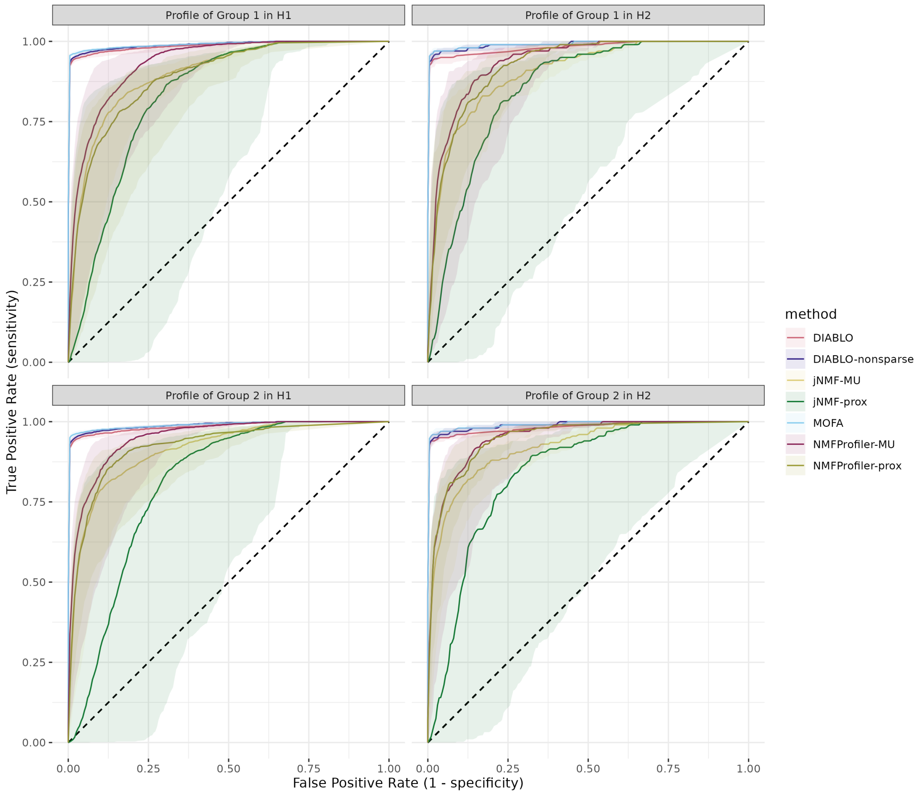

Figure S19: **Simulated dataset 06.** Median ROC curves. The ribbon corresponds to the interquartile range over the 50 simulations. The dashed line corresponds to the ROC of a random classifier.

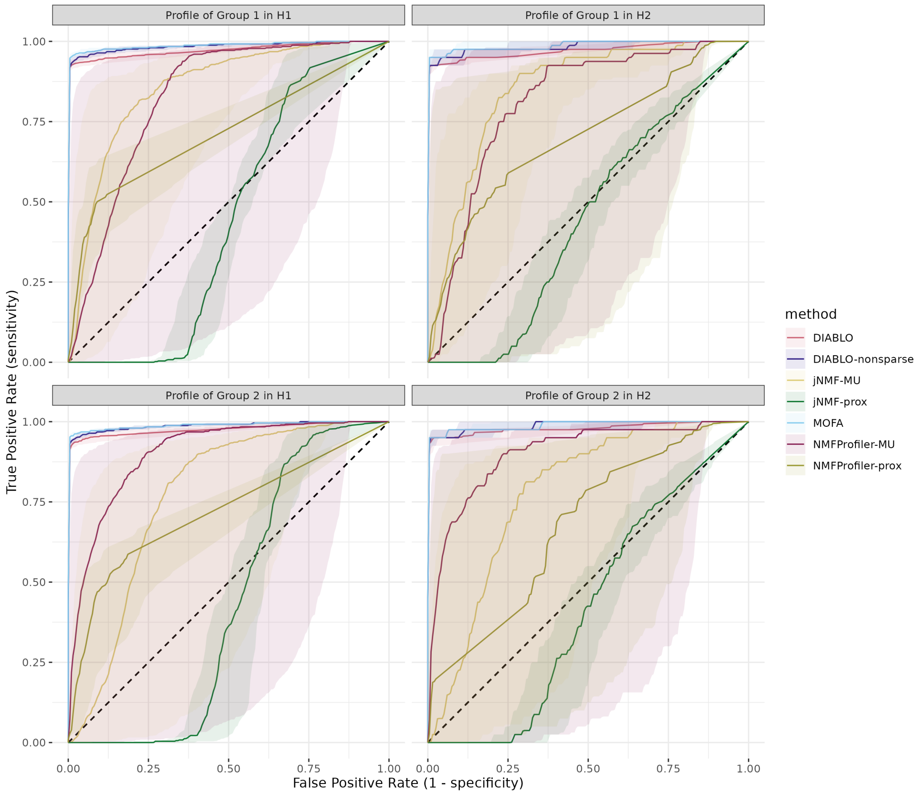

Figure S20: **Simulated dataset 07.** Median ROC curves. The ribbon corresponds to the interquartile range over the 50 simulations. The dashed line corresponds to the ROC of a random classifier.

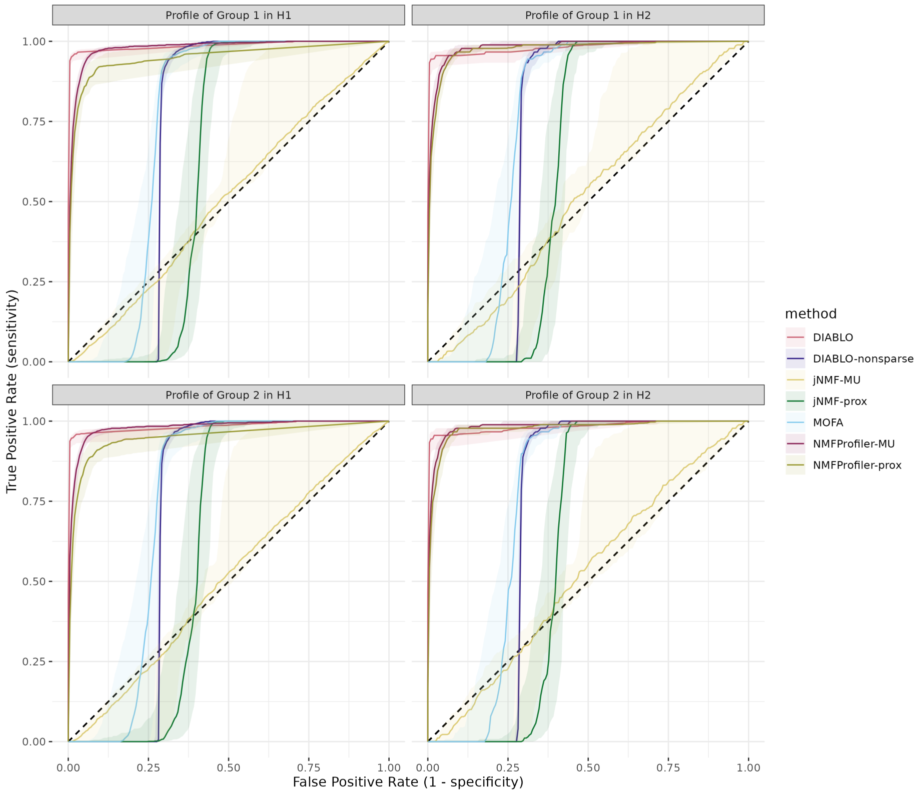

Figure S21: **Simulated dataset 08.** Median ROC curves. The ribbon corresponds to the interquartile range over the 50 simulations. The dashed line corresponds to the ROC of a random classifier.

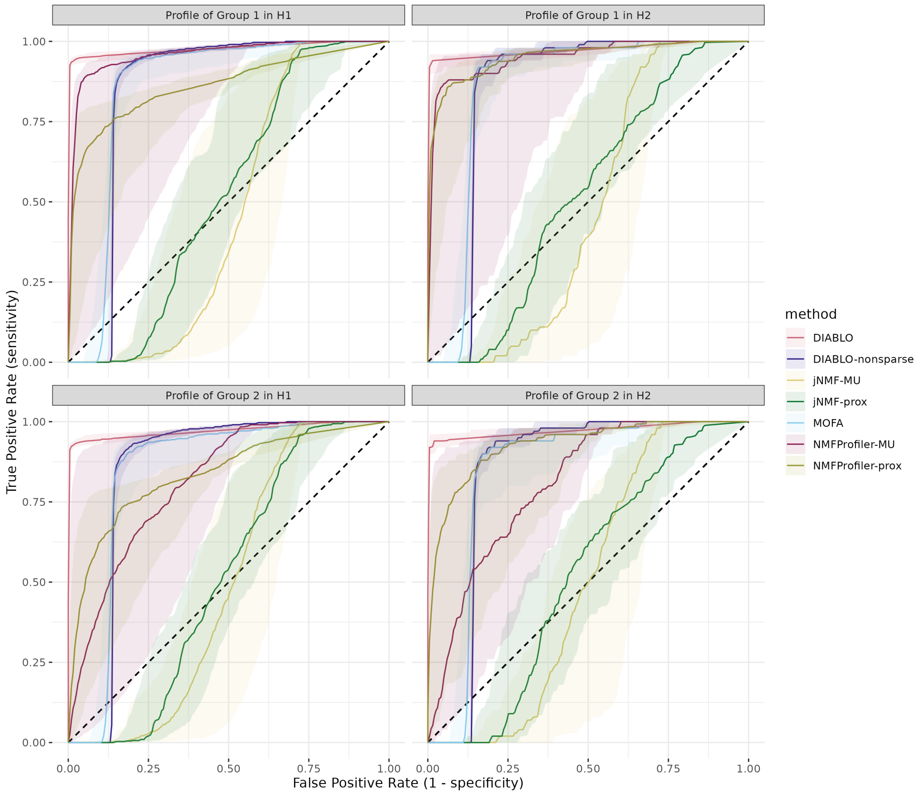

Figure S22: **Simulated dataset 09.** Median ROC curves. The ribbon corresponds to the interquartile range over the 50 simulations. The dashed line corresponds to the ROC of a random classifier.

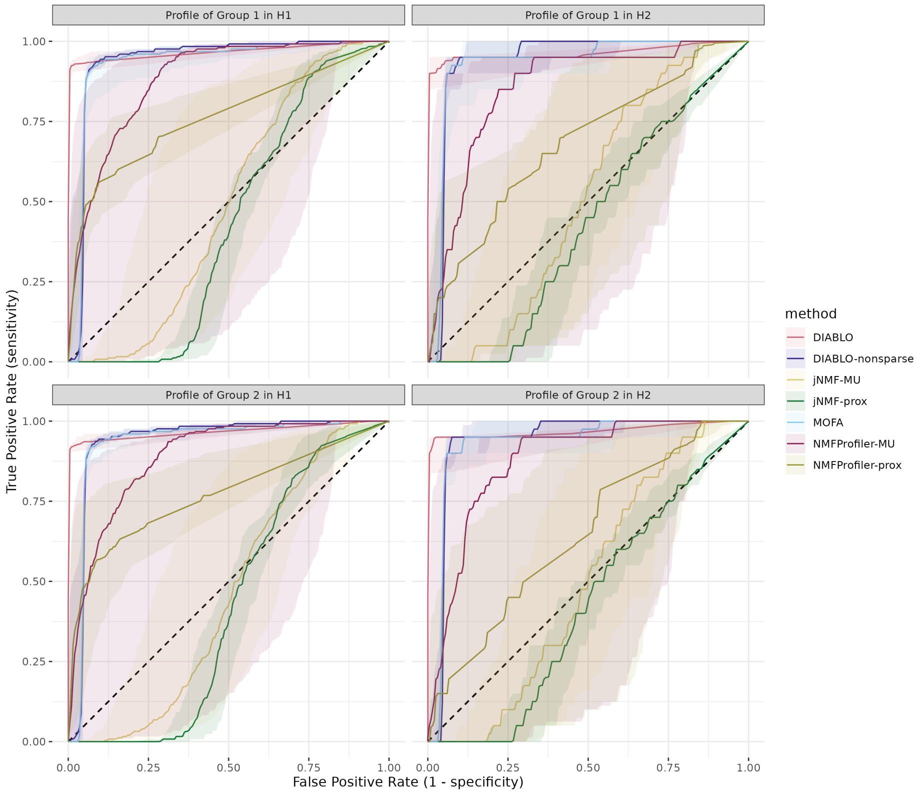

Figure S23: **Simulated dataset 10.** Median ROC curves. The ribbon corresponds to the interquartile range over the 50 simulations. The dashed line corresponds to the ROC of a random classifier.

Variation of the group sample sizes (column "Group size n_u")

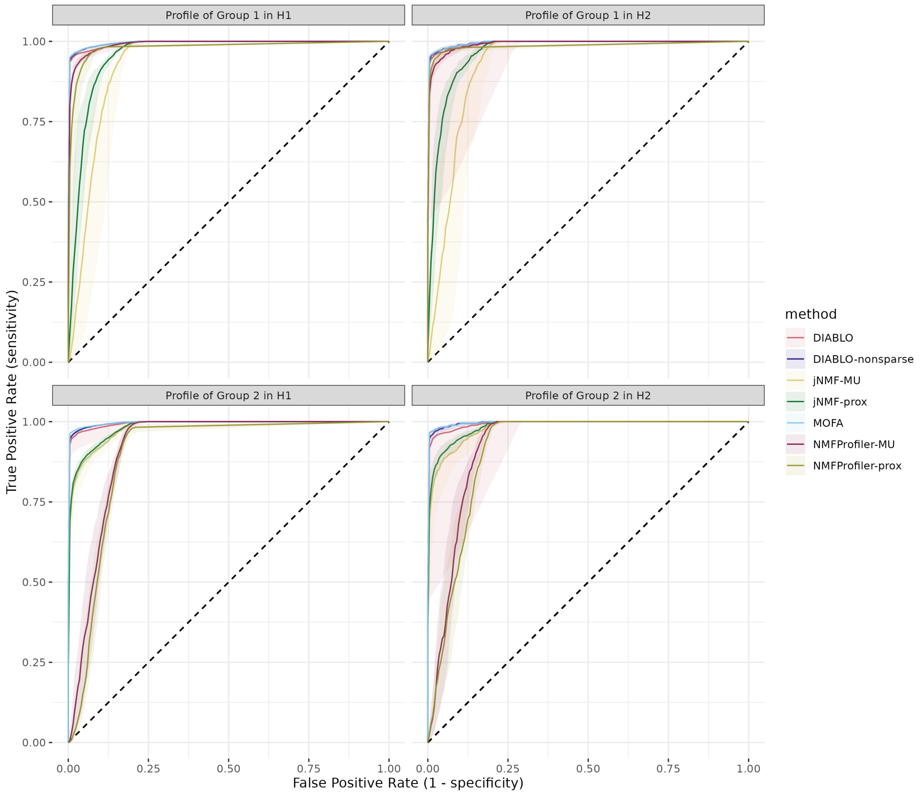

Figure S24: **Simulated dataset 11.** Median ROC curves. The ribbon corresponds to the interquartile range over the 50 simulations. The dashed line corresponds to the ROC of a random classifier.

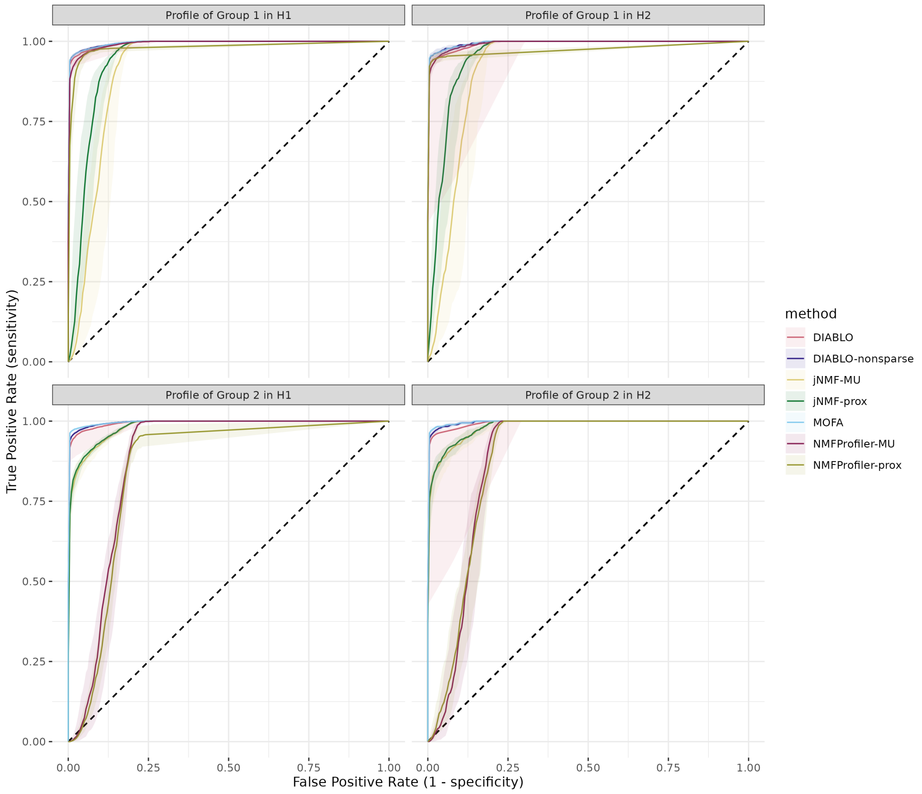

Figure S25: **Simulated dataset 12.** Median ROC curves. The ribbon corresponds to the interquartile range over the 50 simulations. The dashed line corresponds to the ROC of a random classifier.

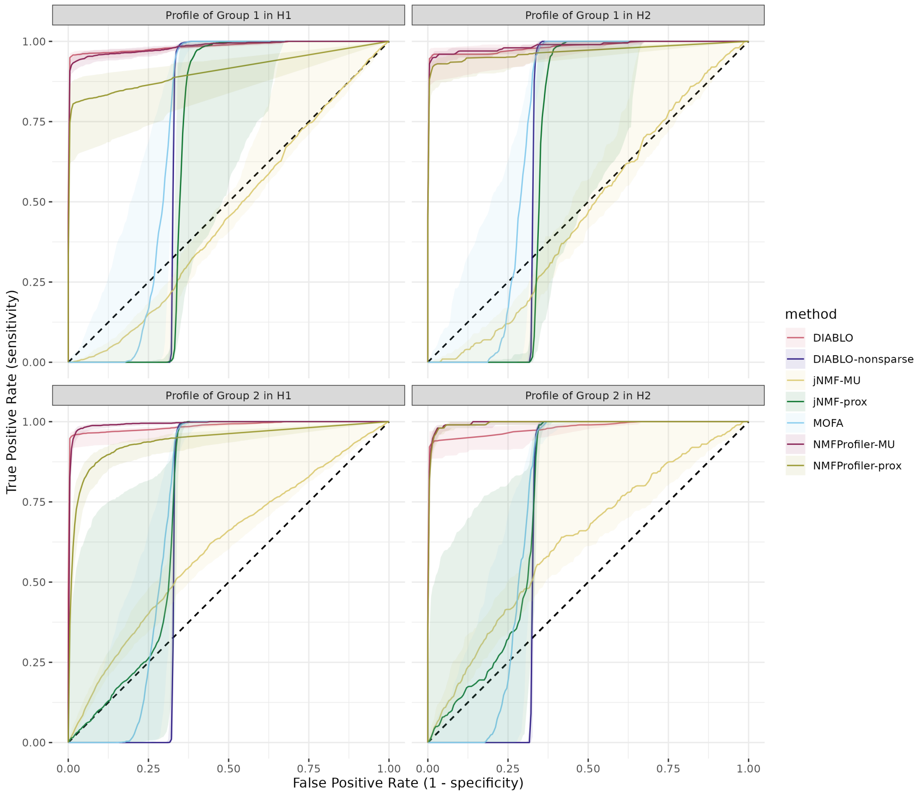

Figure S26: **Simulated dataset 13.** Median ROC curves. The ribbon corresponds to the interquartile range over the 50 simulations. The dashed line corresponds to the ROC of a random classifier.

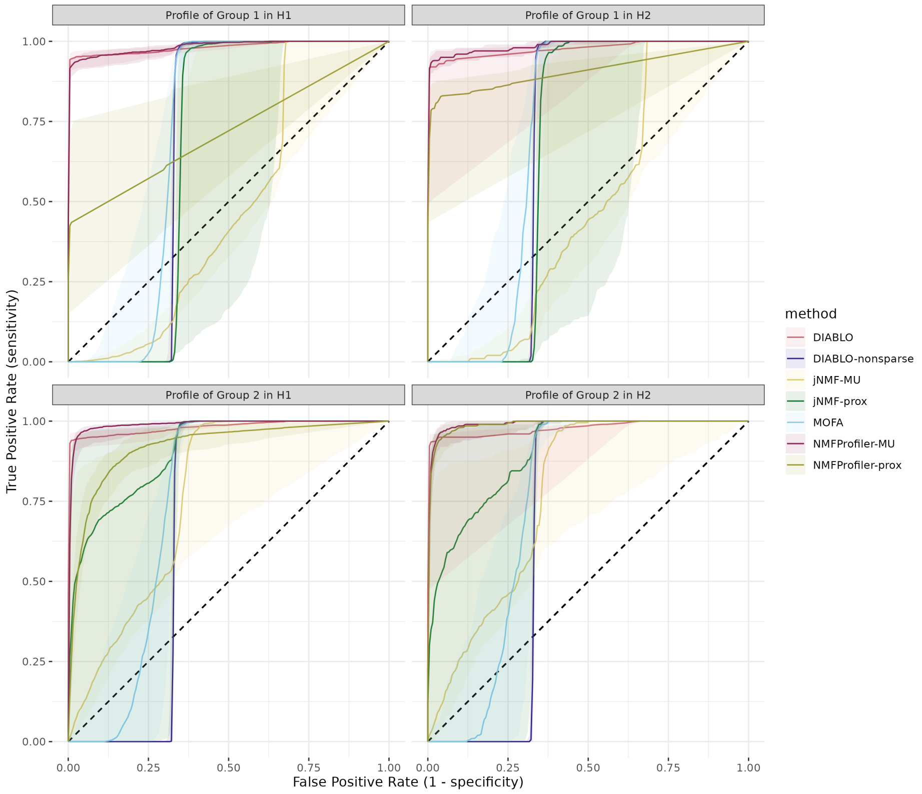

Figure S27: **Simulated dataset 14.** Median ROC curves. The ribbon corresponds to the interquartile range over the 50 simulations. The dashed line corresponds to the ROC of a random classifier.

S5.3 Additional methods and results for TCGA-COAD

S5.3.1 Specific choices in the implementation of NMFProfiler and DIABLO

NMFProfiler. We chose to use NMFProfiler-MU with default hyper-parameters because it was shown more robust and appropriate to very high-dimensional datasets on simulated data than NMFProfiler-Prox. Obtained signatures were found close to sparsity (with many coefficients close to zero) and were rendered exactly sparse using the threshold obtained with the brokenstick heuristic (see Figure S28). Obtained signature sizes are given in Table S4.

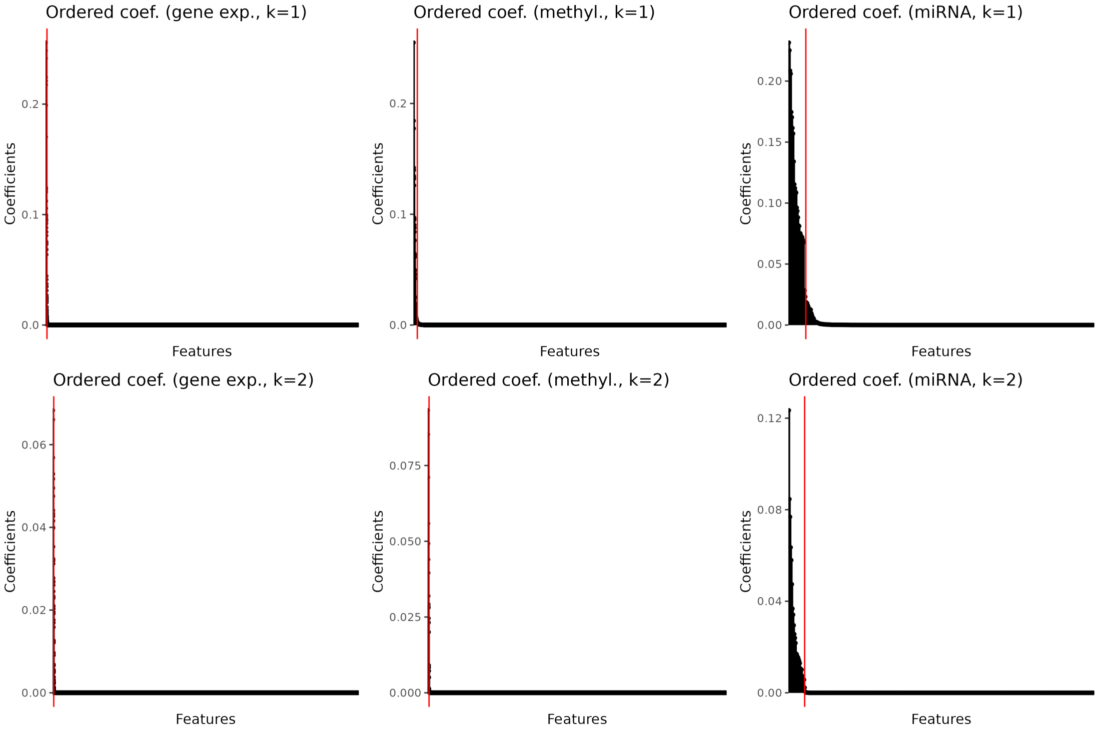

Figure S28: **COAD.** Hard thresholding on N0vsN1 signatures obtained with NMFProfiler-MU, for each omic and each component $k \in \{1, 2\}$. Coefficients are sorted in decreasing order. Red vertical line corresponds to the threshold obtained with the brokenstick heuristic.

Table S4: **COAD.** NMFProfiler-MU signature sizes obtained by the brokenstick heuristic for each (recoded) clinical feature and group

Clinical feature	Group	`gene` ($p_{\texttt{gene}} = 18877$)	`methyl` ($p_{\texttt{methyl}} = 5000$)	`miRNA` ($p_{\texttt{miRNA}} = 503$)
T	T2	36	23	17
	T3	86	78	28
	T4	41	14	16
	All	**161**	**115**	**60**
T2vsT3	T2	34	22	22
	T3	115	74	38
	All	**149**	**96**	**60**
T2vsT4	T2	40	22	20
	T4	47	23	20
	All	**87**	**45**	**40**
M	M0	97	87	40
	M1	37	13	14
	MX	43	26	16
	All	**176**	**126**	**68**
M0vsM1	M0	150	105	44
	M1	39	16	15
	All	**189**	**121**	**58**
M0vsMX	M0	93	103	36
	MX	52	26	19
	All	**145**	**129**	**55**
N	N0	64	60	31
	N1	52	26	23
	N2	51	22	18
	All	**167**	**106**	**69**
N0vsN1	N0	81	62	29
	N1	60	22	27
	All	**141**	**84**	**56**
N0vsN2	N0	79	57	31
	N2	50	14	22
	All	**129**	**71**	**53**

DIABLO. Cross-validation was too long to run for DIABLO to choose the number of selected features in the components. Hence, for a fair comparison we selected the same number of features than the one obtained for NMFProfiler.

S5.3.2 Cox model to explain survival

We assess the ability of DIABLO and NMFProfiler-MU signatures to be predictive of survival, similarly to what was done in [Cantini et al., 2021]. For a given clinical feature $\mathbf{y}$ and for each omic $j \in \{\texttt{gene}, \texttt{methyl}, \texttt{miRNA}\}$, we computed $\mathbf{X}^{(j)}\widehat{\mathbf{H}}^{(j)\top} \in \mathbb{R}^{n \times K}$, the projection of samples onto signatures. Then, for each group u in $\mathbf{y}$, we extracted the submatrix of $\mathbf{X}^{(j)}\widehat{\mathbf{H}}^{(j)\top}$, specific of this group (*i.e.*, rows corresponding to samples in group u and columns corresponding to the signature k describing group u). The $J = 3$ submatrices of group u were then concatenated to form a three-dimensional matrix of predictors for this group.

Finally, a Cox proportional hazard model was fitted to estimate the survival time before death event from this matrix (`coxph()` function of R package **survival**). Three omic-specific p-values and a global model p-value were obtained corresponding, respectively, to the results of the Wald test for each column and to the likelihood ratio test of the full model against the empty model.

Results for N

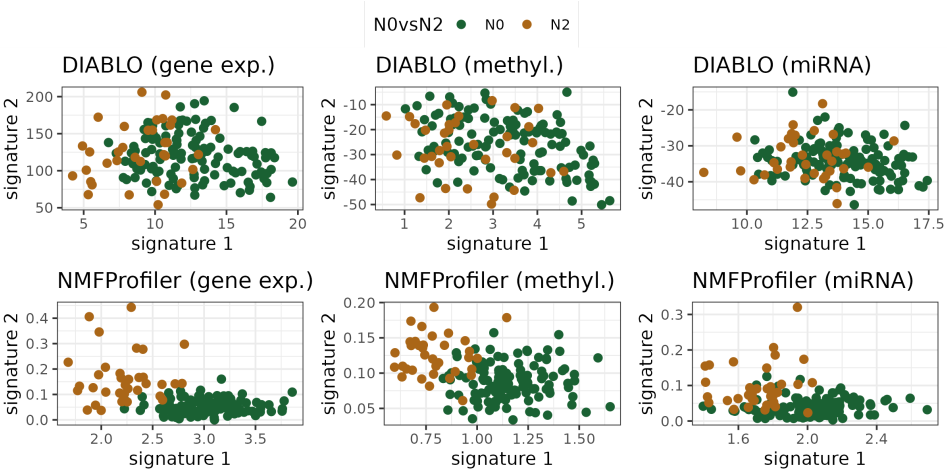

Figure S29: **COAD.** Projections of samples onto signatures of N0vsN2 for each omic and method. For DIABLO, only the x-axis is relevant.

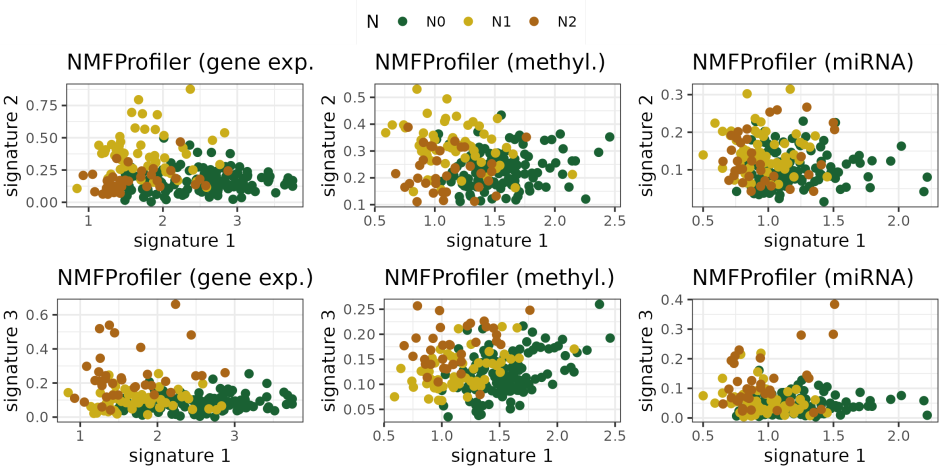

Figure S30: **COAD.** Projections of samples onto signatures of N obtained by NMFProfiler for each omic. The first signature corresponds to group N0, the second to group N1 and the third to group N2.

Results for M

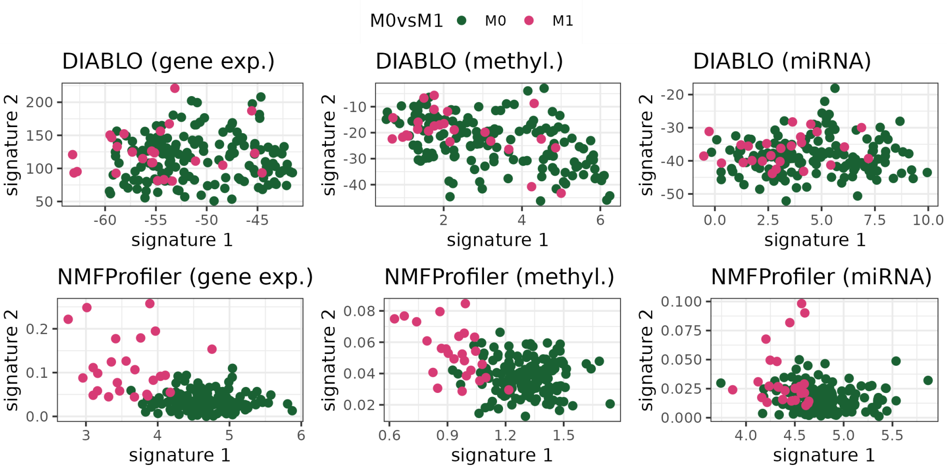

Figure S31: **COAD.** Projections of samples onto signatures of M0vsM1 for each omic and method. For DIABLO, only the x-axis is relevant.

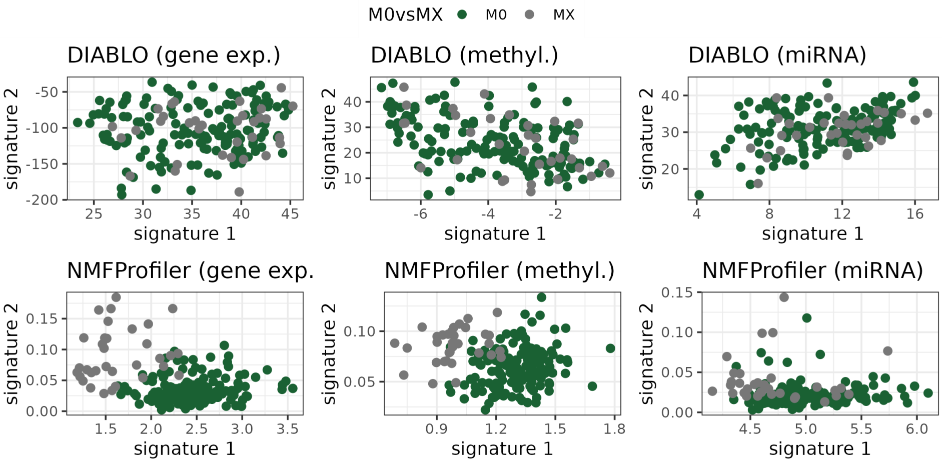

Figure S32: **COAD.** Projections of samples onto signatures of M0vsMX for each omic and method. For DIABLO, only the x-axis is relevant.

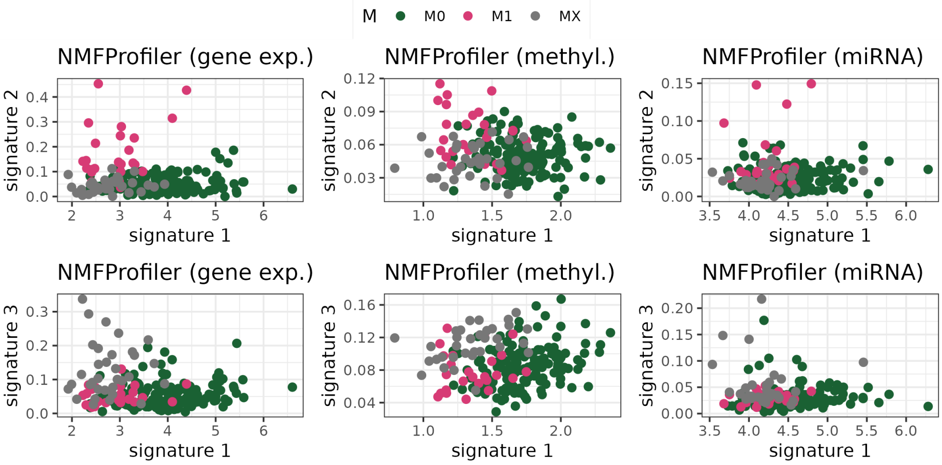

Figure S33: **COAD.** Projections of samples onto signatures of M obtained by NMFProfiler for each omic. The first signature corresponds to group M0, the second to group M1 and the third to group MX.

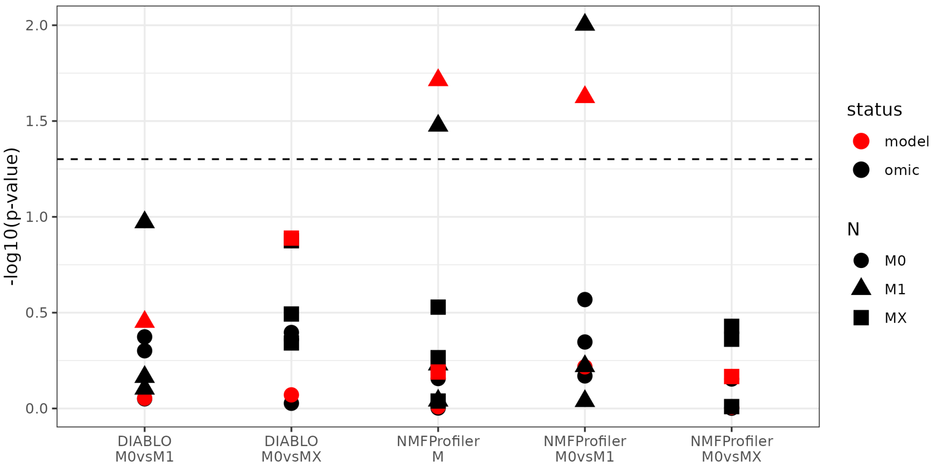

Figure S34: **TCGA-COAD.** $-\log_{10}(p\text{-values})$ obtained with Cox proportional hazard model for M0vsM1 and M0vsMX association with DIABLO and NMFProfiler. Model p-value is displayed in red and p-values related to a specific omic signature are displayed in black. The dashed horizontal line corresponds to 0.05.

Results for T

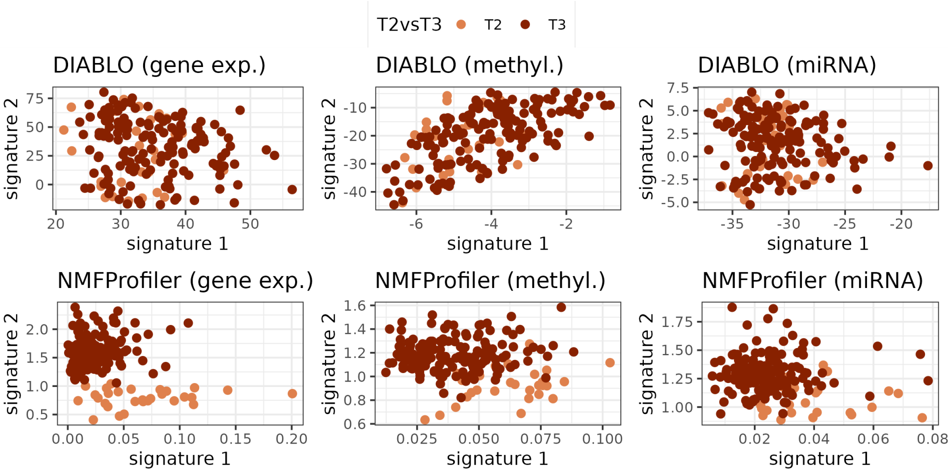

Figure S35: **COAD.** Projections of samples onto signatures of T2vsT3 for each omics and method. For DIABLO, only the x-axis is relevant.

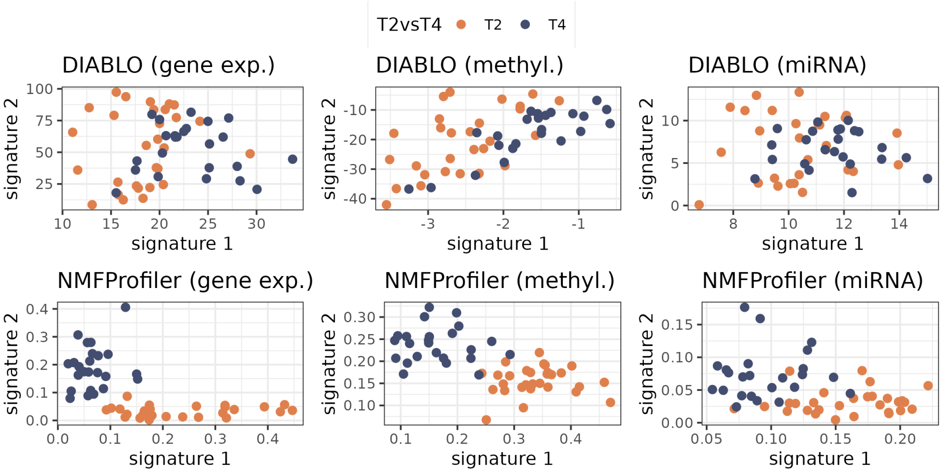

Figure S36: **COAD.** Projections of samples onto signatures of T2vsT4 for each omic and method. For DIABLO, only the x-axis is relevant.

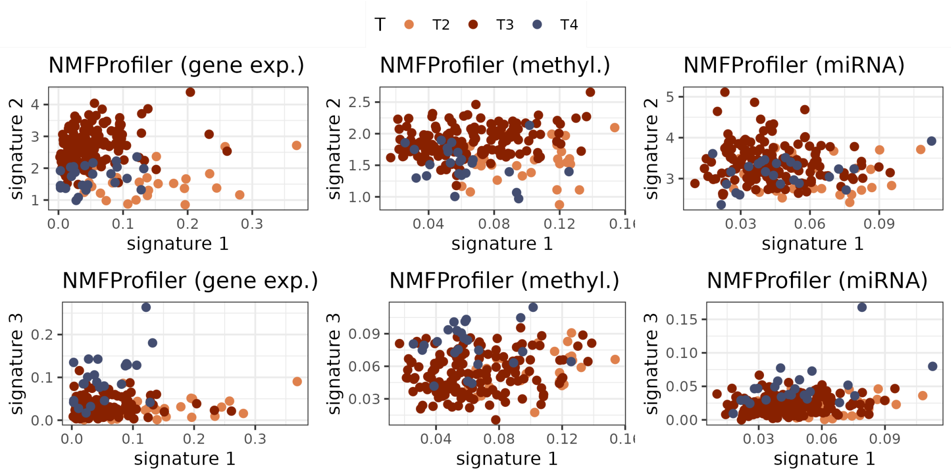

Figure S37: **COAD.** Projections of samples onto signatures of T obtained by NMFProfiler for each omic. The first signature corresponds to group T2, the second to group T3 and the third to group T4.

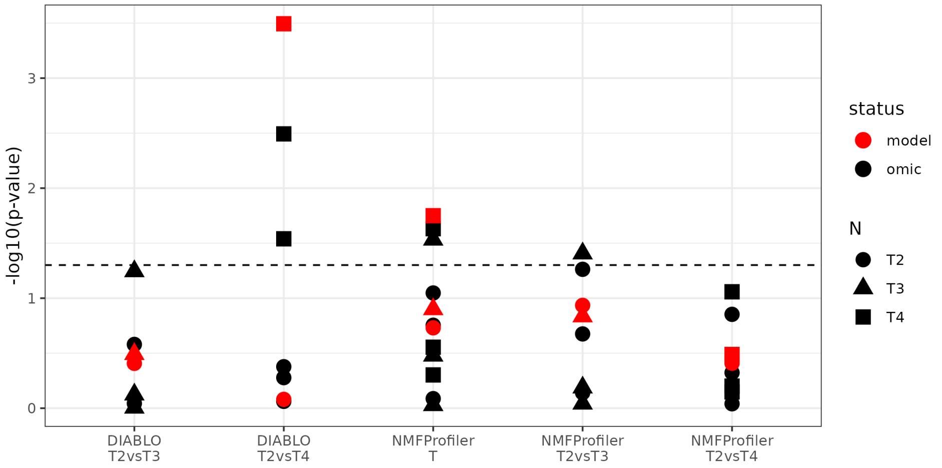

Figure S38: **TCGA-COAD.** $-\log_{10}(p\text{-values})$ obtained with Cox proportional hazard model for T2vsT3 and T2vsT4 association with DIABLO and NMFProfiler. Model p-value is displayed in red and p-values related to a specific omic signature are displayed in black. The dashed horizontal line corresponds to 0.05.

S5.4 Additional methods and results for AD study

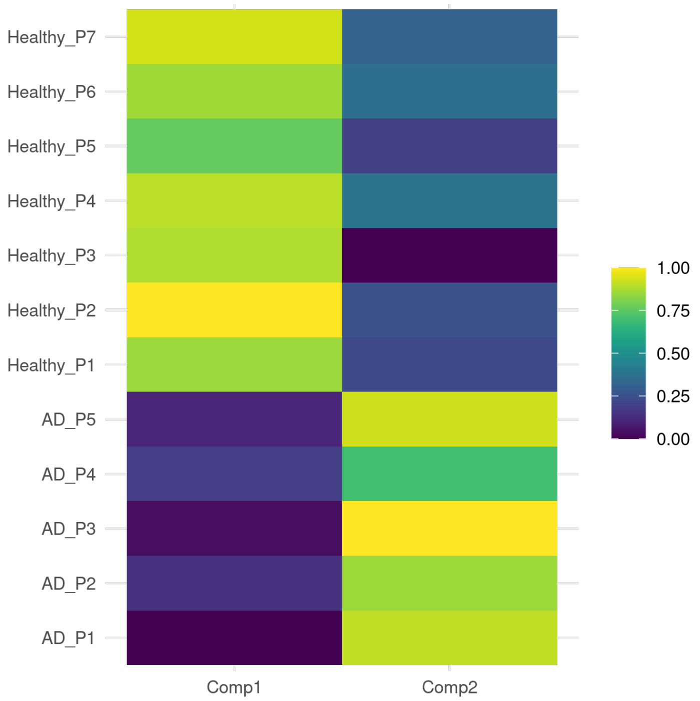

Figure S39: **AD study.** Heatmap of the estimation of the contribution matrix $\mathbf{W} \in \mathbb{R}^{12\times 2}$. To ease readability, coefficients have been rescaled so that their maximum is equal to 1.

Differential analysis on single omics As a baseline for comparison, differential analysis was performed for proteomic and transcriptomic data independently. Results were obtained using all samples from the left and right arms and including samples corresponding to subjects for which only proteomic or transcriptomic was available (*e.g.*, $n = 26$ for proteomic and $n = 28$ for transcriptomic instead of $n = 12$ for the multi-omic analysis; see Section S3.3 for further details). In addition, transcriptomic differential analysis was based on unfiltered probeset (*i.e.*, $p_1 = 22,557$ instead of $p_1 = 1,847$ for the multi-omic analysis). In addition, two proteins were not included in the differential analysis due to low expression (*i.e.*, $p_2 = 279$ instead of $p_2 = 281$ for the multi-omic analysis).

Differential analysis was performed using the linear model with moderated variance from the R package **limma** [Ritchie et al., 2015]. The correlation between the 2 samples corresponding to the same subject (left and right arms) was taken into account with the `duplicateCorrelation` function. FDR control was ensured using the Benjamini and Hochberg [Benjamini and Hochberg, 1995] multiple test correction. A protein or a gene were declared differentially expressed if their adjusted p-value was below 5%.

Supplementary Table S1 gives the test statistic, p-value, and adjusted p-value for all features. 19 proteins were found differentially expressed between groups. Among proteins included in the healthy signature of NMFProfiler, eight out of 281 were also found differentially expressed and overexpressed for healthy samples: P05089_ARG1, P01023_A2M, P31944_CASP14, P20930_FLG, Q3SYB4_SERPINB12, Q13510_ASAH1, P02749_APOH, and P01871_IGHM. Among proteins included in the AD signature of NMFProfiler, eight out of 281 were also found differentially expressed and overexpressed for AD samples: P22528_SPRR1B, P13639_EEF2, Q01469_FABP5, O75083_WDR1, P14735_IDE, O75369_FLNB, P22531_SPRR2E, and P06733_ENO1.

903 genes were found differentially expressed between groups. Among genes included in the healthy signature of NMFProfiler, four out of $1,847$ were found differentially expressed and overexpressed for healthy samples: *RPL10A*, *HNRNPH1*, *ASS1*, and *SOX15*. Among genes included in the AD signature of NMFProfiler, five out of $1,847$ were found differentially expressed and overexpressed for AD samples: *ATP6V1A*, *MOB3A*, *STEAP4*, *GID8*, and *USP17L5*.

Comparison between NMFProfiler and DIABLO signatures DIABLO was also used on the a signature characterizing both healthy and non lesional AD profiles. Cross-validation for number of selected features was performed over $\{10, 25, 50, 75, 100, 200, 300, 400, 500\}$ and $\{10, 25, 50, 75, 100, 125, 150\}$, respectively for transcriptomics and proteomics. The best model corresponded to signature composed of 200 genes and 10 proteins. DIABLO signature was finally split in two group-specific signatures according to sign.

Figure S40 displays Venn diagrams between features included in NMFProfiler and DIABLO signatures. All the proteins selected by DIABLO were also selected by NMFProfiler, whatever the group. Also, a majority of genes selected by NMFProfiler were selected by DIABLO: For genes characteristic of the healthy skin group, five out of eight selected by NMFProfiler were also selected by DIABLO (the non consensual genes were *COX8A*, *HDLBP*, and *RPL10A*). For genes characteristic of the AD group, seven out of eight selected by NMFProfiler were also selected by DIABLO (the non consensual gene was *STEAP4*). Overall, NMFProfiler is less influenced by unbalanced sizes between the two datasets in its selection, while DIABLO tends to extract signatures with sizes more influenced by the respective initial number of features of the two omics.

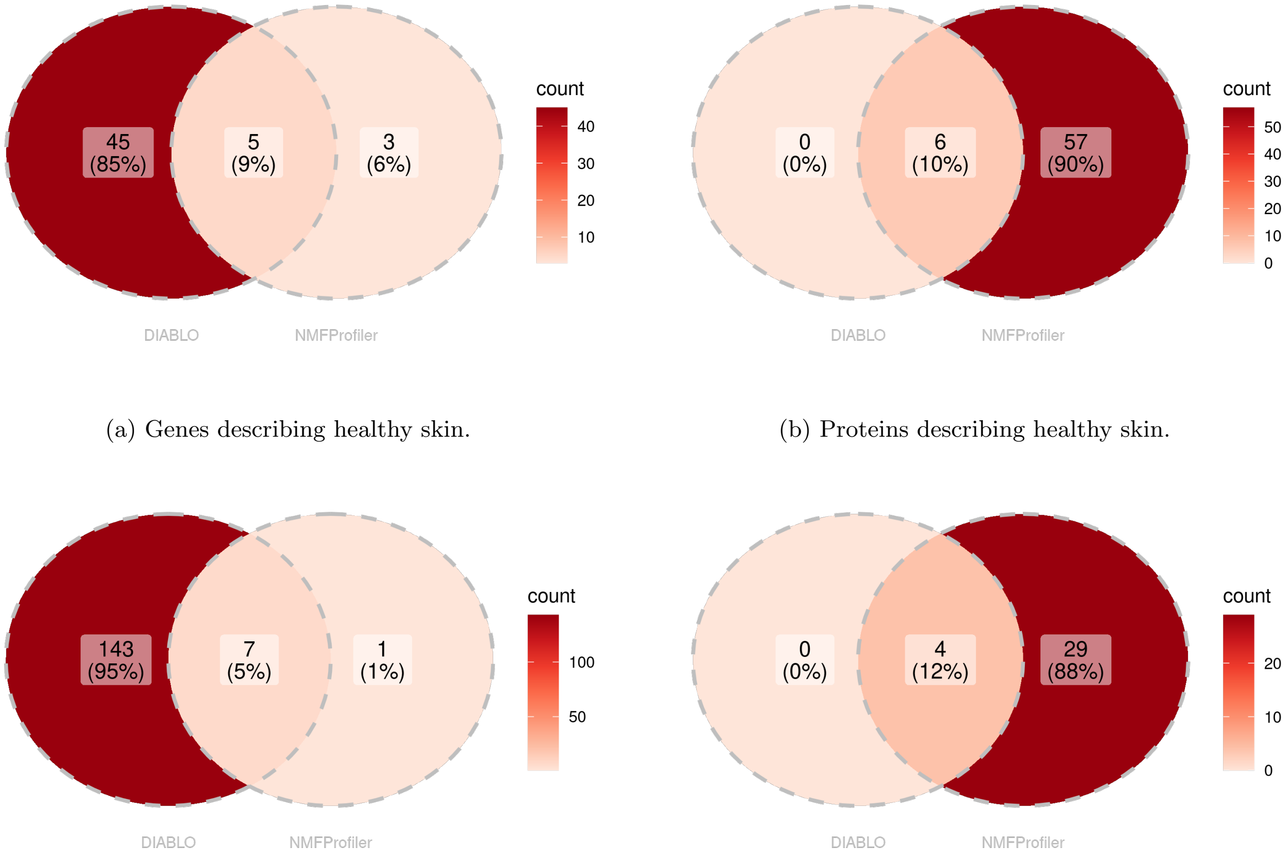

(a) Genes describing healthy skin.

(b) Proteins describing healthy skin.

(c) Genes describing non lesional AD skin.

(d) Proteins describing non lesional AD skin.

Figure S40: **AD study.** Venn diagrams of signatures obtained with DIABLO (left) and NMFProfiler-prox (right), for each group and omic.

The strength of correlation between features selected in a given signature was assessed through the average (absolute

value of) Pearson pairwise correlations (Table S5). As expected, DIABLO provides signatures with very strong correlations between features: In particular, DIABLO gene signature has an average (absolute value of) correlation equal to 0.682 while the (smaller) gene signatures of NMFProfiler are less redundant, with an average absolute value of correlations equal to 0.51. This is explained by the fact that the objective function of DIABLO, based on covariance, favors strong correlations between extracted features, while NMFProfiler seeks good reconstruction and better benefit from the ℓ_1 penalty to extract non redundant features. The same remark holds for protein signatures (average signature of 0.729 for DIABLO and of 0.369 for NMFProfiler), even though more expected considering the signature sizes.

Table S5: **AD study.** Average absolute value of Pearson coefficient correlations for each method and omic.

Method	Transcriptomics	Proteomics	Both
DIABLO	0.682	0.729	0.681
NMFProfiler	0.505	0.369	0.388

S6 List of figures, tables and references

List of Figures

S1 Solutions of the least-square problems in the case of the standard LDA ($\boldsymbol{\beta}_F$) and of the nonnegative constrained LDA ($\boldsymbol{\beta}_{\text{FR-lda}}$) as used in FR-lda. 2
S2 Illustration of the supervised part LDA of NMFProfiler. 3
S3 **Simulated dataset 02.** Heatmaps of $\mathbf{X}^{(j)}$. 12
S4 **Simulated dataset 02.** Individual ROC curves for DIABLO. 13
S5 **Simulated dataset 02.** Individual ROC curves for NMFProfiler-MU. 13
S6 **Simulated dataset 02.** Individual ROC curves for NMFProfiler-prox. 14
S7 **Simulated dataset 02.**. Specificity of NMFProfiler variants. 14
S8 **Simulated dataset 02.** Specificity of DIABLO variants. 15
S9 **Simulated dataset 02.** Distribution of coefficients in dictionary matrices $\mathbf{H}^{(j)}$ for dataset 1, group 1 for NMFProfiler variants. 15
S10 **Simulated dataset 02.** Classification accuracy in logistic regression from features selected by supervised methods. 16
S11 **Simulated dataset 02.** McFadden index obtained in logistic regression from features selected by supervised methods. 16
S12 **Simulated dataset 02.** Computational time of the different methods. 17
S13 **Simulated dataset 00.** Median ROC curves. 21
S14 **Simulated dataset 01.** Median ROC curves. 22
S15 **Simulated dataset 03.** Median ROC curves. 22
S16 **Simulated dataset 04.** Median ROC curves. 23
S17 **Simulated dataset 05.** Median ROC curves. 23
S18 **Simulated dataset 05.** Median ROC curves for the different supervised NMF variants. 24
S19 **Simulated dataset 06.** Median ROC curves. 24
S20 **Simulated dataset 07.** Median ROC curves. 25
S21 **Simulated dataset 08.** Median ROC curves. 25
S22 **Simulated dataset 09.** Median ROC curves. 26
S23 **Simulated dataset 10.** Median ROC curves. 26
S24 **Simulated dataset 11.** Median ROC curves. 27
S25 **Simulated dataset 12.** Median ROC curves. 27
S26 **Simulated dataset 13.** Median ROC curves. 28
S27 **Simulated dataset 14.** Median ROC curves. 28
S28 **TCGA-COAD.** Hard thresholding on N0vsN1 signatures. 29
S29 **TCGA-COAD.** Projections of samples onto signatures of N0vsN2. 31
S30 **TCGA-COAD.** Projections of samples onto signatures of N. 31
S31 **TCGA-COAD.** Projections of samples onto signatures of M0vsM1. 31
S32 **TCGA-COAD.** Projections of samples onto signatures of M0vsMX. 32
S33 **TCGA-COAD.** Projections of samples onto signatures of M. 32
S34 **TCGA-COAD.** $-\log_{10}(p\text{-values})$ obtained with Cox proportional hazard model for M0vsM1 and M0vsMX association with DIABLO and NMFProfiler. 32
S35 **TCGA-COAD.** Projections of samples onto signatures of T2vsT3. 33
S36 **TCGA-COAD.** Projections of samples onto signatures obtained of T2vsT4. 33
S37 **TCGA-COAD.** Projections of samples onto signatures of T. 33
S38 **TCGA-COAD.** $-\log_{10}(p\text{-values})$ obtained with Cox proportional hazard model for T2vsT3 and T2vsT4 association with DIABLO and NMFProfiler. 34
S39 **AD study.** Heatmap of the estimation of the contribution matrix $\mathbf{W}$. 34
S40 **AD study.** Venn diagrams of signatures obtained with DIABLO and NMFProfiler-prox. 35

List of Tables

S1 **Simulated data.** Settings used to generate the different simulated datasets. 10
S2 **TCGA-COAD.** Group sizes of recoded clinical features. 11
S3 **Simulated data.** Averaged AUROCs given simulation scenario number, dataset and group. 18
S4 **TCGA-COAD.** NMFProfiler-MU signature sizes. 30
S5 **AD study.** Average absolute value of Pearson coefficient correlations for each method and omic. . . . 36

References

[Bauschke and Combettes, 2017] Bauschke, H. H. and Combettes, P. L. (2017). *Convex Analysis and Monotone Operator Theory in Hilbert Spaces.* CMS Books in Mathematics. Springer International Publishing, Cham, Switzerland.

[Benjamini and Hochberg, 1995] Benjamini, Y. and Hochberg, Y. (1995). Controlling the false discovery rate: A practical and powerful approach to multiple testing. *Journal of the Royal Statistical Society: Series B (Methodological)*, 57(1):289–300.

[Brouard et al., 2022] Brouard, C., Mariette, J., Flamary, R., and Vialaneix, N. (2022). Feature selection for kernel methods in systems biology. *NAR Genomics and Bioinformatics*, 4(1):lqac014.

[Cantini et al., 2021] Cantini, L., Zakeri, P., Hernandez, C., Naldi, A., Thieffry, D., Remy, E., and Baudot, A. (2021). Benchmarking joint multi-omics dimensionality reduction approaches for the study of cancer. *Nature Communications*, 12(1):124.

[Duda et al., 2000] Duda, R. O., Hart, P. E., and Stork, D. G. (2000). *Pattern Classification.* Willey-Blackwell, USA. 2nd edition.

[Fernsel and Maass, 2018] Fernsel, P. and Maass, P. (2018). A survey on surrogate approaches to non-negative matrix factorization. *Vietnam Journal of Mathematics*, 46:987–1021.

[Irizarry et al., 2003] Irizarry, R. A., Hobbs, B., Collin, F., Beazer-Barclay, Y. D., Antonellis, K. J., Scherf, U., and Speed, T. P. (2003). Exploration, normalization, and summaries of high density oligonucleotide array probe level data. *Biostatistics (Oxford, England)*, 4(2):249–264.

[Johnson et al., 2007] Johnson, W. E., Li, C., and Rabinovic, A. (2007). Adjusting batch effects in microarray expression data using empirical bayes methods. *Biostatistics (Oxford, England)*, 8(1):118–127.

[Leuschner et al., 2019] Leuschner, J., Schmidt, M., Fernsel, P., Lachmund, D., Boskamp, T., and Maass, P. (2019). Supervised non-negative matrix factorization methods for MALDI imaging applications. *Bioinformatics*, 35:1940–1947.

[Parikh and Boyd, 2014] Parikh, N. and Boyd, S. (2014). Proximal algorithms. *Foundations and Trends® in Optimization*, 1(3):127–239.

[Rappoport and Shamir, 2018] Rappoport, N. and Shamir, R. (2018). Multi-omic and multi-view clustering algorithms: review and cancer benchmark. *Nucleic Acids Research*, 46(20):10546–10562.

[Ritchie et al., 2015] Ritchie, M. E., Phipson, B., Wu, D., Hu, Y., Law, C. W., Shi, W., and Smyth, G. K. (2015). limma powers differential expression analyses for rna-sequencing and microarray studies. *Nucleic Acids Research*, 43(7):e47.
